# Small-molecule PROTAC mediates targeted protein degradation to treat STAT3-dependent epithelial cancer

**DOI:** 10.1172/jci.insight.160606

**Published:** 2022-11-22

**Authors:** Jinmei Jin, Yaping Wu, Zeng Zhao, Ye Wu, Yu-dong Zhou, Sanhong Liu, Qingyan Sun, Guizhu Yang, Jiayi Lin, Dale G. Nagle, Jiangjiang Qin, Zhiyuan Zhang, Hong-zhuan Chen, Weidong Zhang, Shuyang Sun, Xin Luan

**Affiliations:** 1 Shanghai Frontiers Science Center for Chinese Medicine Chemical Biology, Institute of Interdisciplinary Integrative Medicine Research, Shanghai University of Traditional Chinese Medicine, Shanghai, China.; 2 Department of Oral and Maxillofacial-Head Neck Oncology, Shanghai Ninth People’s Hospital, Shanghai Jiao Tong University School of Medicine, College of Stomatology, Shanghai Jiao Tong University, National Center for Stomatology, and; 3 National Clinical Research Center for Oral Diseases, Shanghai Key Laboratory of Stomatology, Shanghai, China.; 4 School of Pharmacy, Shanghai Jiao Tong University, Shanghai, China.; 5 China Institute of Pharmaceutical Industry, Shanghai, China.; 6 Department of Chemistry and Biochemistry, College of Liberal Arts, and; 7 Department of BioMolecular Sciences and Research Institute of Pharmaceutical Sciences, School of Pharmacy, University of Mississippi, University, Mississippi, USA.; 8 Zhejiang Provincial Research Center for Upper Gastrointestinal Tract Cancer, The Cancer Hospital of the University of Chinese Academy of Sciences (CAS), Zhejiang Cancer Hospital, Hangzhou, Zhejiang, China.

**Keywords:** Oncology, Therapeutics, Colorectal cancer, Drug therapy, Head and neck cancer

## Abstract

The aberrant activation of STAT3 is associated with the etiology and progression in a variety of malignant epithelial-derived tumors, including head and neck squamous cell carcinoma (HNSCC) and colorectal cancer (CRC). Due to the lack of an enzymatic catalytic site or a ligand-binding pocket, there are no small-molecule inhibitors directly targeting STAT3 that have been approved for clinical translation. Emerging proteolysis targeting chimeric (PROTAC) technology–based approach represents a potential strategy to overcome the limitations of conventional inhibitors and inhibit activation of STAT3 and downstream genes. In this study, the heterobifunctional small-molecule–based PROTACs are successfully prepared from toosendanin (TSN), with 1 portion binding to STAT3 and the other portion binding to an E3 ubiquitin ligase. The optimized lead PROTAC (TSM-1) exhibits superior selectivity, potency, and robust antitumor effects in STAT3-dependent HNSCC and CRC — especially in clinically relevant patient-derived xenografts (PDX) and patient-derived organoids (PDO). The following mechanistic investigation identifies the reduced expression of critical downstream STAT3 effectors, through which TSM-1 promotes cell cycle arrest and apoptosis in tumor cells. These findings provide the first demonstration to our knowledge of a successful PROTAC-targeting strategy in STAT3-dependent epithelial cancer.

## Graphical abstract

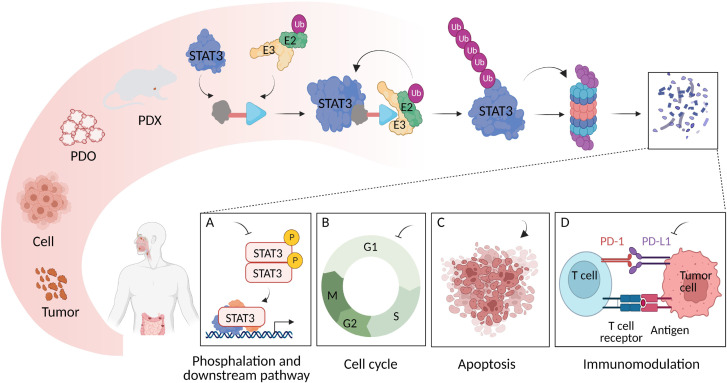

## Introduction

The STAT family of transcription factors has 7 known members, including STAT3, a key mediator of oncogenic signal transduction (1). In contrast to the transient mode of action in normal cells, STAT3 exhibits persistent aberrant activation in a variety of malignant epithelial-derived tumors (2). This is especially the case in over 95% of patients with head and neck squamous cell carcinoma (HNSCC) and 57.4% of patients with colorectal cancer (CRC) (3–5). Despite advancements in surgery and radiotherapy, and the integration of chemotherapy into treatment regimens, clinical HNSCC and CRC phenotypes are highly malignant, and 5-year survival rates remain poor (6–8). The lack of effective targeted therapies, poor response to immunotherapy, and resistance to chemotherapeutics pose significant treatment challenges (9, 10). Thus, new molecular target identification and subsequent development of therapeutic approach are essential to improve treatment outcome (11). Aberrant STAT3 activation represents an important early event during the development and progression of HNSCC and CRC (4, 8), promoting tumor cell proliferation, differentiation, invasion, angiogenesis (12), metastasis, and the evasion of detection by the immune system (13). Because the suppression of tumor cell apoptosis further contributes to reduced treatment response, the inhibition of aberrant STAT3 activation emerges as a compelling HNSCC and CRC target (8, 14, 15).

Strategies to block STAT3 activation in tumor cells include direct binding to the SH2 domain that is responsible for STAT3 dimerization and phosphorylation, or indirect inhibition of upstream tyrosine kinases (16). The compounds that directly bind to the SH2 domain include short peptide aptamers, peptidomimetics, and nonpeptidic small molecules (17). Challenges facing peptide aptamer and peptidomimetic drug development include reduced stability and poor cell permeability that limit their bioactivity (17). Nonpeptidic tyrosine kinase–targeted inhibitors have the potential to be developed as novel phosphorylated STAT3 inhibitors. However, their efficacy is diminished because the inherent transcriptional activity of unphosphorylated STAT3 could also drive the expression of oncogenes such as MET and MRAS by binding to NF-κB (18–21). Furthermore, the inhibition of STAT3 upstream signaling alone is insufficient to overcome the activation of compensatory signaling pathways (12, 13). Therefore, a strategy that completely inhibits the activity of STAT3 makes an invaluable addition to the arsenal of antitumor agents.

Specific downregulation or knockdown of STAT3 has recently emerged as a promising therapeutic approach, with the potential to overcome the limitations associated with conventional inhibitors. Antisense oligonucleotides that target STAT3 mRNA have entered preclinical studies in patients with HNSCC and shown initial efficacy; however, rapid degradation of mRNA significantly limits its bioavailability (14). Proteolysis targeting chimeric (PROTAC) technology has been widely used to specifically flag transcription factors for degradation (22). Unlike the occupancy mode of small molecule inhibitors, which increase the risk of off-target effects, PROTAC adopts the pattern of “event-driven” pharmacology binding at any point within the structure of potential targets (23, 24). Moreover, the catalytic nature of PROTAC can achieve and maintain enhanced target degradation at low doses (23, 25).

The PROTAC molecule SD-36 (26), developed by the Wang group with structure-based design, can effectively simulate the degradation of STAT3 protein in leukemia cells. Apart from the lengthy target-based drug discovery (TDD) design process, this method is envisioned as a feasible way to accelerate the development of a STAT3 degrader through a more phenotypic drug discovery (PDD) strategy (27, 28). Inspired by the chemical structure diversity and biological activity of natural products (29), it was proposed that PROTAC molecules should be designed based on natural molecules as a new strategy to effectively target STAT3, especially exploring the application of STAT3 degrader in solid tumors. Herein, PROTAC-based STAT3 degraders were successfully designed by coupling a small molecule toosendanin (TSN) that specifically binds STAT3 with an E3 ubiquitin ligase–targeted component to generate the chimeric molecule, referred to as TSM-1. The therapeutic potential and mechanisms of action of the PROTAC molecule TSM-1 in STAT3-dependent epithelial cancers (HNSCC and CRC) were systematically assessed in vitro and in vivo, and especially with the patient-derived xenografts (PDX) and patient-derived organoids (PDO) during the discovery of PROTAC molecules, which further indicated that TSM-1, as the first PROTAC molecule, may serve as a promising proteolytic enhancer for STAT3-dependent epithelial cancer.

## Results

### Expression of STAT3 and its clinical significance.

**Figure 1:**
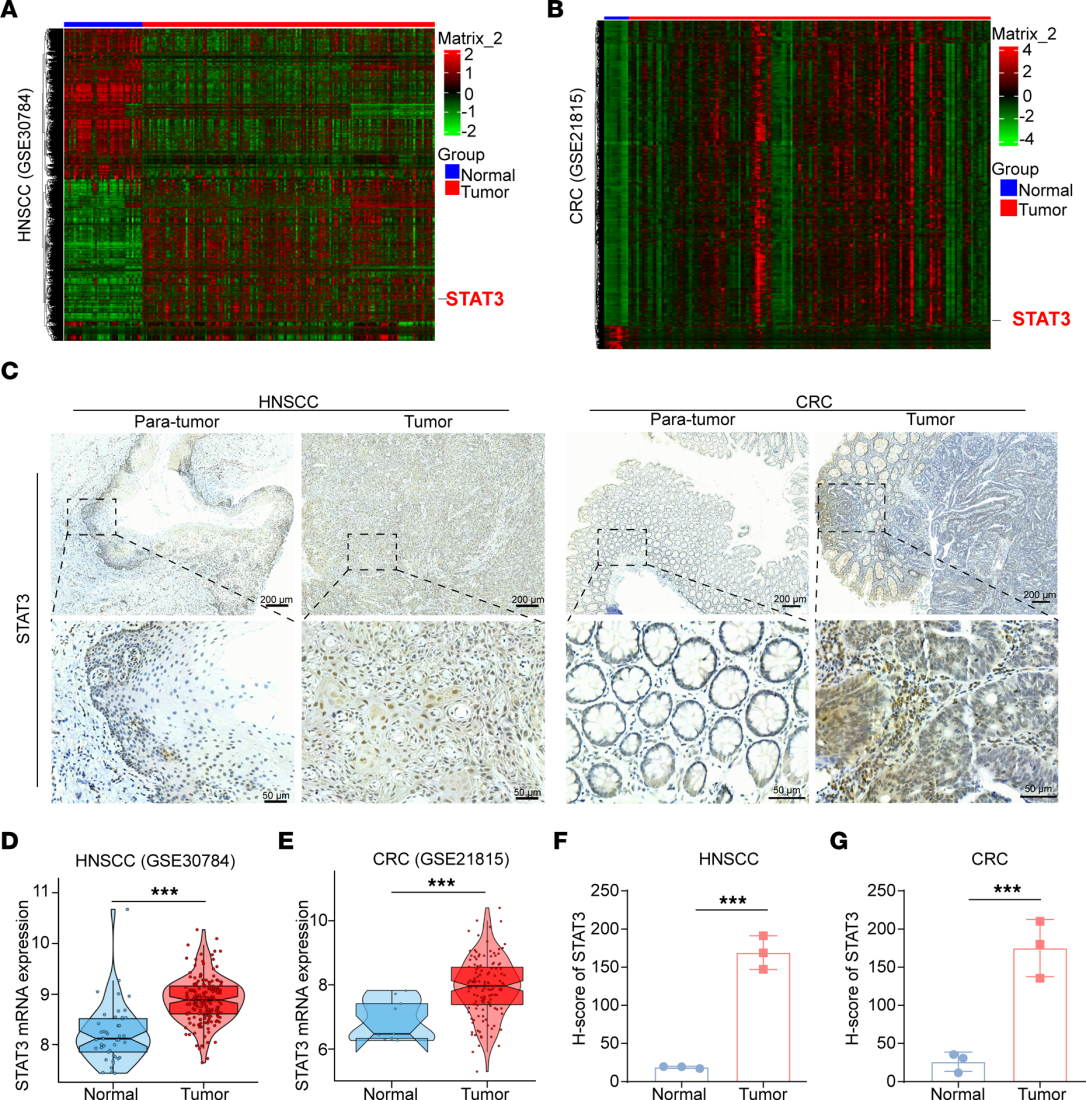
Expression of STAT3 and its clinical significance. (**A** and **D**) STAT3 expression was significantly increased in patients with HNSCC (GSE30784 database). (**B** and **E**) STAT3 expression was increased in patients with CRC according to the GSE21815 database. (**C**, **F**, and **G**) Increased expression of STAT3 protein was observed in biopsy samples from patients with HNSCC and patients with CRC (*n* = 3 patients). ***P < 0.001 when compared with the control group. *P* values are from 2-tailed paired *t* tests (**D**–**G**).

To establish the connection between STAT3 expression and disease stages, a bioinformatic-based approach was employed to analyze clinical sample–derived data deposited in the widely used GSE databases (30, 31). Analysis of the GSE database revealed that tumor samples expressed significantly higher levels of STAT3 in HNSCC (Figure 1, A and D) and CRC (Figure 1, B and E), in comparison with normal tissues. Examination of HNSCC and CRC (Figure 1, C, F, and G) patient biopsy specimens by IHC confirmed that the levels of STAT3 protein were significantly higher in tumors. Taken together, these results suggest that increased STAT3 expression and activation were highly associated with HNSCC and CRC progression and may serve as prognostic biomarkers and potential therapeutic targets.

### Rational design of TSM-1 as a potent degrader of STAT3 protein.

**Figure 2:**
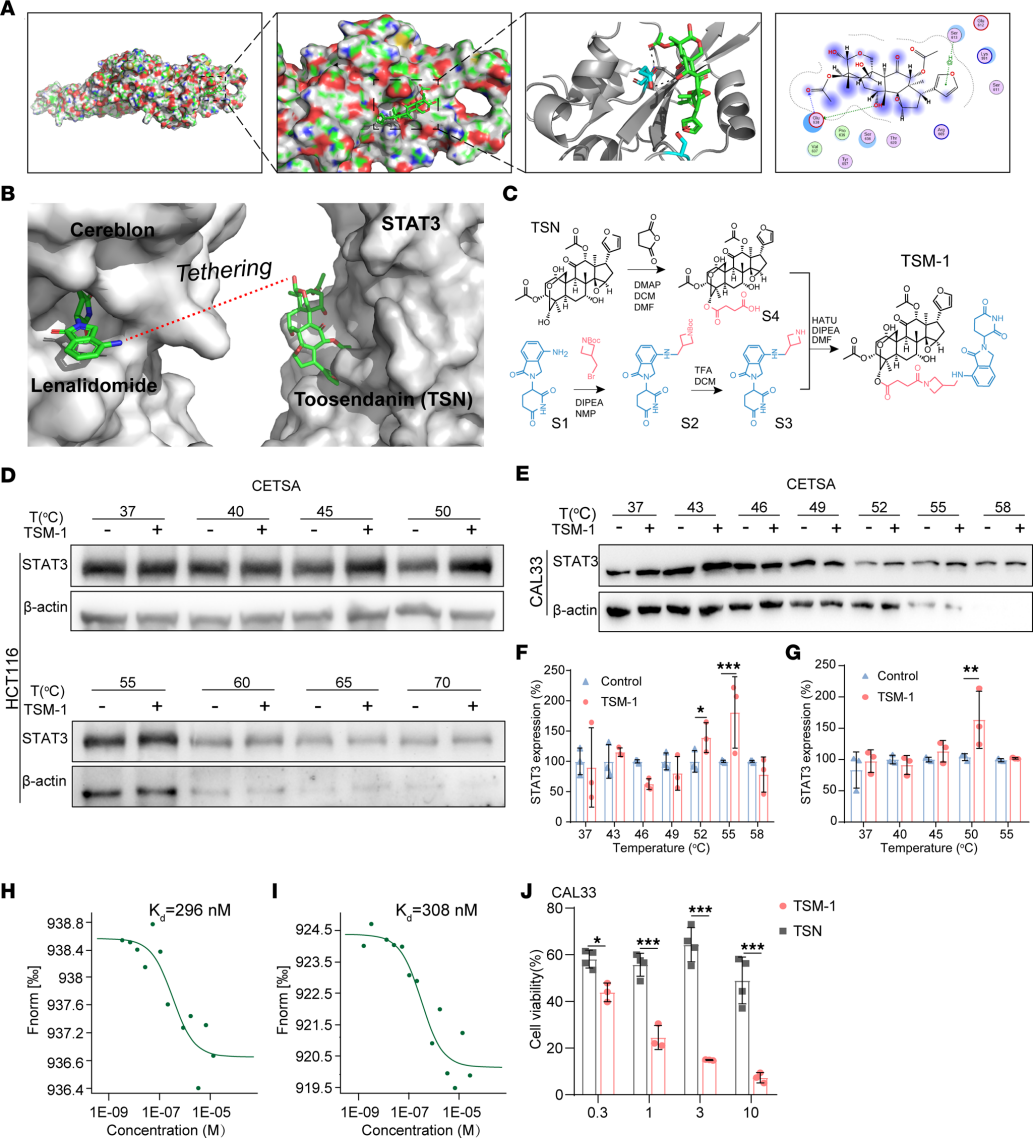
Design, synthesis, and screening of TSMs derivatives. (**A**) Computer simulation of the combination between TSN and STAT3 (PDB, 1BG1), where a hydrogen bond was formed between TSN and Glu638 or Ser613 on STAT3. (**B**) Computer simulation of appropriate site for tethering TSN to lenalidomide (4CI2) for the design of TSM-1. (**C**) Synthetic route of preparing TSM-1. (**D**–**G**) TSM-1 significantly increased the thermal stability of STAT3 in CETSA assays in CAL33 (52°C and 55°C) (**E** and **F**) and HCT116 (50°C) cells (**D** and **G**) (*n* = 3 replicates). (**H**) MST analysis of TSN binding to STAT3 (*K*_d_ = 296 nM). (**I**) MST analysis of TSM-1 binding to STAT3 (*K*_d_ = 308 nM). (**J**) CAL33 cells proliferation was detected using CCK-8 assays after treatment with TSN or TSM-1 for 48 hours (*n* = 3 replicates). Among them, the degrader TSM-1 exhibited pronounced antitumor effects. *P < 0.05, ** *P* < 0.01, and *** *P* < 0.001 when compared with the control group. *P* values are from 2-way ANOVA with Tukey’s multiple-comparison test (**F**, **G**, and **J**).

Following extensive literature-based research and multiple rounds of screening from our own library, the triterpenoid TSN, isolated from the traditional Chinese medicinal plant Melia toosendan, was selected as the STAT3 target warhead. TSN had demonstrated antitumor activity by targeting STAT3 regulation (32, 33), thus providing a valid foundation for this investigation. Molecular docking studies suggested that 3 hydrogen bonds were formed between TSN and Glu638 or Ser613 on STAT3 (Figure 2A). To verify the docking results, MST assay was used to determine the direct binding between TSN and STAT3, and a *K*_d_ value of 296 nM was obtained (Figure 2H). Western blot analysis revealed that TSN had no effect on total STAT3 protein levels in HNSCC and CRC cells (Supplemental Figure 1, A–D; supplemental material available online with this article; https://doi.org/10.1172/jci.insight.160606DS1). Next, an appropriate site for tethering TSN to lenalidomide was identified for the design of STAT3 degraders (Figure 2B). Determination of the linkers was geometrically optimized using MOE software. Inspection of the docking simulation of the STAT3/TSN complex revealed solvent-exposed regions suitable for a linker attachment and PROTAC conversion. Therefore, several linkers based on succinic acid and different spacers were constructed from the most active TSN hydroxy moiety by conjugation to the cereblon (CRBN) ligand (lenalidomide) (Figure 2C). According to the above method, a set of degraders with different physicochemical properties (TSM-1 to TSM-6) were designed and synthesized. Details regarding the preparation and structures of the other PROTAC derivatives (TSM-2 to TSM-6) were shown in the Supplemental data (Supplemental Scheme II–V within Supplemental Methods). We then sought to investigate the antitumor efficacy of TSMs in CAL33 cells. Among them, TSM-1 (Figure 2C) containing single azetidine exhibited the best antitumor activity (Supplemental Figure 2). Next, we replaced the E3 ubiquitin ligase of TSM-1 with Von Hippel-Lindau (VHL) and found that the conjugation of VHL with TSN seriously decreased its solubility, resulting in poor efficacy on HNSCC and CRC cells, so TSM-1 was selected for further research. Cellular thermal shift assay (CETSA) and MST methods were used to determine the binding affinity between STAT3 and TSM-1. It was found that TSM-1 directly bound to STAT3 (Figure 2, D–G) with a *K*_d_ value of 308 nM (Figure 2I). Moreover, TSM-1 achieved more pronounced antitumor effects on tumor cells than TSN (Figure 2J).

### TSM-1 inhibited cell viability in multiple HNSCC and CRC cell lines.

**Figure 3:**
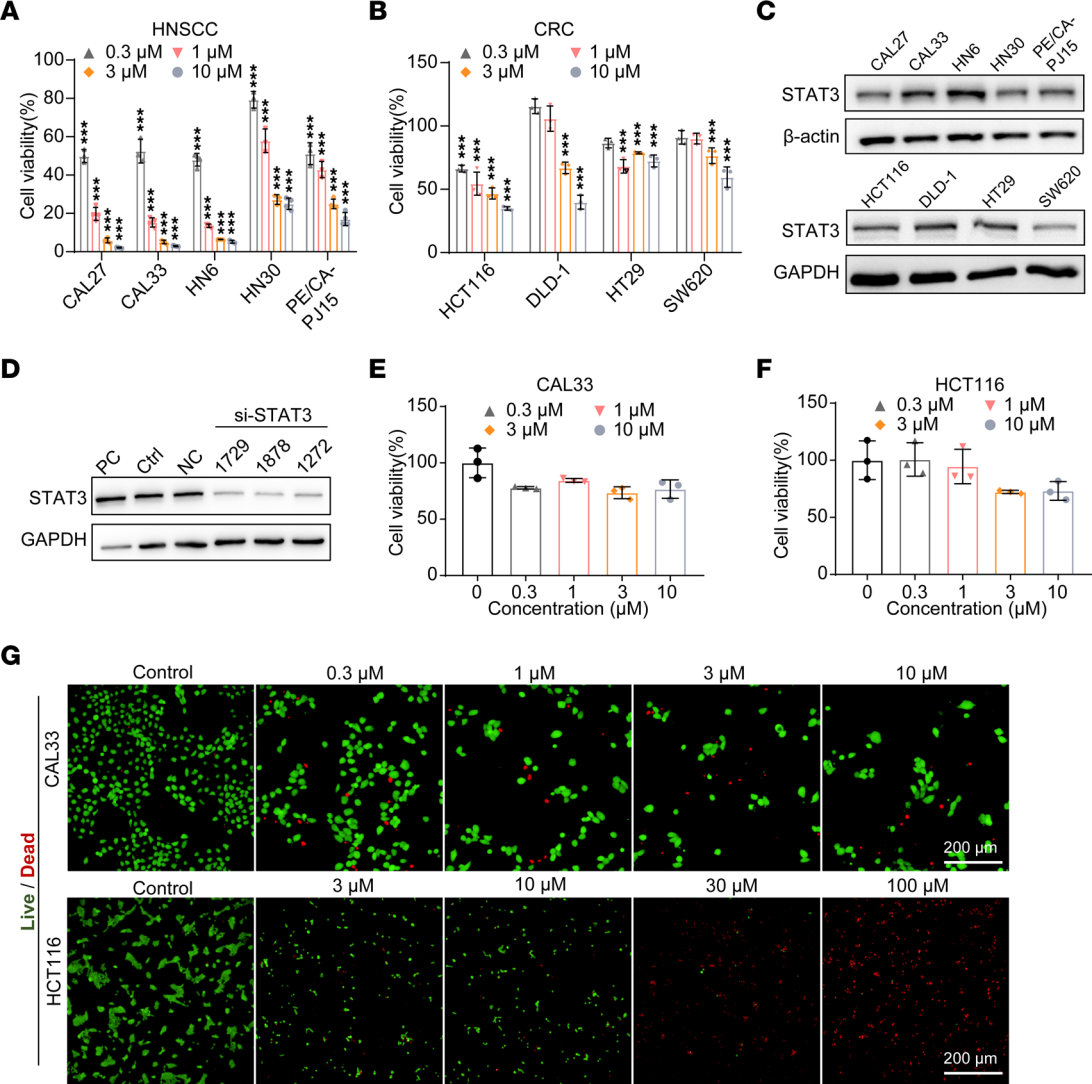
TSM-1 inhibited tumor cell proliferation and viability. (**A** and **B**) HNSCC (**A**) and CRC (**B**) cell proliferation were detected using CCK-8 assays after treatment with TSM-1 for 48 hours (*n* = 3 replicates). (**C**) Protein expression of STAT3 in HNSCC and CRC cells was determined by Western blot. (**D**) STAT3 expression when knocking down of STAT3 through 3 siRNAs (siRNA 1729, 1878, and 1272). PC represented positive control knocking down GAPDH; NC represented negative control. (**E** and **F**) STAT3 knockdown through siRNA 1878 significantly alleviated the inhibition effect of TSM-1 on CAL33 (**E**) and HCT116 cells (**F**) (*n* = 3 replicates). (**G**) CAL33 and HCT116 cells were stained with a live/dead cell viability/cytotoxicity kit after treatment with TSM-1 for 48 hours. Scale bar: 200 μm. (*n* = 3 replicates). The green fluorescence was used to mark the living tumor cells, and the red fluorescence was used to mark the dead tumor cells. ***P < 0.001 when compared with the control group. *P* values are from 2-way ANOVA with Tukey’s multiple-comparison test (**A** and **B**).

The antiproliferative activity of TSM-1 was assessed in a panel of 5 HNSCC and 4 CRC cell lines. The cells were treated with TSM-1 at a range of concentrations for 48 hours, followed by the CCK-8 assay. These results showed that TSM-1 inhibited the viability of HNSCC and CRC cells (Figure 3, A and B), with IC_50_ values that ranged from 0.292 to 5.776 μM (Supplemental Table 1). Moreover, STAT3 protein expression was detected in the same panel of HNSCC and CRC cell lines by Western blot. Specifically, STAT3 protein levels in CAL33 and HN6 cells were higher than the other HNSCC cell lines, which were consistent with their response to the TSM-1 treatment (Figure 3C). Although STAT3 level was also high in HT29 cells, the HT29 cells were not sensitive to TSM-1 at these concentrations. Based on these results and tumor-forming properties, CAL33, HN6, HCT116, and DLD-1 were selected as in vitro models for further studies. To evaluate the effects of TSM-1 on HNSCC and CRC through STAT3, we then knocked down STAT3 using STAT3 siRNA (si-STAT3) (Figure 3D). The cell viability results from STAT3 siRNA alone are shown in Supplemental Figure 3. STAT3 knockdown significantly alleviated the inhibition effect of TSM-1 on CAL33 and HCT116 cells (Figure 3, E and F), indicating the important role of STAT3 in TSM-1 induced antitumor activity. Calcein–acetoxymethyl ester (Calcein-AM) and PI double staining in these cell lines further confirmed the antiproliferative/cytotoxic effects of TSM-1 (Figure 3G and Supplemental Figure 4A), in which the green fluorescence was used to mark the living tumor cells and the red fluorescence was used to mark the dead tumor cells. We found that the number of tumor cells with green fluorescence was significantly reduced, while the number of cells with red fluorescence was also sharply reduced. The adhesion ability of dead CAL33 cells was significantly reduced after TSM-1 treatment, resulting in their shedding in the process of washing steps.

### TSM-1 selectively degraded STAT3 in HNSCC and CRC cell lines.

**Figure 4:**
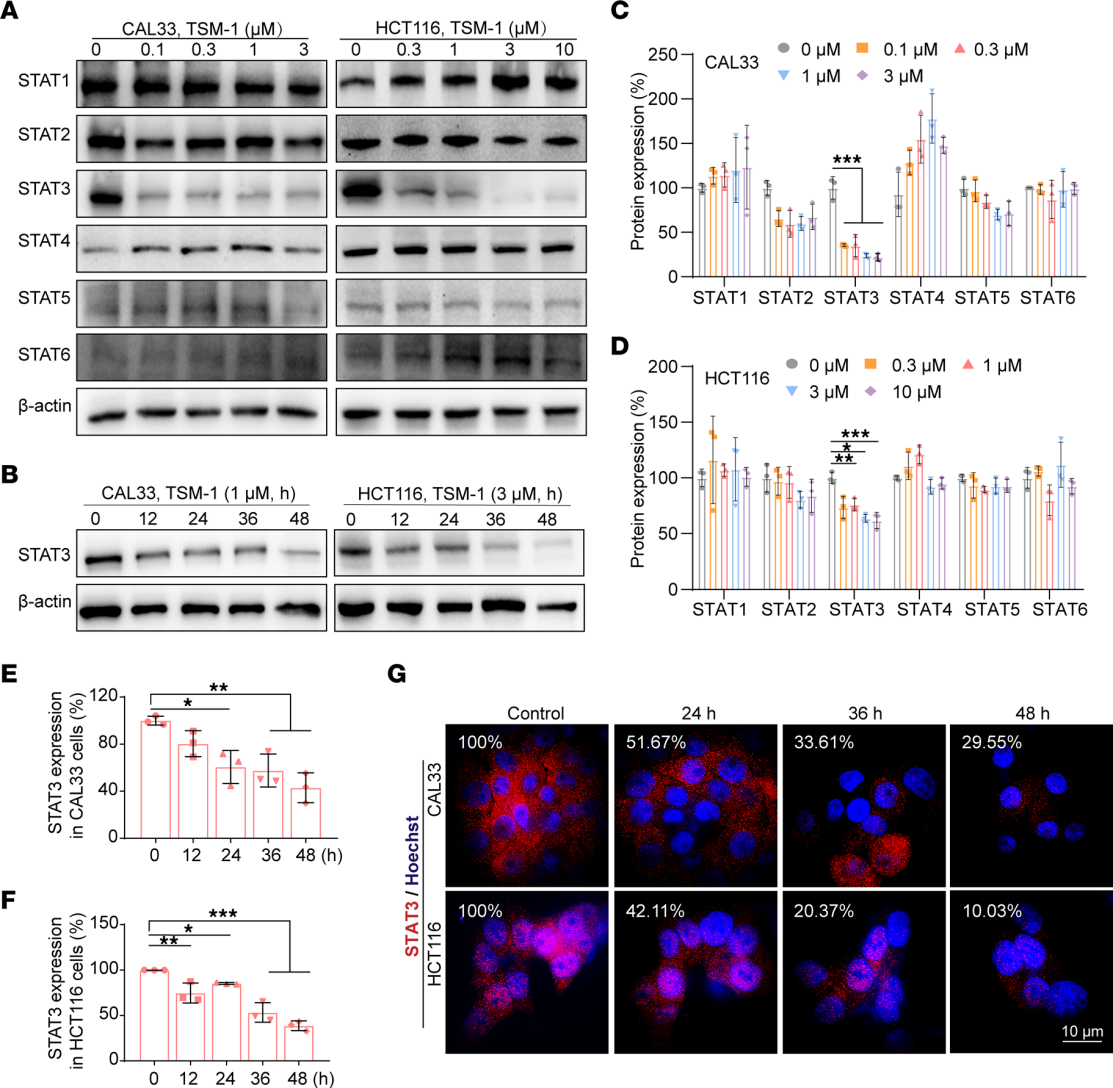
TSM-1 potently and selectively degraded STAT3 in multiple cell-based systems. (**A**) At low concentrations, TSM-1 treatment (36 hours) reduced STAT3 protein expression. (**C** and **D**) Analysis revealed that TSM-1 had minimum effects on other STAT family members (*n* = 3 replicates). (**B**) TSM-1 induced STAT3 degradation in a time-dependent manner in CAL33 and HCT116 cells. (**E**–**G**) Quantitative results of the relative protein levels of STAT3 (**E** and **F**) (*n* = 3 replicates), and the representative microscopic photographs of the CAL33 and HCT116 cells (**G**). Scale bar: 10 μm (*n* = 3 replicates). *P < 0.05, **P < 0.01, and ***P < 0.001 when compared with the control group. *P* values are from 2-way ANOVA with Tukey’s multiple-comparison test (**C** and **D**) or ordinary 1-way ANOVA with Dunnett’s multiple-comparison test (**E** and **F**).

The ability of TSM-1 to induce STAT3 protein degradation was examined in HNSCC and CRC cell lines. Treatment with TSM-1 resulted in the depletion of total STAT3 protein in both HNSCC and CRC cells (Figure 4, A–F, and Supplemental Figure 4, B–D). More importantly, CAL33 and HCT116 exhibited specific STAT3 degradation among the STAT protein family after treatment with TSM-1, suggesting the highly selective nature of TSM-1. An immunofluorescence-based time course study revealed that TSM-1 (3 μM) induced STAT3 protein degradation in a time-dependent manner (Figure 4G and Supplemental Figure 4E), and STAT3 protein levels decreased to 29.55% or 10.03%.

### TSM-1 elicited cell cycle arrest and apoptosis.

**Figure 5:**
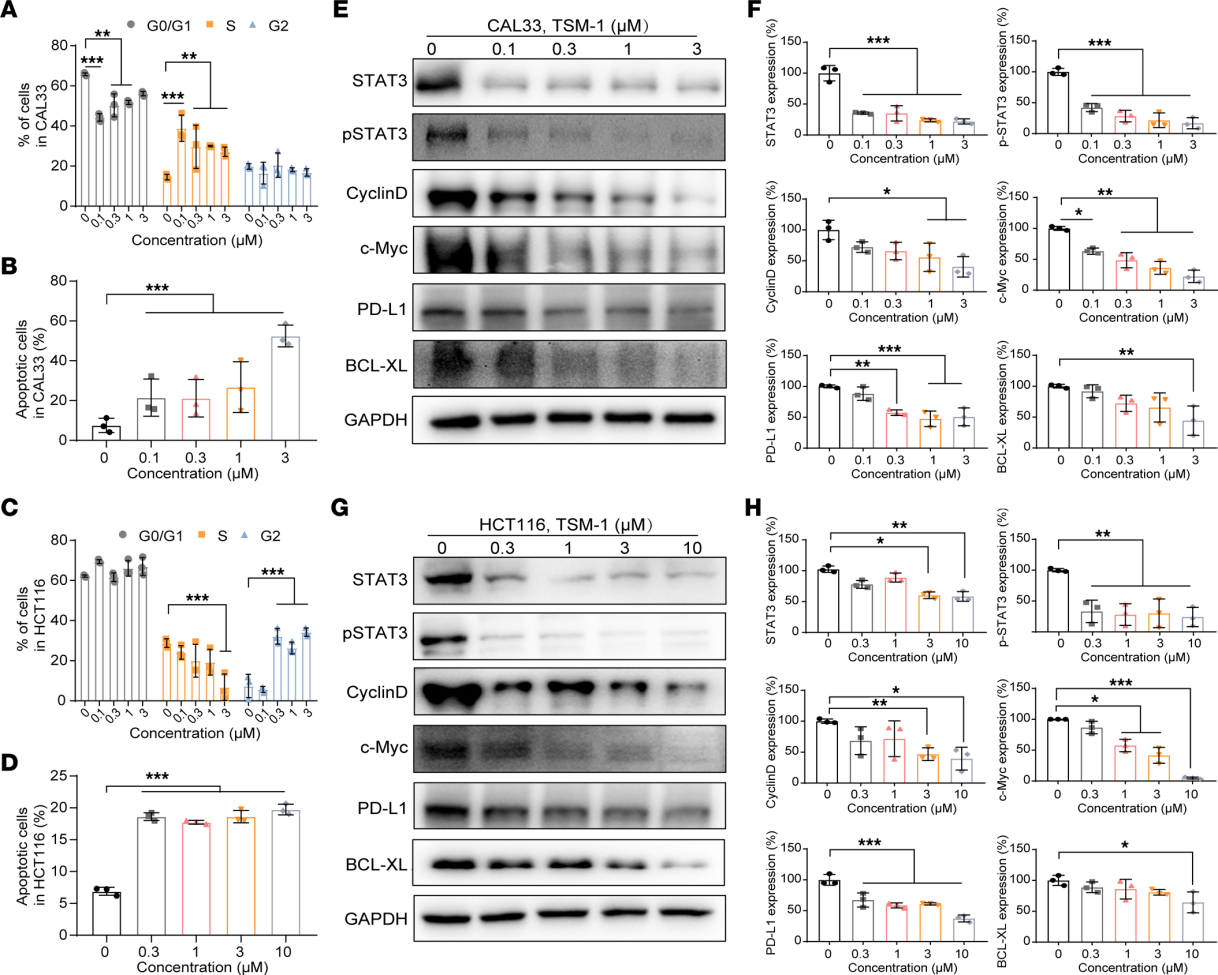
TSM-1 elicited cell cycle arrest and apoptosis. (**A** and **C**) Cell cycle arrest was detected by flow cytometry after treatment with TSM-1 for 36 hours (*n* = 3 replicates). (**B** and **D**) After exposure to TSM-1 for 48 hours, the extent of apoptosis was monitored by flow cytometry (*n* = 3 replicates). (**E**–**H**) TSM-1 treatment (36 hours) reduced levels of STAT3, p-STAT3, cyclin D, c-Myc, PD-L1, and BCL-XL proteins in CAL33 (**E** and **F**) and HCT116 (**G** and **H**) cells (*n* = 3 replicates). *P < 0.05, **P < 0.01, and ***P < 0.001 when compared with the control group. *P* values are from 2-way ANOVA with Tukey’s multiple-comparison test (**A** and **C**) or ordinary 1-way ANOVA with Dunnett’s multiple-comparison test (**B**, **D**, **F**, and **H**).

To investigate the mechanism of action for TSM-1–imposed tumor cell growth inhibition, the effects of TSM-1 on cell cycle and apoptosis were examined using flow cytometry. TSM-1 could significantly induce the increase in the percentage of S phase in CAL33 (Figure 5A and Supplemental Figure 6A), SCC7 (Supplemental Figure 8, F and G), DLD-1 cell lines (Supplemental Figure 5C and Supplemental Figure 7A), and CT26 (Supplemental Figure 8, C and G), accompanied by a decrease in the percentage of G0/G1 phase cells. In addition, TSM-1 caused a significant cycle arrest in G2 phase on HCT116 cells (Figure 5C and Supplemental Figure 7A) and cell cycle arrest in G0/G1 phase on HN6 cells (Supplemental Figure 5A and Supplemental Figure 6A). Furthermore, TSM-1 could trigger apoptosis in CAL33 (Figure 5B and Supplemental Figure 6B), HN6 (Supplemental Figure 5B and Supplemental Figure 6B), HCT116 (Figure 5D and Supplemental Figure 7B), and DLD-1 (Supplemental Figure 5D and Supplemental Figure 7B) cells in a concentration-dependent manner. The apoptosis of CT26 and SCC7 cells were shown in the Supplemental Figure 8, B and E.

To further clarify the downstream signaling pathway, CAL33, HN6, SCC7, HCT116, DLD-1, and CT26 cells were treated with TSM-1, and the cell lysate samples were analyzed by immunoblotting. TSM-1 treatment could effectively deplete STAT3 protein and reduce the level of important downstream proteins, including p-STAT3, cyclinD, c-Myc, PD-L1, and BCL-XL. These results correlated with previously observed efficacy in the cell cycle and apoptosis analyses (Figure 5, E–H; Supplemental Figure 5, E–H; and Supplemental Figure 9, A–D).

### TSM-1 induced ubiquitination and prolonged proteasome-dependent degradation of STAT3 protein.

**Figure 6:**
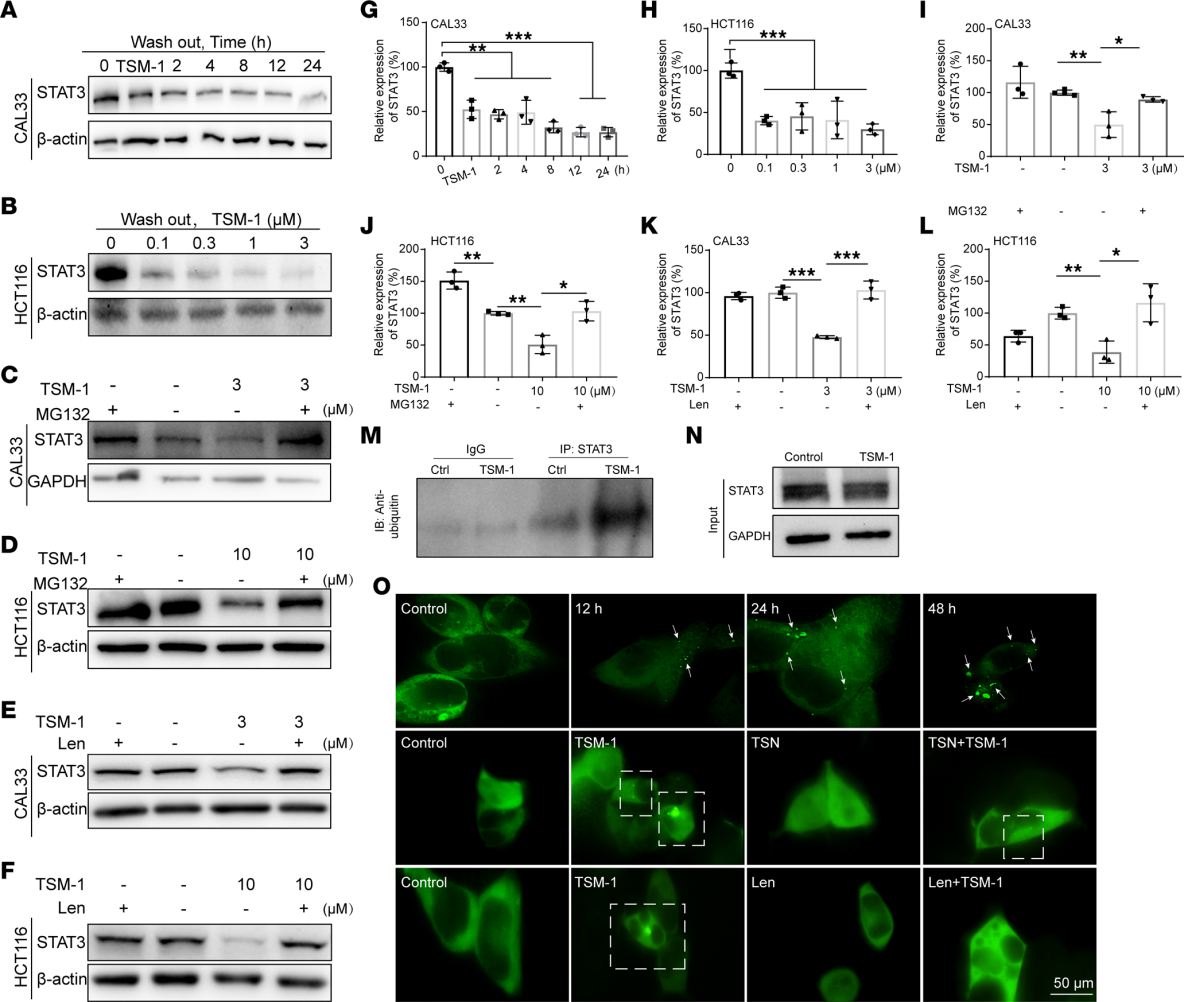
Mechanistic studies for TSM-1 induced STAT3 degradation. (**A**, **B**, **G**, and **H**) TSM-1 treatment (1 μM, 36 hours) reduced STAT3 protein levels when washed out drugs and replaced with fresh medium for different time points in CAL33 cells (**A** and **G**), as well as HCT116 cells (**B** and **H**), when additional incubation with fresh medium for 24 hours (*n* = 3 replicates). (**C**, **D**, **I**, and **J**) Degradation of STAT3 was blocked in CAL33 (**C** and **I**) and HCT116 (**D** and **J**) cells when MG132 (10 μM, 1 hour) was added following 36-hour incubation with TSM-1 (*n* = 3 replicates). (**E**, **F**, **K**, and **L**) Addition of lenalidomide (15 μM) reversed TSM-1 induced downregulation of STAT3 levels in CAL33 (**E** and **K**) and HCT116 (**F** and **L**) cells (*n* = 3 replicates). (**M** and **N**) Immunoprecipitation of ubiquitin when treatment with TSM-1 (1 μM) in 293T cells. IP: STAT3, IB: ubiquitin, and the input samples. (**O**) High-resolution microscopy of GFP-fluorescence images at different time points when treated with 1 μM TSM-1 through GE DeltaVision OMX SR. Formation of ternary complexes in 293T cells after treatment with 1 μM TSM-1 for 36 hours was reversed by pretreatment with 10 μM TSN or 15 μM lenalidomide through High Content Analysis System (*n* = 3 replicates). *P < 0.05, **P < 0.01, and ***P < 0.001 when compared with the control group. *P* values are from ordinary 1-way ANOVA with Dunnett’s multiple-comparison test (**G** and **H**) or 2-tailed unpaired *t* test (**I**–**L**)

An unparalleled feature of PROTAC-based protein degraders is their ability to produce a sustained event-driven degradation of the target proteins in the presence of additional substoichiometric substrate (23). To verify the persistent degradation of TSM-1, STAT3 protein expression was detected at various time points following washout less than 36 hours after TSM-1 treatment in CAL33 cells. The results showed that the decreased STAT3 protein levels remained even after 24 hours of washout (Figure 6, A and G). In HCT116 cells, following TSM-1 treatment at a range of concentrations (24 hours) and an additional 24-hour incubation with fresh medium, the STAT3 protein levels remained significantly reduced (Figure 6, B and H). Taken together, these results confirm the sustained and possibly catalytic effect of TSM-1 on STAT3 degradation.

The heterobifunctional TSM-1 molecule was designed to bind concurrently to STAT3 proteins and CRBN through the TSN and lenalidomide moieties, respectively. This brings the E3 ubiquitin ligase and STAT3 protein into close proximity for subsequent ubiquitination and proteasomal degradation. Therefore, the proteasome inhibitor MG132 and CRBN ligand were used to verify whether TSM-1 degraded STAT3 in a proteasome- and CRBN-dependent manner. In the presence of MG132, the TSM-1–induced STAT3 downregulation in CAL33 (Figure 6, C and I) and HCT116 (Figure 6, D and J) cells was suppressed, supporting proteasome-dependent STAT3 degradation. Moreover, lenalidomide alone had no effect on the levels of STAT3 protein in cells, while preincubation with excess amount of lenalidomide blocked the degradation of STAT3 that was induced by TSM-1 in CAL33 (Figure 6, E and K) and HCT116 (Figure 6, F and L) cells, suggesting that TSM-1 induced STAT3 degradation in a CRBN-dependent manner.

In 293T cells, TSM-1 treatment (1 μM, 36 hours) could reduce the level of STAT3 protein, while increasing the abundance of ubiquitinated STAT3 protein (Figure 6, M and N). The co-IP results suggest that STAT3 protein was ubiquitinated during TSM-1–induced degradation.

### Treatment with TSM-1 induced formation of the ternary complex.

As previously mentioned, the capacity of TSM-1 to degrade STAT3 was highly dependent on the formation of the STAT3–TSM-1–CRBN ubiquitin ligase ternary complex. To real-time visualize TSM-1–induced protein-to-protein interaction, the separation of phases–based protein interaction reporter (SPPIER) technique was applied, which took the advantage of fluorophore phase transition principle and was widely used in PROTAC research (34, 35). Briefly, homogeneous fluorescence could be observed when CRBN-EGFP-HOTag3 or STAT3-EGFP-HOTag6 proteins were expressed in HEK293T cells (Supplemental Figure 10A). Coexpression of these 2 fusion proteins could lead to green fluorescent droplet formation following TSM-1 treatment. The number of fluorescent droplets was positively correlated with the concentration and duration of TSM-1 treatment. Following TSM-1 (3 μM, 1 hour) treatment, fluorescent droplets were formed obviously (Supplemental Figure 10A). High-resolution microscopy (GE DeltaVision OMX SR) was further used to observe the formation of ternary complexes subsequent to TSM-1 (1 μM) treatment at various incubation time points. The results showed that ternary complexes were formed in a time-dependent manner (Figure 6O). When 293T cells were treated with TSN or lenalidomide alone, respectively, there were no green fluorescent drops formed. By contrast, when excess lenalidomide or TSN were combined in advance with E3 ubiquitin ligase or STAT3, fluorescent droplet formation mediated by TSM-1 could be antagonized (Figure 6O). Taken together, these results demonstrate that TSM-1 could form ternary complexes during degradation, which were highly dependent on the E3 ubiquitin ligase and STAT3 ligands, providing further evidence that TSM-1 induced protein-to-protein interactions between STAT3 and CRBN. To facilitate the investigation of the mechanism of action of TSM-1, TSM-Me — an ineffective control of TSM-1 with similar structure — has been synthesized accordingly, in which a methyl group was installed to block its binding with CRBN. Although TSM-Me lost the ability to recruit CRBN, it still exhibited strong affinity with STAT3 (*K*_d_ = 239 nM; Supplemental Figure 11C). However, TSM-Me could not effectively inhibit tumor cell viability and induce STAT3 degradation compared with TSM-1 (Supplemental Figure 11, A, B, D, and E). In addition, the SPPIER technique was used to visualize the interaction of TSM-Me with STAT3 and CRBN; however, the green fluorescent droplet that indicated the formation of STAT3-TSM-Me-CRBN could not be observed following TSM-Me treatment. Taken together, compared with the ineffective control TSM-Me, the small molecule PROTAC (TSM-1) in this research could mediate protein degradation of STAT3 by recruiting E3 ubiquitin ligase CRBN (Supplemental Figure 11, F and G).

### Antitumor efficacy of TSM-1 in cell-derived xenograft models.

**Figure 7:**
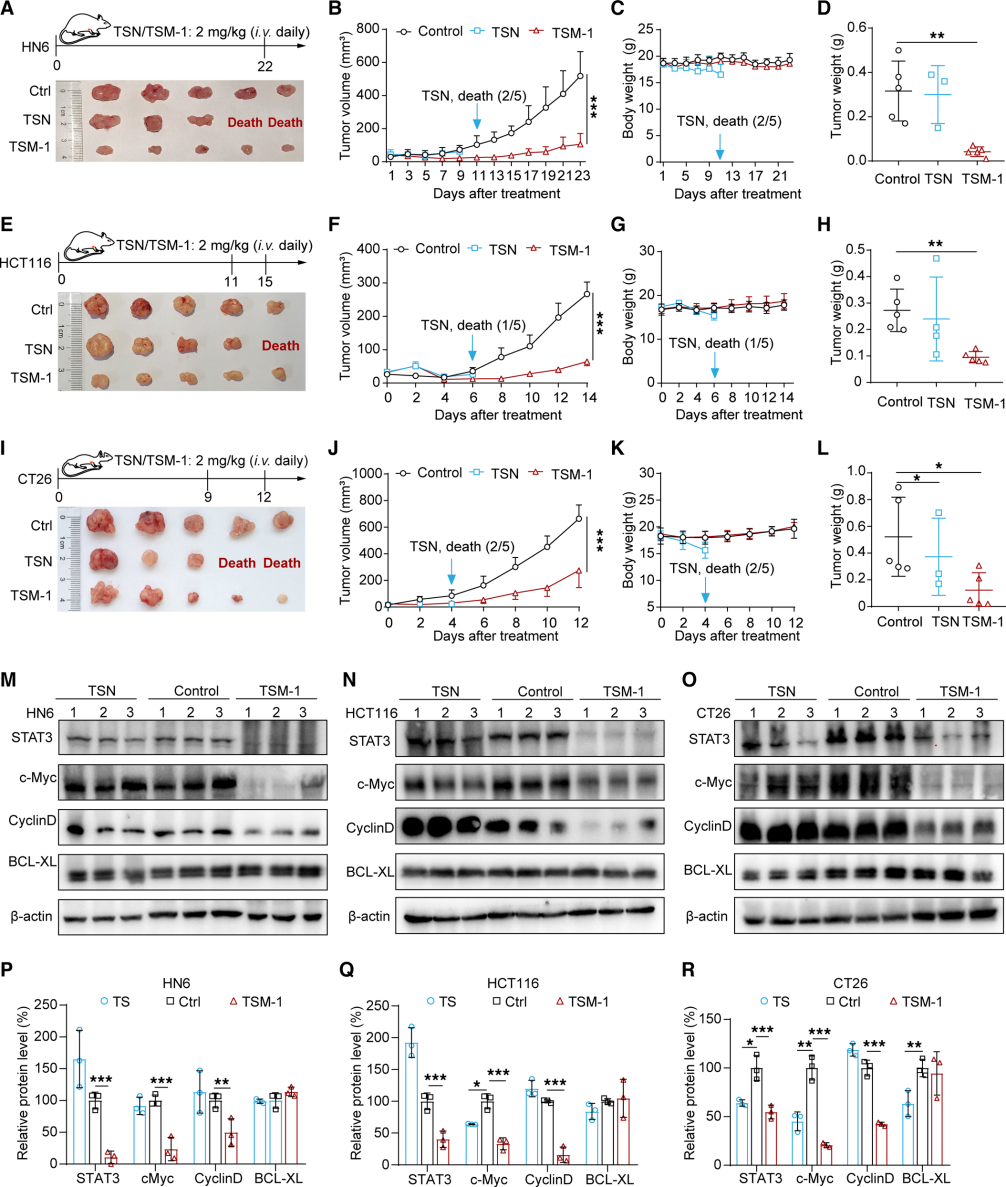
TSM-1 decreased STAT3 protein levels and inhibited tumor growth in vivo. (**A**, **E**, and **I**) The treatment regimen diagrams (*n* = 5 mice). (**B**, **C**, **F**, **G**, **J**, and **K**) Both tumor volume (**B**, **F**, and **J**) and body weight (**C**, **G**, and **K**) were monitored every 2 days. (**A**, **D**, **E**, **H**, **I**, and **L**) When mice were sacrificed, the tumors were photographed (**A**, **E**, and **I**), and tumor weight was recorded (**D**, **H**, and **L**). (**M**–**R**) Western blot analyses show that TSM-1 treatment inhibited STAT3 and its downstream signaling pathway–related target gene expression in vivo. *P < 0.05, **P < 0.01, and ***P < 0.001 when compared with the control group. P values are from ordinary one-way ANOVA with Dunnett’s multiple-comparison test (**D**, **H**, and **L**) or 2-way ANOVA with Tukey’s multiple-comparison test (**B**, **F,** **J**, **P**, **Q**, and **R**).

To evaluate the antitumor effects of TSM-1 in HNSCC and CRC cell–derived xenograft models, HN6 (HNSCC), HCT116 (CRC), and CT26 (CRC) tumor-bearing mice were administered with TSM-1 (2 mg/kg) or TSN (2 mg/kg) daily by tail vein injection. The treatment schedules are shown in Figure 7, A, E, and I. In comparison with the control group, TSM-1 (2 mg/kg) induced significant tumor regression (Figure 7, B, F, and J) and reduced tumor weight (Figure 7, D, H, and L) without affecting the bodyweight of the mice (Figure 7, C, G, and K). In contrast, 2 HN6 tumor-bearing mice (2 of 5), 1 HCT116 tumor-bearing mice (1 of 5), and 2 CT26 tumor-bearing mice (2 of 5) died on the eleventh, sixth, and fourth day following TSN administration, respectively (Figure 7, A, E, and I). Subsequent H&E staining, TUNEL assays, and IHC analyses of tumor sections showed that TSM-1 treatment also led to an increase in necrotic tumor lesions, apoptotic-positive cells, a decrease in the number of proliferating cells, and reduced STAT3 protein expression (Supplemental Figures 12–14), indicating the proapoptosis and antiproliferative effects of TSM-1. To test whether TSM-1 could induce STAT3 protein degradation in vivo, the levels of STAT3 and downstream signaling proteins in the tumor samples were evaluated by Western blot assays. As shown, the levels of STAT3, c-Myc, and cyclin D proteins were lower in the TSM-1 treatment group than the control group (Figure 7, M–R). Taken together, TSM-1 inhibited tumor growth by reducing STAT3 protein levels and suppressing its downstream signaling pathways. To further explore the mechanism of toxicity of TSN, we detected the blood routine in CT26 tumor-bearing models and found that TSN could induce acute inflammatory response, indicating tissue damage. IHC staining of tumor tissues also showed that TSN induced blurred myocardial fibroblasts, severe hyperemia in alveolar cavity, and a reduction of glomerular features (Supplemental Figure 15). In addition, the median survival time of HCT116 tumor-bearing mice in the control group and TSM-1–treated group was 17 days and 35 days, respectively (Supplemental Figure 16, A, C, E, and F). And the median survival time of CT26 tumor-bearing mice in the control group and TSM-1–treated group was 14 days and 22 days (Supplemental Figure 16, B, D, G, and H), respectively, indicating that TSM-1 could greatly inhibit tumor growth and improve overall survival of 2 tumor models. To detect the effects of TSM-1 on T cells in vivo, the expression levels of IL-2, TNF-α, and IFN-γ in tumor tissues have been detected by ELISA kit, and results indicate that TSM-1 could increase these cytokines, showing T cell activation and infiltrates. As expected, the tumors treated with TSM-1 also exhibited increased infiltration of CD8^+^ T cells (Supplemental Figure 17).

**Figure 8:**
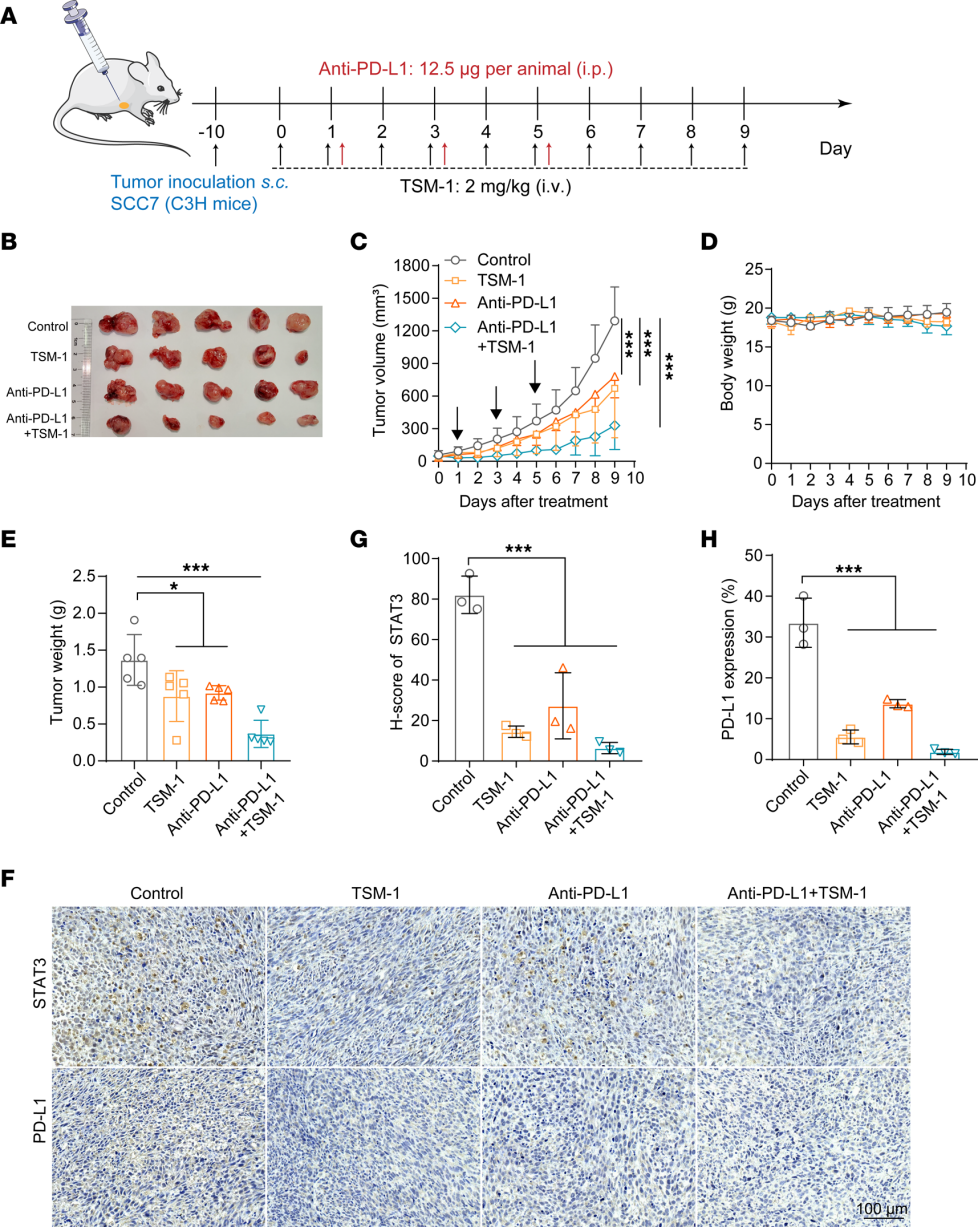
TSM-1 enhanced anti–PD-L1 immune checkpoint blockade. (**A**) The pattern diagram. For the combinatorial immunotherapy, anti–PD-L1 antibody (12.5 μg per animal) was i.p. injected on days 1, 3, and 5 in addition to the vein injection of 2 mg/kg TSM-1. (**B**) When mice were sacrificed, the tumors were photographed (*n* = 5 mice). (**C** and **D**) Both tumor volume (**C**) and body weight (**D**) were monitored every day (*n* = 5 mice). (**E**) When mice were sacrificed, tumor weight was recorded (*n* = 5 mice). (**F**) TSM-1 led to decreased STAT3 and PD-L1 expression compared with control group. (**G** and **H**) Statistical analysis results are shown (*n* = 3 mice). *P < 0.01 and ***P < 0.001 versus the control group. *P* values are from ordinary 1-way ANOVA with Dunnett’s multiple-comparison test (**E**, **G**, and **H**) or 2-way ANOVA with Tukey’s multiple-comparison test (**C**).

On the other hand, STAT3 constitutively occupies the proximal region of the PD-L1 promoter and directly regulates PD-L1 expression (36). STAT3 degradation is expected to inhibit the tumor immune evasion and combine with the anti–PD-L1 antibody for immunotherapy. As previously mentioned, TSM-1 could effectively deplete STAT3 protein and reduce the PD-L1 expression (Figure 5, E and G). Then, we used SCC7–derived (murine HNSCC cell line) xenograft models to validate the potential of combining TSM-1 and anti–PD-L1 antibody. These results proved that TSM-1 plus anti–PD-L1 antibody could induce more significant tumor regression compared with TSM-1 or anti–PD-L1 antibody alone (Figure 8). The engagement of PD-L1 and PD-1 exerts inhibitory effects on T cells by inducing their apoptosis and anergy (36). To investigate whether TSM-1 could reverse the negative influence executed by cancer cells on T cells, we constructed a coculture model of tumor cells with activated Jurkat cells in vitro (37). IL-2, a signature cytokine of activated T cells, was used as a biomarker for monitoring T cell activation and proliferation. The results show that the level of IL-2 in supernatants was significantly increased after treatment with TSM-1 (1 μM). In addition, T cells treated with TSM-1 exhibited better antitumor efficacy against tumor cells in vitro, indicating the improved antitumor effects of activated Jurkat T cells upon TSM-1 treatment (Supplemental Figure 18).

### PDO and PDX models predicted the potential of TSM-1 drug therapy.

**Figure 9:**
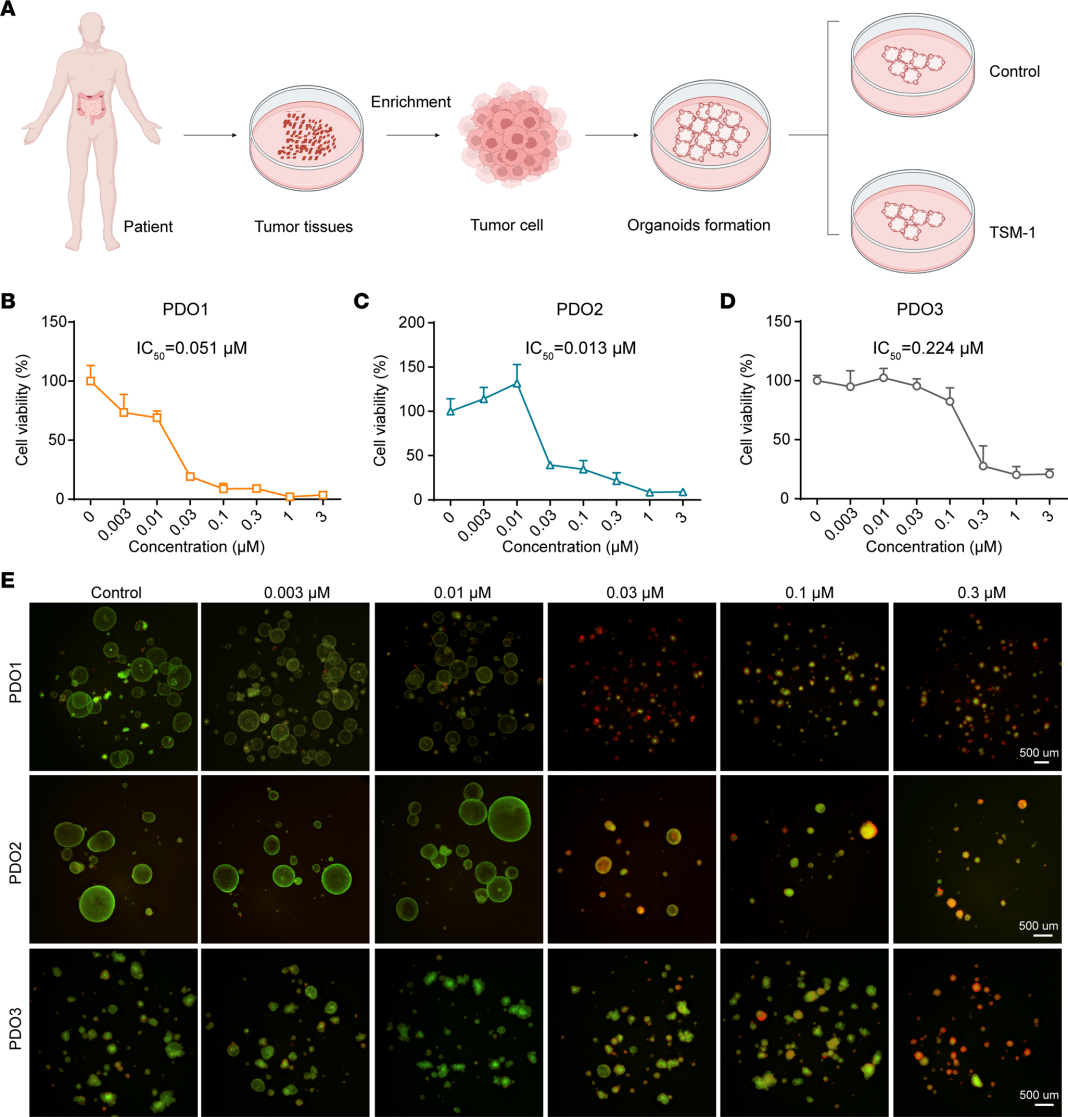
TSM-1 suppressed organoid formation and survival. (**A**) The pattern diagram. (**B**–**D**) Concentration-dependent effects of TSM-1 on organoid survival, based on organoid sizes (%) (*n* = 3 replicates). (**E**) Pictures of patient-derived organoids stained with Live/Dead fluorescent dye after TSM-1 treatment for 6 days (*n* = 3 replicates). Scale bars: 500 μm.

PDO have become an effective clinical-related model for predicting the drug responses, and they retain the morphological and characteristics of human primary tumors (38–40). Prior studies in CRC PDOs have been proved to be suitable for preclinical drug screening and response prediction (41, 42). Therefore, we conducted a prospective and observable assay to validate the efficacy of TSM-1 using CRC PDO models. Three PDOs were successfully employed and treated with various concentrations of TSM-1 for consecutive 6 days. The pattern diagram was shown in Figure 9A. TSM-1 could impede organoid formation and survival after treatment for 6 days (Supplemental Figure 19). Subsequently, LIVE/DEAD staining assay was used to label the live organoids with green, and dead organoids were labeled with red. These results successfully proved that TSM-1 could markedly decrease the number and viability of CRC organoids even at low concentrations (Figure 9E), with the IC_50_ values ranged from 0.013 to 0.224 μM (Figure 9, B–D).

**Figure 10:**
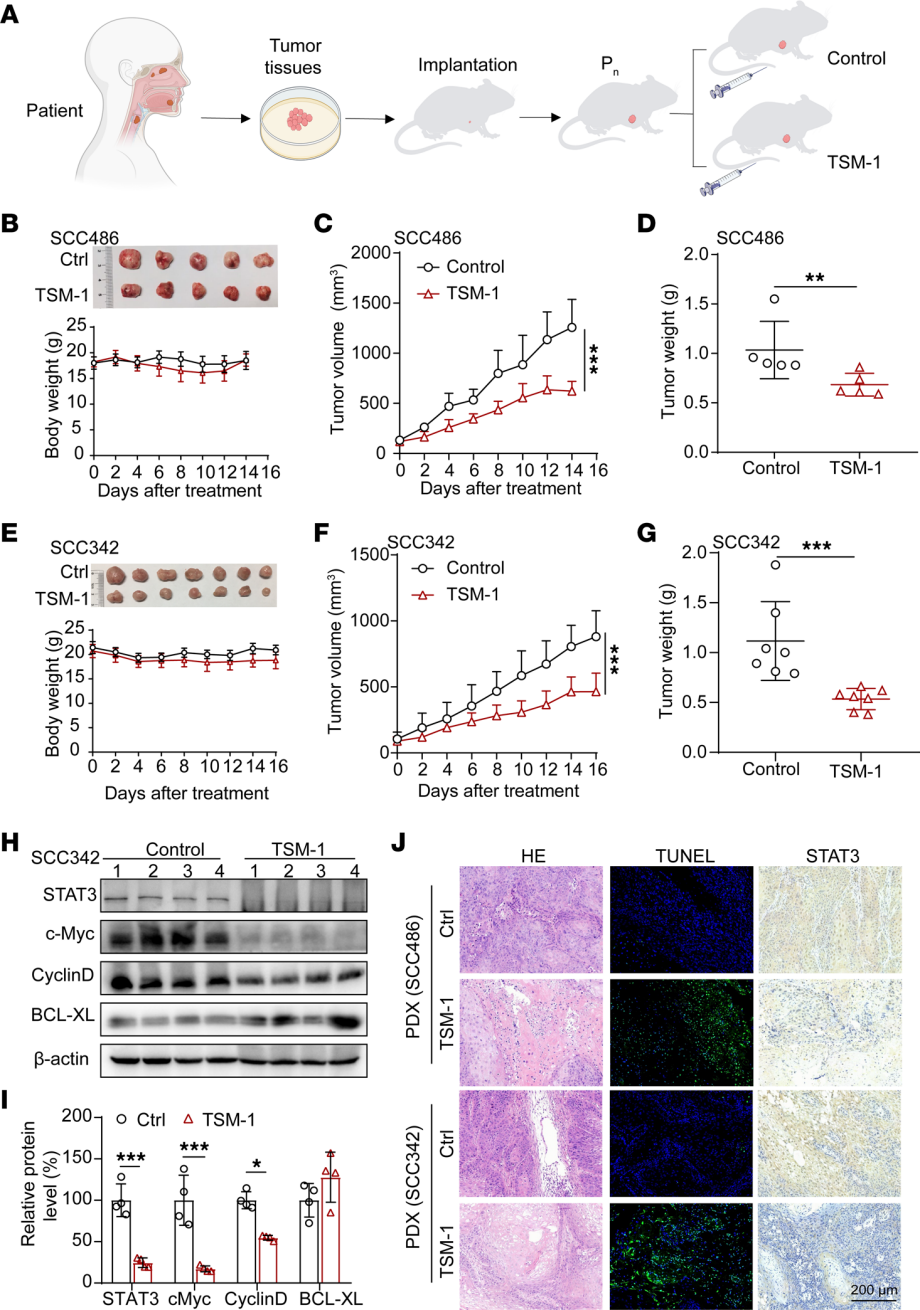
TSM-1 inhibited HNSCC tumor growth in PDX models. (**A**) The treatment regimen diagrams. (**B**–**G**) Both tumor volume (**C** and **F**) and body weight (**B** and **E**) were monitored every 2 days (*n* = 5 mice in **B**, and *n* = 7 mice in **E**); when mice were sacrificed, the tumors were photographed (**B** and **E**), and tumor weight was recorded (**D** and **G**). (**H** and **I**) Western blot analyses showed that TSM-1 treatment inhibited STAT3 and its downstream signaling pathway–related target gene expression in PDX SCC342 model. (**J**) TSM-1 treatment led to significantly increased necrotic lesion, TUNEL^+^ cells, and decreased STAT3 expression in PDX models. *P < 0.05, **P < 0.01, and ***P < 0.001 when compared with the control group. *P* values are from 2-way ANOVA (**C** and **F**), 2-tailed unpaired *t* test (**D** and **G**), or 2-way ANOVA with Tukey’s multiple-comparisons test (**I**).

PDX models that recapitulate the biological property and molecular heterogeneity of human cancers have been considered as an ideal preclinical model for inferring clinical performance (6, 43), thus offering a drug-testing platform that more closely resembles clinical conditions. To assess the clinical value of TSM-1 for STAT3-targeted therapy in patients with HNSCC, we further carried out the efficacy verification in HNSCC PDX models. Our group has successfully established a series of PDX models using human HNSCC specimens (44, 45). Among them, 2 PDX models (SCC486 and SCC342) with endogenously elevated STAT3 mRNA expression were employed to investigate the safety and therapeutic potential of TSM-1 in this study. As the PDX volume reached approximately 100 mm^3^, these HNSCC PDX-bearing mice were randomly divided into 2 groups and administrated i.v. with PBS or TSM-1 (Figure 10A).

Rapid tumor growth rate correlates with poor prognosis in patients with HNSCC (6). The PDX model SCC486 exhibited a high degree of malignancy with rapid tumor growth after engraftment; TSM-1 (5 mg/kg, daily) could inhibit PDX SCC486 tumor growth without obvious toxicity (Figure 10, B–D). The second PDX model SCC342 displayed a slower tumor growth rate, and TSM-1 could successfully hinder the PDX SCC342 tumor progression at 4 mg/kg/day by tail vein injection (Figure 10, E–G). Histopathological examination showed that TSM-1 led to much more necrosis and TUNEL^+^ cells; moreover, such treatment resulted in less STAT3^+^ cells (Figure 10J and Supplemental Figure 20). Similarly, TSM-1 could decrease the protein level of STAT3 and its downstream protein, including c-Myc and cyclin D in PDX tumors (Figure 10, H and I). The H&E staining of tissue samples, including heart, liver, spleen, lung, and kidney, also showed no observable toxicity-related damage subsequent to TSM-1 treatment (Supplemental Figure 21).

All these pioneering efforts supported the potential of TSM-1 as the first PROTAC molecule validated by HNSCC PDX and CRC PDO models, paving the way for future clinical development.

## Discussion

Aberrant STAT3 activation has been implicated in tumor initiation and progression, and preclinical evidence strongly supports STAT3 as a potential treatment target in STAT3-dependent epithelial cancer (1, 3, 46). While there are several reported STAT3 targeting modalities, most of them have significant drawbacks due to the absence of active sites or allosteric regulatory pockets, and the landscape for promising STAT3 inhibitors remains murky (13).

Recently, PROTAC technology has gained momentum as an effective method to induce targeted protein degradation through ubiquitination-mediated proteasomal degradation (47, 48). The application of PROTACs has afforded several advantages for targeting STAT3 over traditional inhibitors (26, 49). First, STAT3 protein degradation can be achieved more efficiently and sustainably than technologies that use STAT3 mRNA with the characteristic of rapid degradation (50, 51). Second, the targeted STAT3 degradation can completely inhibit the transcriptional activity of both dimerized and monomeric STAT3 (19, 21, 26). Third, PROTAC molecules exhibit improved therapeutic efficacy and minimized drug resistance because of the potential for unique catalytic recycling (23, 24).

In 2019, Wang et al. developed SD-36, a degrader that targeted STAT3 in leukemia and lymphoma cells (26). In spite of the demonstrated efficacy of SD-36, the structure-based design of the PROTAC requires a lengthy trial-and-error process. Our research focuses on the highly challenging STAT3-dependent epithelial cancer, including HNSCC and CRC, where STAT3 has emerged as an important drug-discovery target (3). Starting with a STAT3-interacting natural product identified from our compound library by MST assay, this proof-of-concept study is the first to our knowledge to investigate the antitumor potential of the PROTAC strategy in HNSCC and CRC. By taking advantage of their structural diversity and inherent range of biological activity, the semisynthetic design of PROTACs based on natural small molecule shows promise to greatly expand the application of STAT3 degraders in solid tumor.

Recent studies have shown that TSN possesses antitumor effects in various cancers, such as triple-negative breast cancer (TNBC), glioma, ovarian cancer, liver cancer, CRC, by regulating c-Myc, PI3K/AKT/mTOR, and NF-κB, which are strongly correlated with STAT3 (52–55). Zhang et al. also reported that TSN directly bound to the SH2 domain of STAT3 with a *K*_d_ value of 240 nM, leading to the impediment of various oncogenic processes in osteosarcoma (32) and suggesting that TSN is a good natural compound targeting STAT3. This study demonstrated the potential of TSM-1, a TSN-based heterobifunctional PROTAC molecule, to knock down STAT3 protein expression both in vitro and in vivo. Such a strategy offers a potent, reversible, and efficacious approach to degrade STAT3 proteins for malignant epithelial-derived cancer treatment. The bioinformatic-based approach was further employed to analyze STAT3 expression on multiple tumors, indicating gastric cancer (GC), TNBC, and cervical cancer (CC) with high STAT3 expression (Supplemental Figure 22, A–C). Then, we evaluated the antitumor effects of TSM-1 on these tumors, and results indicate that TSM-1 still exhibited good inhibitory effects on those cancer cell lines (Supplemental Figure 22, D–G).

In comparison to the prototype compound TSN used to construct TSM-1, the degrader TSM-1 exhibited enhanced potency and reduced toxicity to inhibit cancer cell proliferation/viability. In xenograft models, TSM-1 inhibited tumor formation without an overt level of toxicity. By contrast, TSN-treated mice displayed limited right hind limb movement, weight loss, and even death. This difference in their toxicity profiles may, in part, result from the potential catalytic activity and recyclability of TSM-1, which reduced the amount of compound required for efficacy and/or improved its absorption and distribution. It is also possible that TSN metabolism may produce toxic metabolites, a concept that warrants further study.

In PDX and PDO models, TSM-1 effectively inhibited the growth of tumors, demonstrating the efficacy of TSM-1 in clinically relevant tumor models, as well as its potential suitability for future translational development. Especially for patients with early stage HNSCC, surgical resection often leads to severe functional impairment and deformed appearance, further affecting overall quality of life. We speculate that the PROTAC-based strategy could be used via intratumorally injection in conjunction with surgery for patients with an early stage HNSCC diagnosis, to improve both treatment outcomes and quality of life. For superficial STAT3-dependent cancers such as HNSCC, intratumoral injection is expected to enhance the efficacy of PROTACs, as well as overcome the potential risk to normal tissues or organs. In addition, overexpression of STAT3 protein is positively associated with advanced-stage HNSCC, especially among patients with stage 4 disease (Supplemental Figure 23). Because there is no specific drug for late-stage HNSCC, the PROTAC-based approach may afford a new treatment option by inducing the total degradation of STAT3 protein, thus inhibiting tumor recurrence and metastasis. Furthermore, systemic administration of TSM-1 resulted in a significant reduction of PD-L1 expression in both HNSCC and CRC tumor tissues (Supplemental Figure 24). The ability of TSM-1 to enhance anti–PD-L1 immune checkpoint blockade was determined. The combination of TSM-1 with anti–PD-L1 antibody significantly enhanced the immunotherapeutic effect, indicating the potential application of the PROATC strategy in immunotherapy, while whether there is a synergistic effect is worth further analysis. It has also been reported that STAT3 controls the malignant behavior of tumor cells, while it dictates the responsiveness to radio- and chemotherapy (56). In addition, STAT3 partially coordinates the cisplatin-resistance phenotype in non–small-cell lung cancer, providing support for the combination of cisplatin, rapamycin, and STAT3 abrogation as a rational therapeutic approach for lung cancer (57). Moreover, X. Zhou proved that the STAT3/HOTAIR/EZH2 axis may serve as a novel therapeutic target for combination therapy of cisplatin and cetuximab to treat patients with HNSCC with PI3K activation (58). Luo et al. also verified that chemo-gene therapy by combining PTX, the first-line chemotherapeutic drug, with STAT3 siRNA may be a practical strategy to effectively suppress tumor growth and metastasis (59). Taken together, TSM-1, the PROTAC targeting STAT3 degradation, is expected to be used in combination with first-line therapeutic agents or targeted drugs in clinic to enhance the antitumor effect as well as overcoming drug resistance.

In summary, we have established proof of concept for the successful application of the PROTAC strategy to target otherwise “undruggable” proteins by rapidly and reversibly degrading endogenous STAT3 protein both in vitro and in vivo for HNSCC and CRC. This PROTAC molecule takes natural small molecules as lead compounds; they provide a powerful tool for oncology research and solid foundation for future drug discovery.

## Methods

Supplemental Methods are available online with this article.

### Compounds and reagents.

TSN was purchased from Pusi Bio-Technology. MG132 (MB5137) and lenalidomide (01131085) were obtained from Meilunbio and Adamas-Beta, respectively. Phytohemagglutinin (PHA, abs47014909), phorbol myristate acetate (PMA, abs9107), and ELISA kits for IL-2 (abs551102) were all obtained from Absin Bioscience Inc. Antibodies for STAT1 (ab109461, 1:1,000), STAT2 (ab32367, 1:1,000), STAT4 (ab68156, 1:1,000), STAT5 (ab194898, 1:1,000), STAT6 (ab32520, 1:1,000), Bcl2 (ab182858, 1:1,000), c-Myc (ab32072, 1:1,000), and ubiquitin (ab134953, 1:500) were obtained from Abcam. Antibodies for STAT3 (9139s, 1:1,000), p-STAT3 (Y705) (9145T, 1:1,000), PD-L1(13684, 1:1,000), cyclin D1 (55506, 1:1,000), BCL-XL (2764, 1:1,000), Bim (2933, 1:1,000), HRP-linked anti–rabbit IgG (7074, 1:5,000), HRP-linked anti–mouse IgG (7076, 1:5,000), and Alexa Fluor 647 conjugate (4418, 1:1,000) were from Cell Signaling Technology. WT mouse IgG (sc-2025, 1:50) was obtained from Santa Cruz Biotechnology Inc. Annexin V-FITC apoptosis assay kit was purchased from Absin, cell cycle and apoptosis analysis kit from was purchased from Beyotime, and Neofect DNA transfection reagent (TF20121201) was provided by Beijing SBS Genetech Co. Ltd. Recombinant human STAT3 protein (P40763) was obtained from Novoprotein.

### Synthesis of TSM-1.

The reagents used in the chemical synthesis were purchased from Tansoole platform. The synthesis route for TSM-1 refers to Figure 2C. A mixture of commercially available S1 (260 mg, 1 mmol) and tert-butyl 3-(bromomethyl) azetidine-1-carboxylate were dissolved in NMP (5 mL). The DIPEA (0.1 mL) was added into the reaction solution and stirred for 12 hours at 80°C. The reaction mixture was then diluted by ethyl acetate (EA) (50 mL) and washed with H_2_O. The organic layer was dried over anhydrous Na_2_SO_4_, concentrated, and purified by column chromatography (20:1 DCM/MeOH) to yield S2 (347 mg, 81%) as white solid powder. ESI-MS (*m/z*) is 427.49 [M+H]^+^.

Trifluoracetic acid (TFA, 1 mL) was added dropwise into a solution of S2 (347 mg) in DCM (10 mL) at 0°C. The mixture was then allowed to room temperature and stirred for 1 hour, and the solvent was removed under vacuo. The residue was suspended in H_2_O, and the saturated NaHCO_3_ was added with efficient stirring at 5°C, the resultant precipitate was filtered off and the filtrate (260 mg, 98%) was concentrated to dryness as white solid powder S3. ESI-MS (*m/z*) is 329.1 [M+H]^+^.

The TSN was dissolved in anhydrous DCM (10 mL); then, the succinic anhydride (500 mg, 5 mmol) and 4-dimethylaminopyridine (244 mg, 2 mmol) were added. The mixture was then allowed to reach room temperature and stirred for 12 hours. Then, the solution was concentrated in vacuo, and the residue was added EA (100 mL). The organic layer was washed with 1N HCl (100 mL) and H_2_O, dried over anhydrous Na_2_SO_4_, and concentrated to yield white solid S4 (640 mg, 95%), which was used without further purification. ESI-MS (*m/z*) is 673.6 [M+H]^+^.

The S3 (329 mg, 1 mmol) and S4 (674 mg, 1 mmol) were dissolved in anhydrous DMF (10 mL), and the solution was cooled to 0°C; then, the HATU (570 mg, 1.5 mmol) and DIPEA (495 μL, 3.0 mmol) were added. The mixture was stirred at 0°C for 12 hours. The residue was diluted with EA and washed with saturated NaCl. Then, the organic phase was dried over Na_2_SO_4_, concentrated, and purified by reverse column chromatography (1 g/L NH_4_HCO_3_/MeCN = 1:1) to yield white solid TSM-1 (493 mg, 50%). ESI-MS (*m/z*) is 985.05 [M+H]^+^ . ^1^H NMR (400 MHz, CDCl_3_) and ^13^C NMR (101 MHz, CDCl_3_) spectras are reported in δ/ppm as follows: ^1^H NMR (400 MHz, CDCl_3_) δ 9.13 (s, 1H), 7.34–7.31 (m, 2H), 7.22–7.19 (m, 1H), 7.11 (s, 1H), 6.75 (d, *J* = 8 Hz, 1H), 6.12 (d, *J* = 1.2 Hz, 1H), 5.74 (s, 1H), 5.26 (s, 1H), 5.22 (s, 1H), 5.13–5.10 (m, 1H), 4.60 (s, 1H), 4.32–4.20 (m, 5H), 4.14–4.06 (m, 1H), 3.92–3.90 (s, 1H), 3.76 (s, 1H), 3.60 (s, 1H), 3.42 (s, 1H), 2.95–2.91 (m, 2H), 2.76–2.68 (m, 6H), 2.36 (m, 2H), 2.22–2.17 (m, 2H), 2.09–2.05 (m, 2H), 2.04–2.03 (m, 2H), 2.00 (s, 2H), 1.96 (s, 3H), 1.93 (s, 1H), 1.90 (s, 2H),1.69 (d, *J* = 9.2 Hz, 1H), 1.56–1.51 (m, 1H), 1.34–1.32 (dd, *J* = 2.6 Hz, *J* = 4.9 Hz,1H), 1.30 (s, 3H), 1.14–1.08 (m, 3H), 0.78 (s, 3H). ^13^C NMR (101 MHz, CDCl_3_) δ 207.17, 172.20, 171.78, 171.44, 170.67, 170.21, 143.02, 142.98, 142.55, 141.50, 140.79, 132.04, 129.94, 126.89, 122.62, 117.42, 116.51, 112.93, 112.74, 112.07, 94.97, 78.74, 83.72, 72.21, 70.16, 70.06, 74.88, 51.97, 48.62, 45.97, 44.06, 42.64, 41.54, 39.40, 39.39, 38.46, 38.43, 35.11, 33.67, 29.14, 28.80, 28.62, 28.06, 23.83, 23.30, 22.43, 21.58, 20.90, 19.28, 19.24, 15.77.

### Cell culture.

Authenticated 293T, HCT116, DLD-1, HT29, and SW620 cell lines were purchased from the Cell Bank of the Shanghai Institute of Cell Biology (SIBS), CAS. CAL27, CAL33, HN6, HN30, and PE-CA/PJ15 were gifts from The Ninth People’s Hospital Affiliated to Shanghai Jiao Tong University School of Medicine. HCT116 and HT29 cells were cultured in McCoy’s 5A medium (16600082, Thermo Fisher Scientific), DLD-1 and SW620 cells were cultured in RPMI-1640 medium (11875101, Thermo Fisher Scientific), and the rest of the cell lines were all cultured in DMEM (MA0212, Meilunbio). Human T leukemia cells (Jurkat T cells) were obtained from Zhong Qiao Xin Zhou Biotechnology Co. Ltd. and were cultured in RPMI-1640 medium. All culture media were supplemented with 10% (v/v) FBS (10091155, Thermo Fisher Scientific) and 1% penicillin/streptomycin (PWL062, Meilunbio), and the cells were grown at 37^o^C under 5% CO_2_/95% air in a humidified incubator.

### Bioinformatic analysis of STAT3 from public databases.

The original RNA-Seq data and clinical information across all available caner types and HNSCC (GSE30784)/CRC (GSE21815) data sets were obtained from TCGA (The Cancer Genome Atlas) platform (https://cancergenome.nih.gov/) (30) and GEO online database (31, 60), respectively. Then, the mRNA expression levels of STAT3 were determined via R software (v3.5.1). Briefly, the differentially expressed genes (DEGs) were identified by limma package. Then, the mRNA expression pattern of these DEGs or STAT3 were visualized with a heatmap, box plot, or violin plot using the pheatmap or ggplot2 package.

### CCK-8 assay.

Cultured HNSCC and CRC cells were seeded into 96-well plates (3599, Corning) at the density of 5 × 10^4^ cells/well in a volume of 100 μL. After an overnight incubation, the cells were treated with TSM-1 at a range of concentrations for 48 hours. CCK8 solution (10 μL/well) was added, and the incubation continued for 1 hour. Then, the OD values (450 nm) were measured using Cytation 5 microplate reader (BioTek). The data normalized to the untreated control and presented as “% Inhibition” using the following formula: (OD_A_ – OD_B_)/(OD_C_ – OD_B_) 100%, which was used to determine IC_50_ values. OD_A_ represents groups with different concentrations of TSM-1, OD_B_ represents blank groups with only medium, and OD_C_ represents control groups.

### Coculture experiments and IL-2 detection.

HCT116 or CAL33 cells were seeded into a 12-well plate at the density of 1 × 10^4^ cells/well for attachment, and they were subsequently treated with TSM-1 (1 μM) for 24 hours. Then, Jurkat T cells (6 × 10^4^) were prestimulated using PHA (10 μg/mL) and PMA (10 ng/mL) for 4 hours. HCT116 or CALL33 cells were cocultured with activated Jurkat T cells for another 24 hours. ELISA kits were used to detect the level of IL-2, a biomarker for monitoring T cell activation, in the supernatant medium. The underlying living cancer cells were virtualized with crystal violet staining. After being dissolved in 10% acetic acid, the cells were quantified using Cytation 5 microplate reader (BioTek) at 595 nm.

### si-STAT3.

siRNA, negative control (NC), and positive control (PC) for GAPDH oligonucleotide sequences were synthesized by GenePharma. The sequences were shown in Supplemental Table 2. Transfection was performed using TransMate (GenePharma) according to the protocol. Cultured CAL33 or HCT116 cells (3 × 10^5^ cells/well) were seeded in 6-well plates and were transfected with siRNA. After 48 hours of transfection, cells were collected, and STAT3 protein expression was detected by Western blot.

### Calcein-AM/PI double staining.

To further evaluate cell growth of HNSCC and CRC, Calcein-AM/PI double staining kit (Meilun, MA0361) was used according to the manufacturer’s instructions. In brief, HNSCC or CRC cells (8 × 10^4^ cells/well) in 96-well plates were incubated with TSM-1 for 48 hours. Then, cells were labeled with calcien-AM (2 μM) plus PI (8 μM) for 15 minutes, followed by fluorescence microscopy to observe the fluorescence images by detecting red (Calcein-AM, Ex/Em = 495/515 nm, AM) and green (Ex/Em = 535/617 nm, propidium iodide [PI]) signals on SparkCyto.

### Cell cycle analysis.

For cell cycle analysis, HNSCC or CRC cells (3 × 10^5^ cells/well) were seeded into 6-well plates (3516, Corning) and treated with various concentration of TSM-1. The cells were then washed twice with ice-cold 1× PBS. After digestion, cell mass precipitation was obtained and fixed in 50% ethanol overnight at 4°C. Finally, the cells were stained with PI plus RNase A (PI: RNase A = 1:9) for 1 h at room temperature and analyzed by flow cytometry.

### Annexin V apoptosis assay.

For cell apoptosis detection, Annexin V–FITC apoptosis assay kit (MA0220, Meilunbio) was used. Briefly, HNSCC or CRC cells (3 × 10^5^ cells/well) were seeded into 6-well plates (3516, Corning) and treated with various concentrations of TSM-1. When washed with ice-cold 1× PBS, cell mass precipitation was obtained and fixed in binding buffer and 5 μL Annexin V–FITC for 15 minutes at room temperature. Then, the PI regent (5 μL) was added and the cells were analyzed by flow cytometry.

### Co-IP.

The HEK 293T cells (1.5 × 10^5^ cells/well) were grown in 6-well plates and were transfected with STAT3 plasmid DNA (0.2 μg/well) when cultured to approximately 50% confluence. After 24 hours, the cells were treated with TSM-1 (3 μM) for another 36 hours. The cells were collected, washed with PBS, and lysed in cell lysis buffer (P0013F, Beyotime). For IP assays, 2 mg of total protein lysate was used. Added for 5 μL anti-STAT3 (1 μg/μL) or equivalent volume normal rat IgG antibody (0.4 μg/μL), total protein was incubated overnight at 4°C. After washing, 20 μL protein A beads (Santa Cruz Biotechnology Inc., sc-2003) were added into the above immune complexes and then incubated at room temperature for 1 hour to form bead conjugates. Then, the bead conjugates were collected and washed 5 times with 200 μL of precooled IP lysis (1861603, Thermo Fisher Scientific). Finally, the samples were incubated with 5SDS loading buffer for immunoblotting.

### Immunoblotting.

For immunoblotting, HNSCC or CRC cells (3 × 10^5^ cells/well) were grown in 6-well plates and exposed to TSM-1 at specified concentrations. Cells were lysed in ice-cold RIPA buffer (sc-24948, Santa Cruz Biotechnology Inc.) containing 50 protease inhibitors (catalog 11697498001). Total proteins were separated by 10% (PG112) or 12.5% (PG113) SDS-PAGE gel (EpiZyme) and transferred to a 0.2 μm PVDF membrane (10600021, Cytiva). PVDF membranes were blocked in 1× TBST (abs952, Absin) with 5% nonfat milk for 1 hour at room temperature. Signals were visualized by Odyssey Infrared Imaging System (Tanon) and analyzed by using ImageJ software (NIH). β-Actin (ab6276, 1:1000) or GAPDH (ab8245, 1:1000) was used as control.

### Immunofluorescence.

The CRC or HNSCC cells (5 × 10^4^ cells/well) in a 20 mm confocal dish were treated with DMSO (solvent control) or TSM-1 for different hours, fixed with 4% paraformaldehyde for 20 minutes, and permeabilized with 0.5% Triton X-100 (diluted with water) for 5 minutes. When blocking with 1% BSA (MA0100, Meilunbio) in 1× PBS (MA0015, Meilunbio) for 1 hour at room temperature, cells were stained with anti-STAT3 antibody (1:1000, Cell Signaling Technology [CST]) at 4°C overnight. Following incubation, cells were washed with ice-cold 1× PBS and incubated for another 60 minutes with fluorescently labeled secondary antibody (1:500). The cells were washed with 1× PBS and stained with Hoechst 33342 for 10 minutes. Fluorescence images were visualized using GE DeltaVision OMX SR.

### SPPIER.

Plasmids were showed in Supplemental Table 3. HEK 293T cells were grown in 96-well glass-bottom plates (J04961, Jingan) at the density of 1 × 10^4^ cells/well. When cells were cultured to approximately 50% confluence, the transfection was performed with 0.2 μg of each plasmid DNA through Neofect DNA transfection reagent (TF20121201, Beijing Genomtech). Twelve hours after transient transfection, real-time imaging was performed with an incubation chamber, which was maintained at 37°C and 5% CO_2_. The fluorescence images were captured by GE DeltaVision OMX SR and High Content Analysis System (PerkinElmer).

### Molecular docking.

The cocrystal structure of STAT3 (Protein Data Bank [PDB], 1BG1) was used to model the binding poses of TSN with STAT3/DNA complex. In addition, the crystal structure of DDB1-CRBN E3 ubiquitin ligase bound to lenalidomide (PDB, 4CI2) was used to generate the structure between CRBN and lenalidomide in Figure 2G using PyMOL software.

### Microscale thermophoresis (MST) assay.

The MST assay was performed using Monolith NT.115 (NanoTemper). First, STAT3 protein was dissolved in distilled water at 20 μM. For optimal binding, the assay was performed in a buffer containing 10 mM CaCl_2_, 50 mM NaCl, 50 mM HEPES (pH 7.5), and 5 mM DTT. Then, protein was labeled with fluorescent dye and incubated with different concentrations of TSM-1 or TSM-Me. Incubated at room temperature away from light for 10 minutes, the mixture was loaded into the capillary and scanned by Monolith NT.115.

### CETSA.

CAL33 or HCT116 cells were grown in 6-well plates at the density of 3 × 10^5^ cells/well and incubated with TSM-1 (80 μM) for 3 hours at 37°C; they were then lysed using NP-40 lysis buffer containing 20× phosphatase (04906837001, Roche) and 50× complete protease inhibitors. The proteins were subsequently divided into 7 or 8 equal aliquots, followed by cooling for 3 minutes at different temperatures. Finally, all the samples were incubated with 5× SDS loading buffer for immunoblotting.

### Syngeneic and xenograft tumor models.

Four-week-old female BALB/C mice, BALB/C nude mice, and C3H/HeNCrl mice were all purchased from Shanghai Model Organisms Center Inc. and were reared adaptively in a sterile environment for 1 week prior to experiments. For the syngeneic (3 × 10^5^ CT26 cells and 8 × 10^5^ SCC7 cells per mouse) and xenograft tumor models (5 × 10^6^ HCT116 cells and 1 × 10^7^ HN6 cells per mouse, respectively), cells resuspended in 100 μL ice-cold 1× PBS were s.c. injected into the right flanks of mice/nude mice. When the tumor volume reached about 50 mm^3^, the mice/nude mice were randomized into separate groups according to the design (*n* = 5 per group): vehicle control, TSN, TSM-1, anti–PD-L1 antibody alone, and anti–PD-L1 antibody plus TSM-1. TSN (2 mg/kg), TSM-1 (2 mg/kg), and anti–PD-L1 antibody (12.5 μg/animal) were given in all cases involved. The TSN and TSM-1 were dissolved in 5% DMSO:10% PEG 400:5% Tween 80:80% normal saline and were administered via tail vein every day at the specified dose and with the duration indicated. To evaluate the effect of TSM-1 on the overall survival of mice, the tumor growth and survival of tumor-bearing mice were continually monitored after therapy, as suggested. Tumor volumes were measured daily during the experiment, and the experimental end point was determined when the tumor volume reached 2,000 mm^3^; then, the overall survival of mice was plotted using GraphPad Prism 8.0. Anti–PD-L1 antibody was dissolved in normal saline and administered via i.p. injection on days 1, 3, and 5 after treatment. Tumor volumes and animal weights were measured every day. Tumor volumes were calculated using the following formula: (length × width^2^)/2. When the experiments were terminated, the mice were sacrificed and tumors were harvested for further analyses (e.g., H&E staining, TUNEL, Ki67, and STAT3 protein detection) by Servicebio. For CT26 tumor-bearing mice, plasma was separated and centrifuged at 3,000*g* in 4°C for 15 minutes; then, a routine blood test was performed in an automatic blood cell analyzer (BC-2800vet).

### PDX model of HNSCC.

All patient-derived tumor samples were obtained from Shanghai Ninth People’s Hospital with each patient’s written informed consent and research ethics board approval in accordance with the Declaration of Helsinki. PDX models were generated as previously described (44). Briefly, tumor pieces from PDX_1_ (SCC486) and PDX_2_ (SCC342) were engrafted to 5-week-old female nude mice (Shanghai Super-B&K Laboratory Animal Corp. Ltd.). In this study, the fourth generation (SCC486) and sixth generation (SCC342) were expanded for drug treatment. Once the tumor volumes reached about 100 mm^3^, TSM-1 (diluted into 200 μL solution containing 5% DMSO, 10% PEG 400, 5% Tween 80, and 80% normal saline) was administered daily to tumor-bearing nude mice via tail vein injection. Tumor volume and animal weights were measured every 2 days. Tumor volume calculation, sample harvest, and analyses were the same as those described in the previous section.

### PDO models of CRC.

Organoids were established as described previously by D1Med Technology Inc. (61, 62). In this study, 50 organoids/well were laid into 48-well plates and incubated in 5% CO_2_/95% air at 37°C for total 6 days. The organoids were stained with fluorescence and photographed under microscope (Zeiss, Vert A1), and the area of live organoids was counted. On day 3, freshly prepared drug and medium mixture were added. At the end of the sixth day, live/dead organoids were stained with fluorescence, and the area of live organoids (shown in green fluorescence) was photographed. The organoid survival rate was calculated by recording organoid size (%) at specified concentrations. The formula is as follows: organoid survival rate = (organoids alive on day 6 in TSM-1 group/organoid alive on day 0 in TSM-1 group)/(organoids alive on day 6 in control group/organoids alive on day 0 in control group) × 100%.

### Statistics.

Statistical analyses were performed using 2-tailed unpaired *t* test or 1-way ANOVA with Dunnett’s multiple comparisons test (GraphPad Prism 8.0.1 software), 2-way ANOVA, and Tukey’s test were used for analyses among multiple groups. And the H-score to quantify STAT3 protein levels in tumor tissues has been performed using ImageJ software (NIH). Significance is defined as *P* < 0.05. Data are presented as mean + SD (bars) or mean ± SD (curves) from at least 3 independent experiments.

### Study approval.

All animals in the syngeneic and xenograft tumor models were treated according to guidelines and approved by the Ethical Committee of Shanghai University of Traditional Chinese Medicine (no. PZSHUTCM220627037). All PDX tumor models were conducted according to the guidelines of the IACUC of Shanghai Jiao Tong University School of Medicine.

## Author contributions

JJ, Yaping Wu, and Z Zhao contributed to data curation, formal analysis, investigation, methodology, writing of the original draft, and review and editing of the manuscript. Ye Wu, QS, and GY contributed to data curation, methodology, and software. YZ, JL, DGN, SL, and JQ contributed to data curation and manuscript editing and revision. Z Zhang and HC contributed to data curation, funding acquisition, and editing. XL, SS, and WZ contributed to conceptualization, resources, supervision, funding acquisition, and review and editing of the manuscript.

## Supplementary Material

Supplemental data

## Version 1 11/22/2022

Electronic publication
