# Supplementary material for: Small-molecule PROTAC mediates targeted protein degradation to treat STAT3-dependent epithelial cancer
Source: JCI Insight. 2022 Nov 22;7(22):e160606. doi: 10.1172/jci.insight.160606 (PMC9746828; doi:10.1172/jci.insight.160606)
Supplement: Supplemental data [file jciinsight-7-160606-s208.pdf]

Supporting information

Small Molecule PROTAC-Mediated Targeted Protein Degradation to Treat STAT3-Dependent Epithelial Cancer

Jinmei Jin[1#], Yaping Wu[2#], Zeng Zhao[3,4#], Ye Wu[1], Yudong Zhou[5], Sanhong Liu[1], Qingyan Sun[4], Guizhu Yang[2], Dale G. Nagle[5,6], Jiangjiang Qin[7], Zhiyuan Zhang[2], Hongzhuan Chen[1], Weidong Zhang[1✉], Shuyang Sun[2✉], and Xin Luan[1✉]

1. Shanghai Frontiers Science Center for Chinese Medicine Chemical Biology, Institute of Interdisciplinary Integrative Medicine Research, Shanghai University of Traditional Chinese Medicine, Shanghai, 201203, China
2. Department of Oral and Maxillofacial-Head Neck Oncology, Shanghai Ninth People's Hospital, Shanghai Jiao Tong University School of Medicine; College of Stomatology, Shanghai Jiao Tong University; National Center for Stomatology; National Clinical Research Center for Oral Diseases; Shanghai Key Laboratory of Stomatology, Shanghai, 200011, China
3. School of Pharmacy, Shanghai Jiao Tong University, Shanghai, 200011, China
4. China Institute of Pharmaceutical Industry, Shanghai, 201203, China
5. Department of Chemistry and Biochemistry, College of Liberal Arts, University of Mississippi University, MS, 38677-1848, USA
6. Department of BioMolecular Sciences and Research Institute of Pharmaceutical Sciences, School of Pharmacy, University of Mississippi, MS 38677-1848, USA
7. Zhejiang Provincial Research Center for Upper Gastrointestinal Tract Cancer, The Cancer Hospital of the University of Chinese Academy of Sciences (Zhejiang Cancer Hospital), Hangzhou, 310022, Zhejiang, China.

# These authors contributed equally to this work.

✉ Corresponding authors: Prof. **Weidong Zhang**, Shanghai University of Traditional Chinese Medicine, Shanghai, 201203, China; orcid.org/0000-0002-7384-2490; Email: wdzhangy@hotmail.com.
Prof. **Shuyang Sun**, Shanghai Ninth People's Hospital, Shanghai Jiao Tong University School of Medicine, Shanghai, 200011, China; orcid.org/0000-0002-1342-8941; Email: sunshuyang@sjtu.edu.cn.
Prof. **Xin Luan**, Shanghai University of Traditional Chinese Medicine, Shanghai, 201203, China; orcid.org/0000-0003-3674-256X; Email: luanxin@shutcm.edu.cn.

Synthesis of TSMs.

Scheme II: Synthetic route of preparing TSM-2.

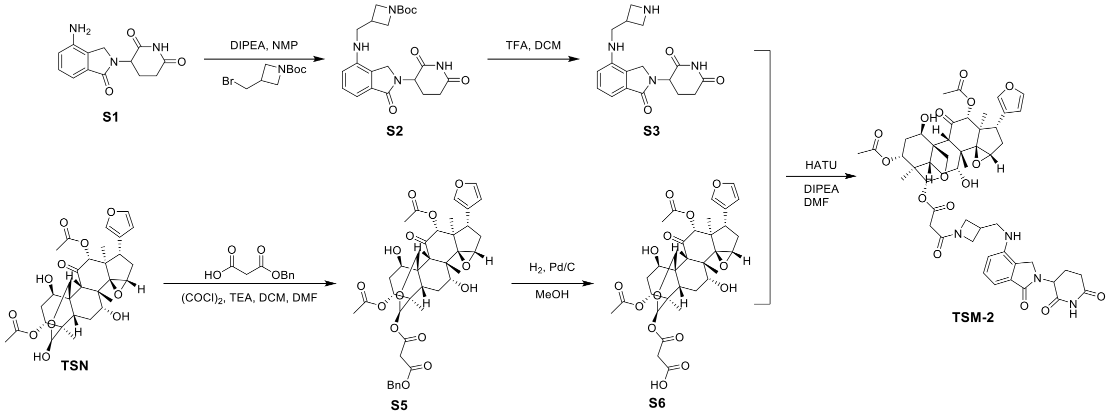

3-(benzyloxy)-3-oxypropionic acid (233 mg, 1.2 mmol) was dissolved in anhydraous DCM (5 mL). Oxaloyl chloride (0.12 mL, 1.4 mmol) and the catalytic amount of anhydrous DMF were added into the reaction solution at 0°C. The mixture was then stirred at room temperature until the reaction of raw materials was complete. When the solvent was evaporated, the crude was dissolved in 2 mL anhydrous DCM.

A mixture of **TSN** (574 mg, 1 mmol) and triethylamine (0.27 mL, 2 mmol) were dissolved in anhydrous DCM (5 mL), which was allowed to room temperature and stirred for 12 h. The reaction mixture was diluted with DCM and washed with water for three times. The organic layer was washed with saturated NaCl, dried over Na_2SO_4, concentrated and purified by column chromatography (1:2 PE/EA) to yield white solid product **S5** (390 mg, 52%). ESI-MS: *m/z* 751.8 $[M+H]^+$.

The above **S5** was dissolved in methanol (10 mL) and 10% palladium carbon (100 mg). After being replaced with hydrogen for three times, the mixture was stirred at room temperature until the reaction was complete. The crude product **S6** (310 mg, 90%) was obtained by adding diatomite for extraction and filtration, followed by washing with methanol for three times. ESI-MS: *m/z* 660.7 $[M+H]^+$.

TSM-2 (460 mg, 47%, ESI-MS: *m/z* 969.88 $[M+H]^+$) was prepared following the procedure described for **TSM-1**.

Scheme III: Synthetic route of preparing TSM-3.

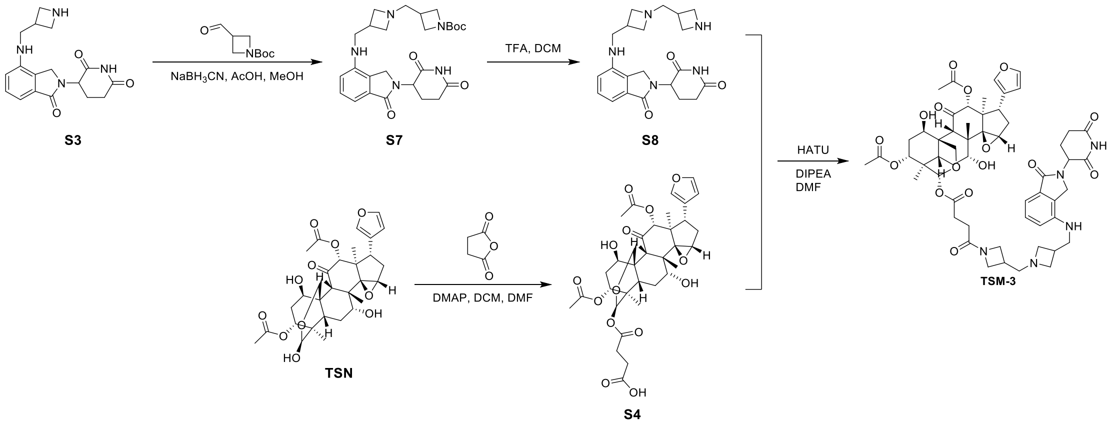

The mixture of **S3** (328 mg, 1 mmol) and 3-formylazacyclobutane-1-carboxylate tert-butyl ester (370 mg, 2 mmol) were dissolved in 10 mL methanol. Then the sodium cyanoborohydride (315 mg, 5 mmol) and 0.1 mL acetic acid were added into the reaction solution and stirred at room temperature until the reaction was complete, followed by evaporating the solvent and being re-dissolved in ethyl acetate. The organic layer was washed with H_2O and dried over anhydrous Na_2SO_4, concentrated, and purified by column chromatography (20:1 DCM/MeOH) to yield white solid product **S7** (350 mg, 70%), ESI-MS: *m/z* 498.6 $[M+H]^+$.

S7 was dissolved in 10 mL DCM and dropped into 1 mL TFA, then the mixture was stirred at room temperature until the reaction was complete. When the solvent was evaporated, the crude product was re-dissolved in water and dropped into saturated $NaHCO_3$ solution. The products were precipitated at 5°C and filtered to obtain white solid **S8** (266 mg, 95%), ESI-MS: *m/z* 398.5 $[M+H]^+$.

TSM-3 was prepared following the procedure described for **TSM-1** as white solid product (570 mg, 54%), ESI-MS: *m/z* 1055.03 $[M+H]^+$。

Scheme IV: Synthetic route of preparing TSM-4, TSM-5.

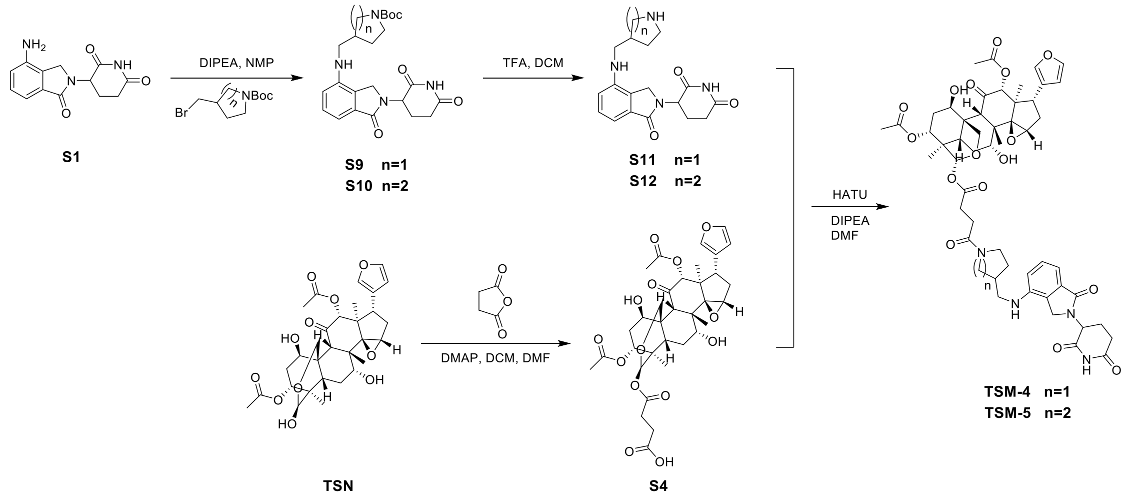

A mixture of commercially available **S1** (260 mg, 1 mmol) and tert-Butyl 3-(bromomethyl) azetidine-1-carboxylate were dissolved in NMP (5 mL). The DIPEA (0.1 mL) was added into the reaction solution and stirred for 12 h at 80 ^oC. The reaction mixture was then diluted by EA (50 mL) and washed with H_2O. The organic layer was dried over anhydrous Na_2SO_4, concentrated, and purified by column chromatography (20:1 DCM/MeOH) to yield **S9** (370 mg, 83%) or **S10** (365 mg, 80%) as white solid powder. ESI-MS: *m/z* 443.5 $[M+H]^+$ (**S9**), and *m/z* 457.5 $[M+H]^+$ (**S10**).

TFA (1 mL) was added dropwise into a solution of **S9** or **S10** (347 mg) in DCM (10 mL) at 0°C. The mixture was then allowed to room temperature and stirred for 1 h, followed by removing the solvent *under vacuo*. The residue was suspended in H_2O and the saturated $NaHCO_3$ was added with efficient stirring at 5°C, the resultant precipitate was filtered off and the filtrate (260 mg, 98%) was concentrated to dryness as white solid powder **S11** (277 mg, 97%) or **S12**(279 mg, 98%). ESI-MS: *m/z* 343.4 $[M+H]^+$ (**S11**), and *m/z* 357.4 $[M+H]^+$ (**S12**).

The **TSN** was dissolved in anhydrous DCM (10 mL), then the succinic anhydride (500 mg, 5 mmol) and 4-dimethylaminopyridine (244 mg, 2 mmol) were added. The mixture was then allowed to room temperature and stirred for 12 h. Then the solution was concentrated *in vacuo* and the residue was added EA (100 mL). The organic layer was washed with 1 N HCl (100 mL) and H_2O, dried over anhydrous Na_2SO4, and concentrated to yield white solid **S4** (640 mg, 95%), which was used without further purification. ESI-MS: *m/z* 674 $[M+H]^+$.

The **S11** or **S12** (329 mg, 1 mmol) and **S4** (674 mg, 1 mmol) were dissolved in anhydrous DMF (10 mL) and the solution was cooled to 0°C, then the HATU (570 mg, 1.5 mmol) and DIPEA (495 μL, 3.0 mmol) were added. The mixture was stirred at same temperature for 12 h and concentrated to yield white solid **TSM-4** (520 mg, 52%) or **TSM-5** (510 mg, 50%). ESI-MS: *m/z* 997.97 $[M+H]^+$ (**TSM-4**), and *m/z* 1013.96 $[M+H]^+$ (**TSM-5**).

Scheme V: Synthetic route of preparing TSM-6

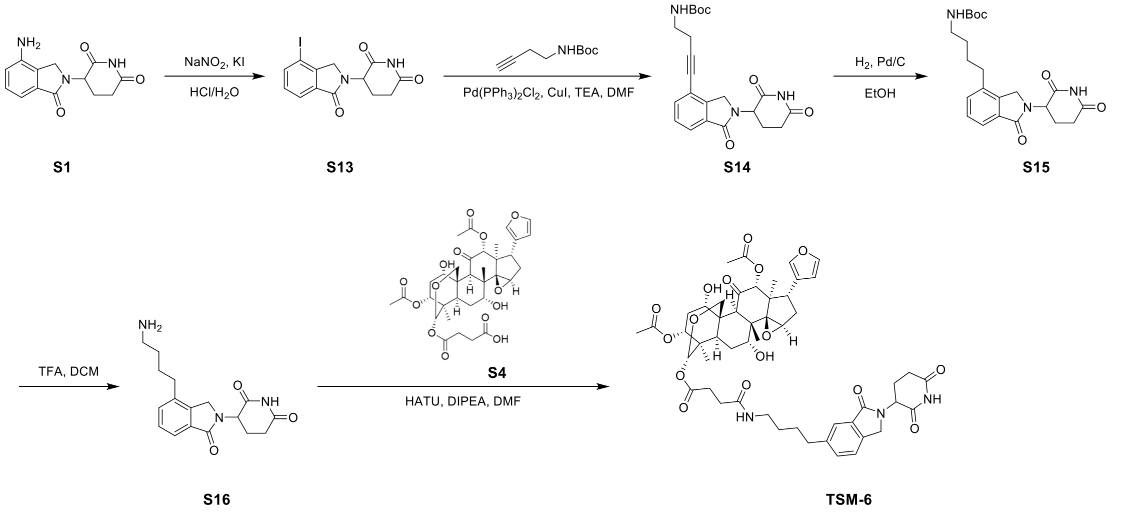

The **S1** (260 mg, 1 mmol) was dissolved in HCl/H_2O (5 mL/5 mL) and the solution was cooled to -5°C, then the aqueous solution of sodium nitrite (138 mg, 2 mmol in 1 mL H_2O) was added. The mixture was stirred at same temperature for 1 h and then the potassium iodide in aqueous solution (332 mg, 2 mmol in 1 mL H_2O) was added into the reaction solution and stirred for 12 h at room temperature, the concentrated to yield brown soild **S13** (250 mg, 67%). ESI-MS: m/z 371.1 [M+H]+.

A mixture of **S13** (370 mg, 1 mmol), cuprous iodide (380 mg, 5 mmol), trans-dichlorobis (triphenyl-phosphine) palladium (II) (105 mg, 0.15 mmol), and triethylamine (696 μL, 5 mmol) were dissolved in anhydrous DCM (5 mL), which was replaced by nitrogen 3 times. Then tert-butyl carbamate (519 μL, 3 mmol)) was added into the reaction solution and stirred for 5 h at 85°C. When the reaction was complete, the mixture was then diluted by EA, washed with H_2O, concentrated, and purified by column chromatography (20:1 DCM/MeOH) to yield **S14** (290 mg, 70%) as brown solid powder. ESI-MS: m/z 412.4 $[M+H]^+$.

The above **S14** (290 mg, 0.71 mmol) was dissolved in methanol (10 mL) and 10% palladium carbon (100 mg). After being replaced with hydrogen for three times, the mixture was stirred at room temperature until the reaction was complete. The crude product **S15** (280 mg, 90%) was obtained by adding diatomite for extraction and filtration, followed by washing with methanol for three times. ESE-MS: *m/z* 416.4 $[M+H]^+$.

S16 was prepared following the procedure described for **S3** as white solid product (302 mg, 96%), ESI-MS: *m/z* 316.4 $[M+H]^+$。

TSM-6 was prepared following the procedure described for **TSM-1** as white solid product (490 mg, 50%), ESI-MS: *m/z* 970.94 $[M+H]^+$。

Scheme Ⅵ: Synthetic route of preparing TSM-Me

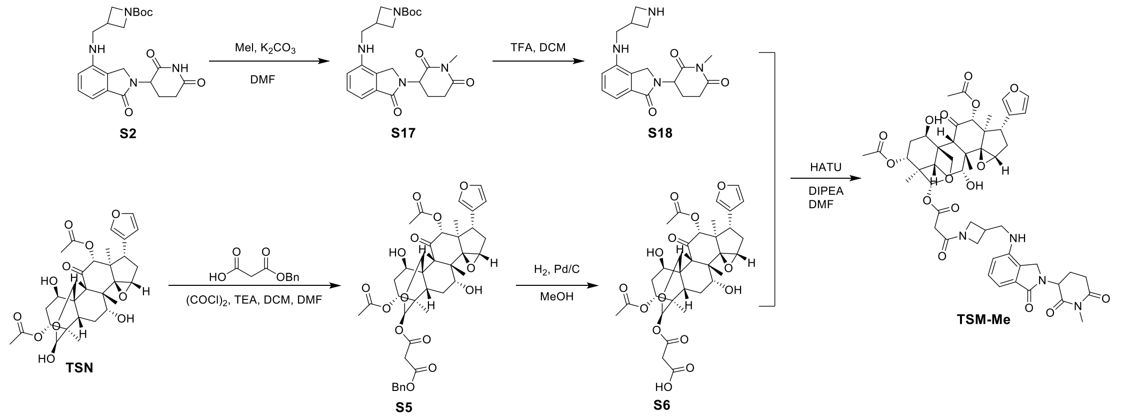

The **S2** (428 mg, 1.0 mmol) and K2CO3 (207 mg, 1.5 mmol) were dissolved in 10 mL of DMF, MeI (156 mg, 1.1 mmol) were added. The reaction solution was stirred at room temperature for 20 h and monitored by TLC. Upon completion, the reaction then poured into 30 mL of water, and extracted with ethyl acetate, organic layer was combined and washed with water and brine. The solution was dried over anhydrous Na2SO4, filtered and concentrated under reduced pressure, the crude product was purified by silica gel column chromatography with DCM and MeOH (DCM/MeOH = 30:1-20:1) to give white solid **S17** (267 mg, 60%), ESI-MS: *m/z* 443.5 $[M+H]^+$. ^{1}H NMR (400 MHz, Chloroform-d) δ 7.30 (t, *J* = 7.7 Hz, 1H), 7.19 (d, *J* = 7.4 Hz, 1H), 6.74 (d, *J* = 8.0 Hz, 1H), 5.09 (dd, *J* = 13.3, 5.1 Hz, 1H), 4.21 – 4.05 (m, 2H), 4.04 – 3.98 (m, 2H), 3.64 (dd, *J* = 8.7, 5.0 Hz, 2H), 3.39 (d, *J* = 7.3 Hz, 2H), 3.07 (s, 3H), 2.90 – 2.72 (m, 3H), 2.21 – 2.10 (m, 1H), 2.09 – 2.00 (m, 1H), 1.39 (s, 9H).

To **S17** (442 mg, 1.0 mmol) in DCM (3 mL) was added TFA (0.307 mL, 4.01 mmol) and the reaction mixture was stirred at room temperature for 3 h and then the solvent was removed and concentrated to dryness under vacuum. The crude product **S18** was directly used for the next step, ESI-MS: *m/z* 343.4 $[M+H]^+$.

To a solution of **S6** (67.4 mg, 0.1 mmol) in DMF (3 mL), 3 (41.0 mg, 0.12 mmol) HATU (57.0 mg, 0.15 mmol) and DIPEA (38.7 mg, 0.30 mmol) were added at 0 oC. Then the resulting reaction mixture was stirred at room temperature for 5 h and monitored by TLC. Upon completion, the reaction was then quenched with water and extracted with EtOAc. The organic layer was washed twice with water, dried over anhydrous Na2SO4, filtered and concentrated under reduced pressure. The crude material was purified by silica gel column chromatography using a DCM-MeOH gradient (DCM/MeOH = 40:1-12:1) to give the desired product **TSM-Me** as white powder (60 mg, yield 61%).^{1}H NMR (400 MHz, Chloroform-d) δ 7.31 (d, *J* = 5.2 Hz, 2H), 7.21 (d, *J* = 7.5 Hz, 1H), 7.09 (s, 1H), 6.76 (d, *J* = 8.0 Hz, 1H), 6.10 (s, 1H), 5.72 (s, 1H), 5.24 (s, 1H), 5.19 (s, 1H), 5.09 (d, *J* = 13.2 Hz, 1H), 4.59 (s, 1H), 4.30 – 4.20 (m, 5H), 4.07 (t, *J* = 8.5 Hz, 1H), 3.96 – 3.88 (m, 1H), 3.73 (d, *J* = 14.6 Hz, 2H), 3.66 – 3.57 (m, 2H), 3.43 (d, *J* = 6.6 Hz, 2H), 3.10 (s, 3H), 2.99 – 2.88 (m, 4H), 2.76 – 2.63 (m, 6H), 2.33

(d, *J* = 21.8 Hz, 3H), 2.20 – 2.09 (m, 2H), 2.03 (s, 3H), 1.95 (s, 3H), 1.87 (dd, *J* = 21.0, 9.9 Hz, 3H), 1.68 (d, *J* = 11.4 Hz, 1H), 1.29 (s, 3H), 1.08 (d, *J* = 5.7 Hz, 3H), 0.76 (s, 3H).13C NMR (100 MHz, CDCl3) δ 207.3, 171.6, 171.6, 171.5, 170.7, 170.4, 170.4, 170.3, 142.7, 142.5, 140.8, 132.2, 129.8, 127.0, 122.6, 113.0, 112.0, 95.0, 78.7, 73.6, 72.2, 70.0, 64.9, 58.7, 54.9, 53.5, 52.7, 51.6, 48.6, 47.0, 45.9, 45.6, 42.9, 42.6, 41.5, 39.3, 38.5, 35.2, 33.6, 32.1, 29.0, 28.1, 27.2, 25.6, 22.4, 21.5, 20.9, 19.2, 18.6, 17.3, 15.7, 12.5.

^{1}H-NMR analysis of **TSM-1**.

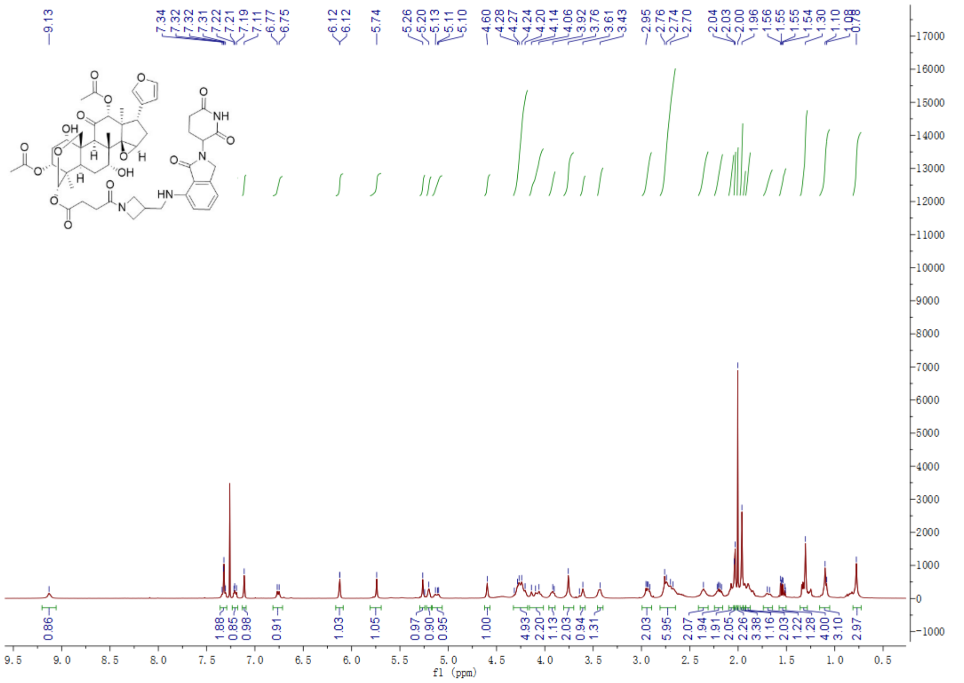

^{14}C-NMR analysis of **TSM-1**.

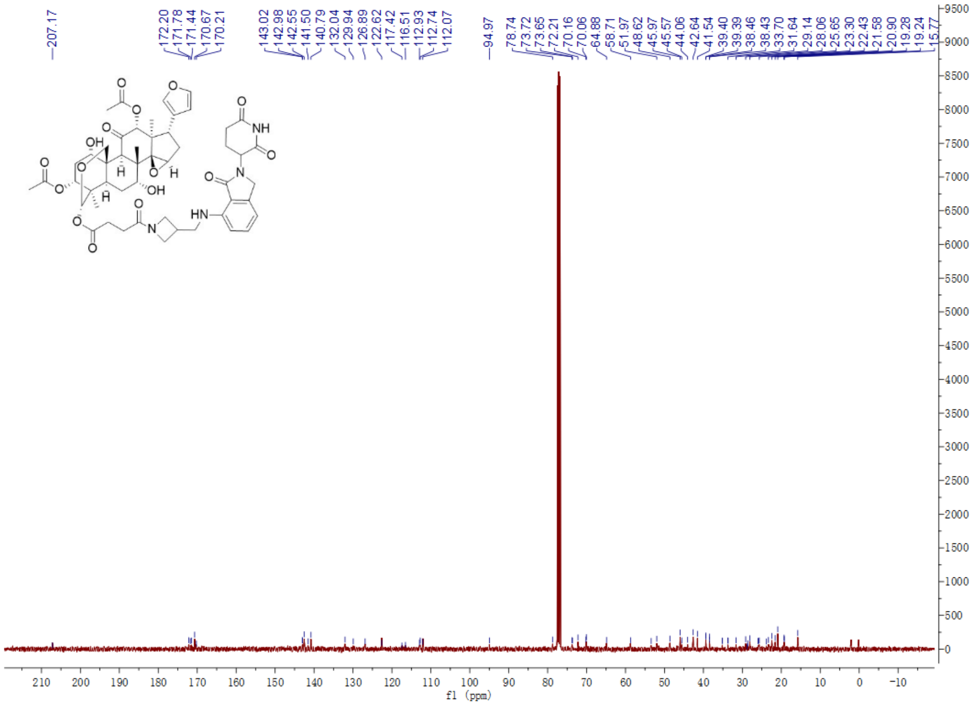

^{1}H-NMR analysis of **TSM-Me**.

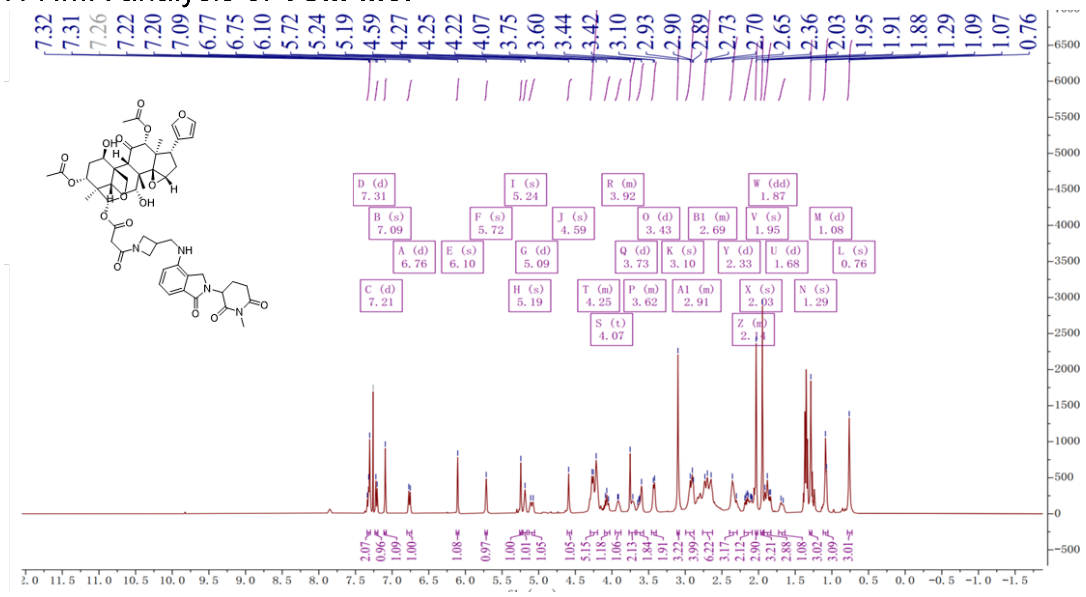

[14]C-NMR analysis of **TSM-Me**.

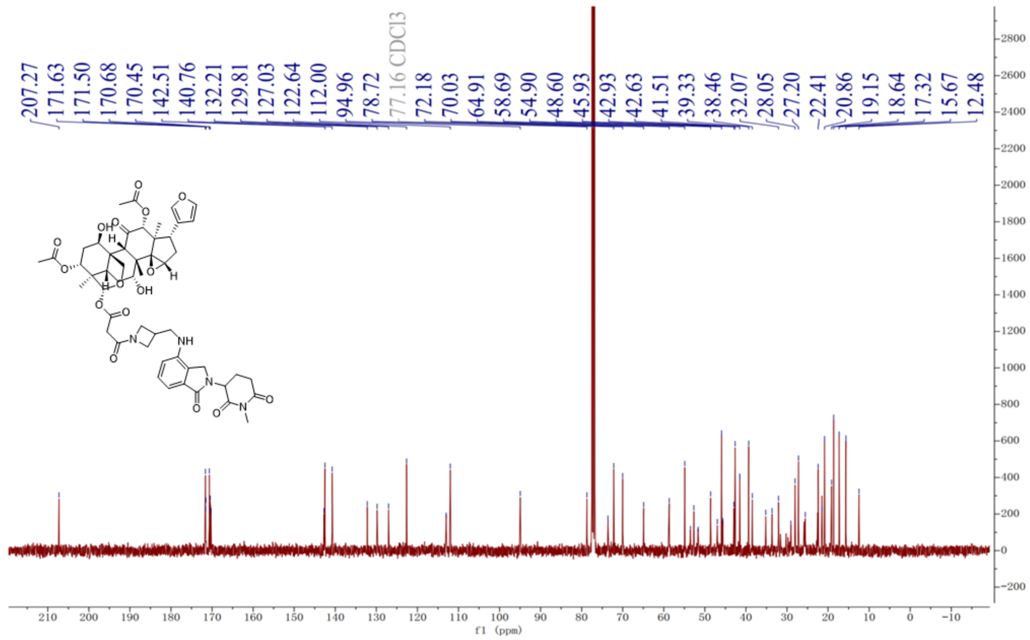

Liquid chromatograph-mass spectrometer analysis.

TSM-1

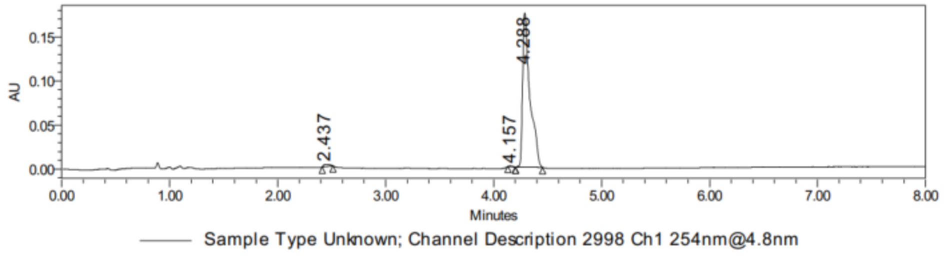

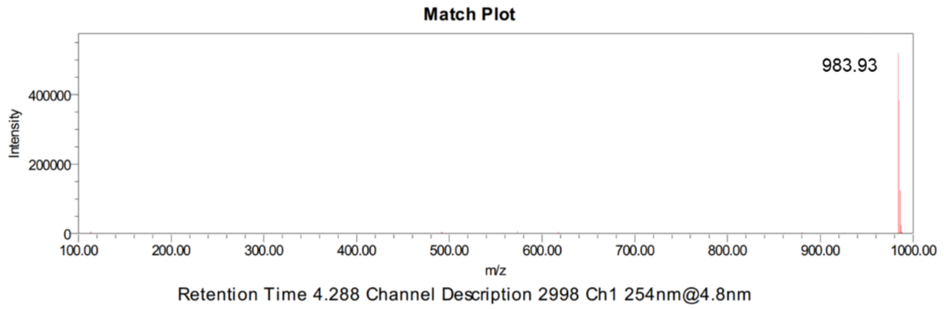

TSM-2

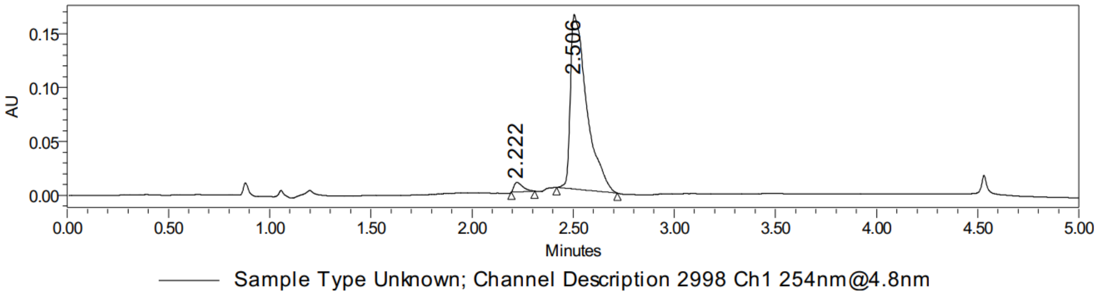

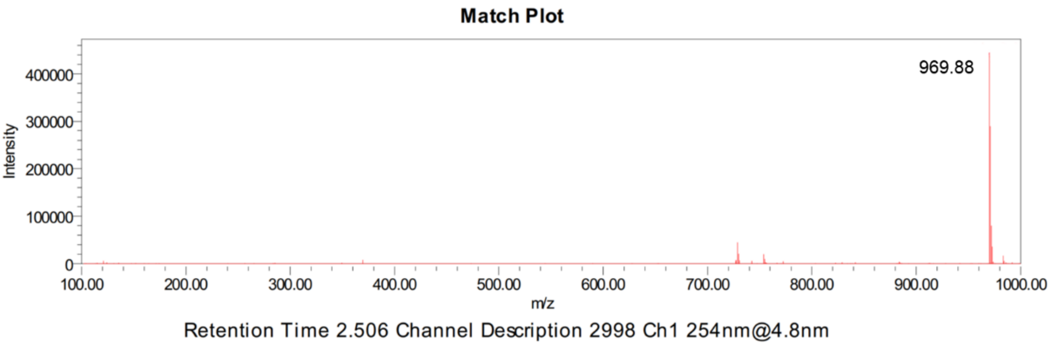

TSM-3

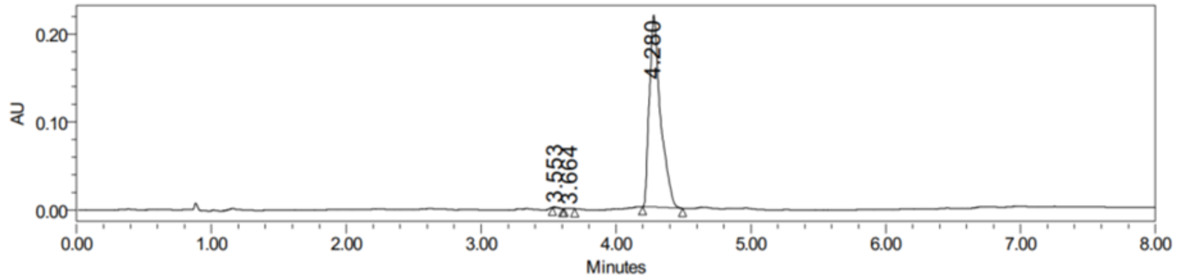

Sample Type Unknown; Channel Description 2998 Ch1 254nm@4.8nm

Match Plot

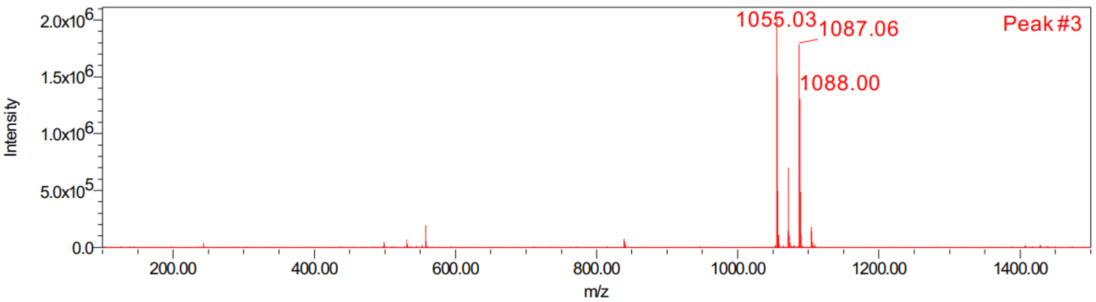

Retention Time 4.280 Channel Description 2998 Ch1 254nm@4.8nm

TSM-4

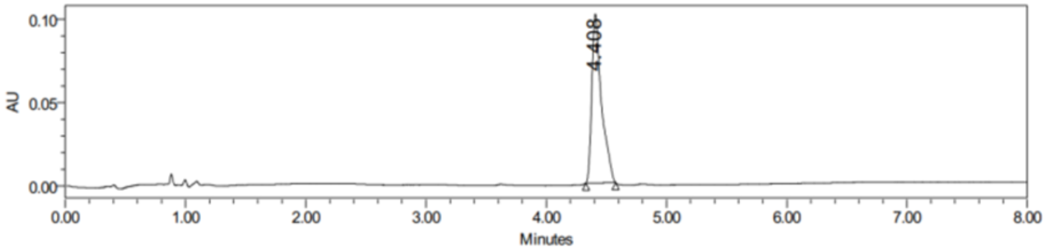

Sample Type Unknown; Channel Description 2998 Ch1 254nm@4.8nm

Match Plot

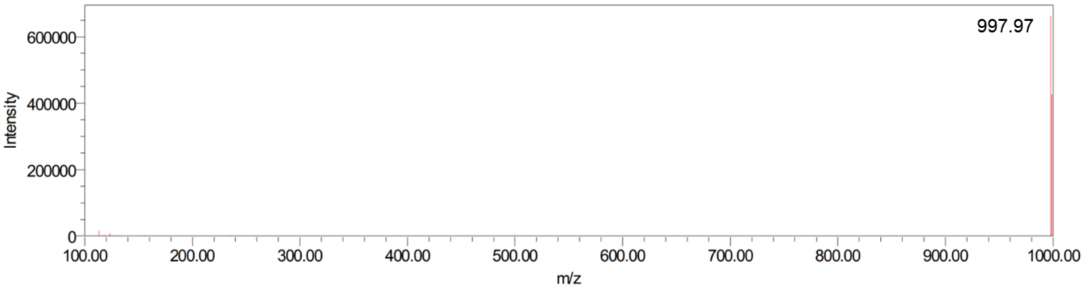

Retention Time 4.408 Channel Description 2998 Ch1 254nm@4.8nm

TSM-5

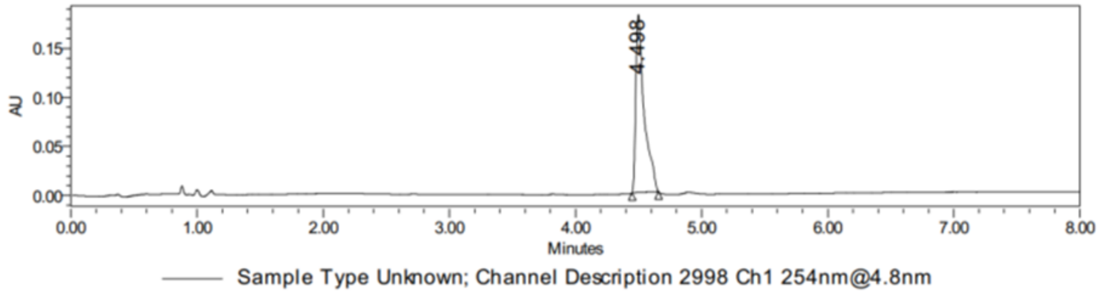

4.498
AU
0.15
0.10
0.05
0.00
0.00
1.00
2.00
3.00
4.00
5.00
6.00
7.00
8.00
Minutes
Sample Type Unknown; Channel Description 2998 Ch1 254nm@4.8nm

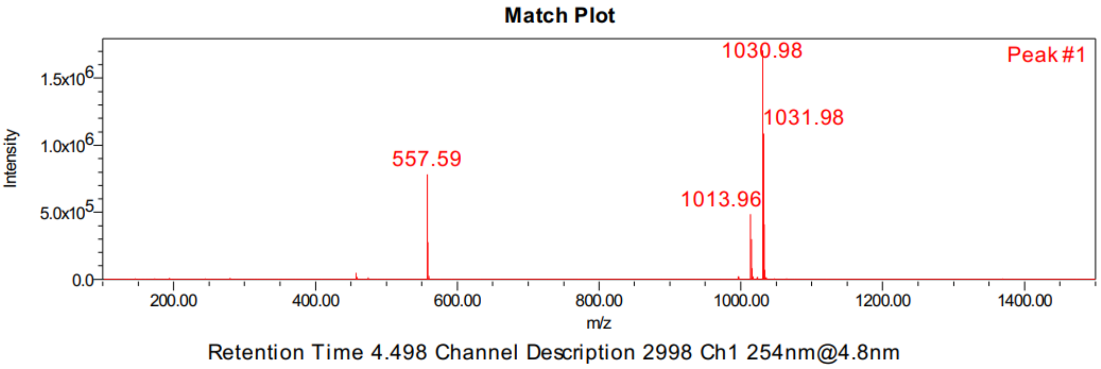

Match Plot
Peak #1
1030.98
1031.98
557.59
1013.96
Intensity
1.5x10^6
1.0x10^6
5.0x10^5
0.0
200.00
400.00
600.00
800.00
1000.00
1200.00
1400.00
m/z
Retention Time 4.498 Channel Description 2998 Ch1 254nm@4.8nm

TSM-6

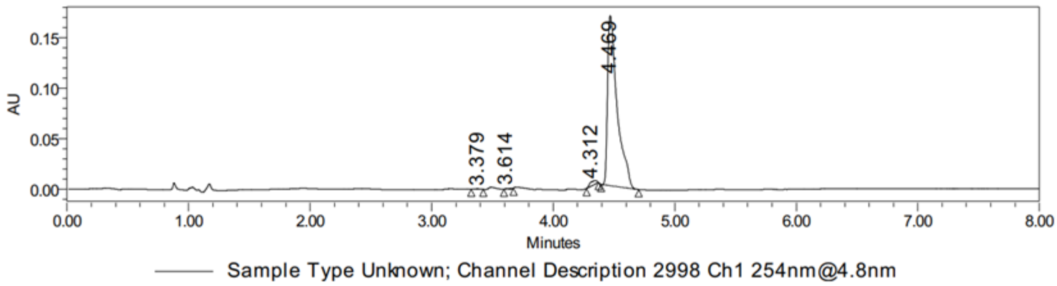

4.469
3.379
3.614
4.312
AU
0.15
0.10
0.05
0.00
0.00
1.00
2.00
3.00
4.00
5.00
6.00
7.00
8.00
Minutes
Sample Type Unknown; Channel Description 2998 Ch1 254nm@4.8nm

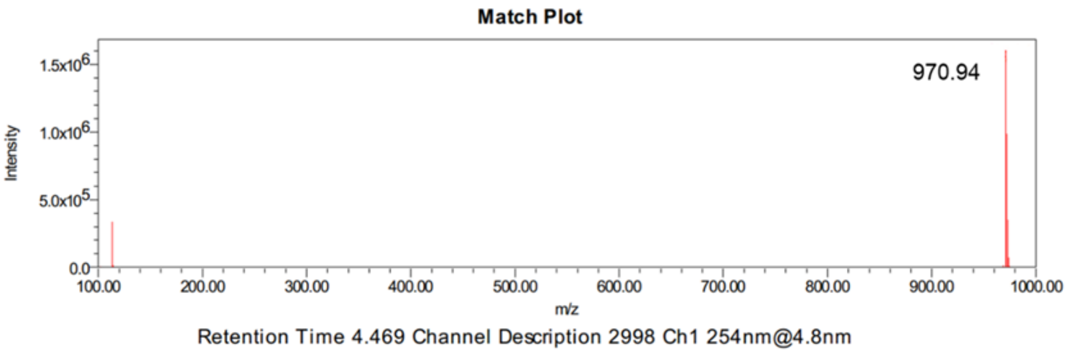

Match Plot
970.94
Intensity
1.5x10^6
1.0x10^6
5.0x10^5
0.0
100.00
200.00
300.00
400.00
500.00
600.00
700.00
800.00
900.00
1000.00
m/z
Retention Time 4.469 Channel Description 2998 Ch1 254nm@4.8nm

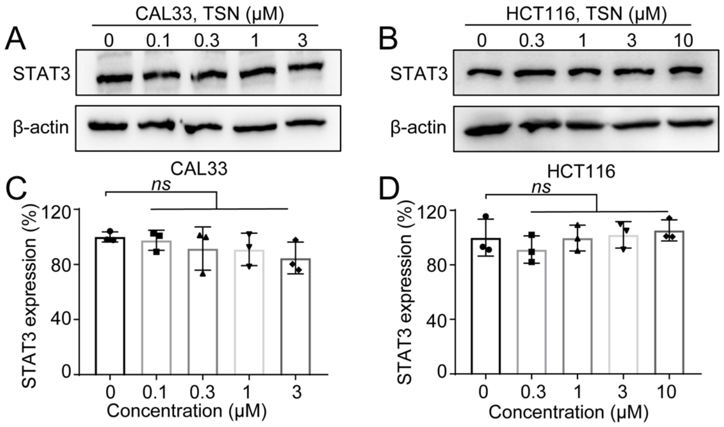

Figure S1. STAT3 protein expression. Western blot analysis showed that TSN treatment had no effect on STAT3 expression in CAL33 (A, C) and HCT116 (B, D) cells. n=3 replicates, ns: no statistical significance. P values are from ordinary one-way ANOVA with Dunnett's multiple comparison test (C, D).

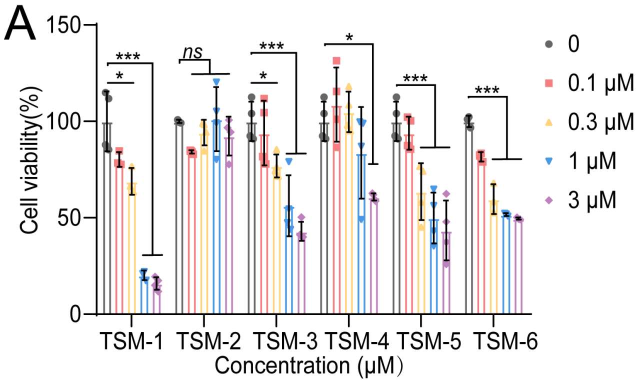

B

Drugs	IC_{50} (μM)
TSM-1	0.240
TSM-2	——
TSM-3	1.651
TSM-4	——
TSM-5	0.647
TSM-6	4.005

Figure S2. Anti-tumor effects of STAT3 degraders. IC_{50} (B) of CAL33 cells were detected when cells were treated with different TSMs for 48 h using CCK-8 assays (A) (n=3 replicates). Statistical significance: **p*<0.05, ***p*<0.01, ****p*<0.001 versus the control group, ns: no statistical significance. P values are from two-way ANOVA with Tukey's multiple comparison test (A).

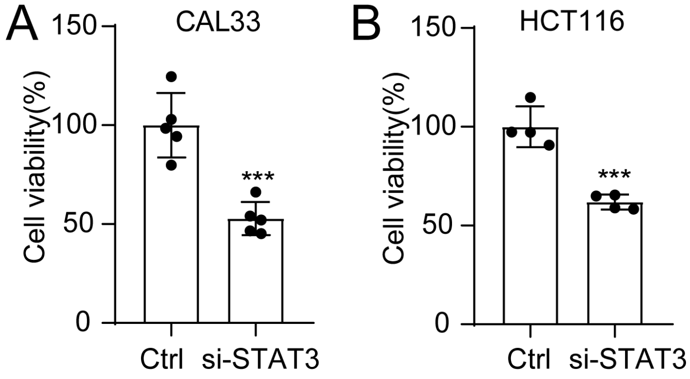

Figure S3. Cell viability of CAL33 and HCT116. (A) STAT3 knockdown through

siRNA (1878) for 48 h inhibited cell viability of CAL33 (n=5 replicates). (B) STAT3 knockdown through siRNA (1878) for 48 h inhibited cell viability of HCT116 (n=4 replicates). Statistical significance: ***p<0.001 versus the control group. P values are from Unpaired t test (A, B).

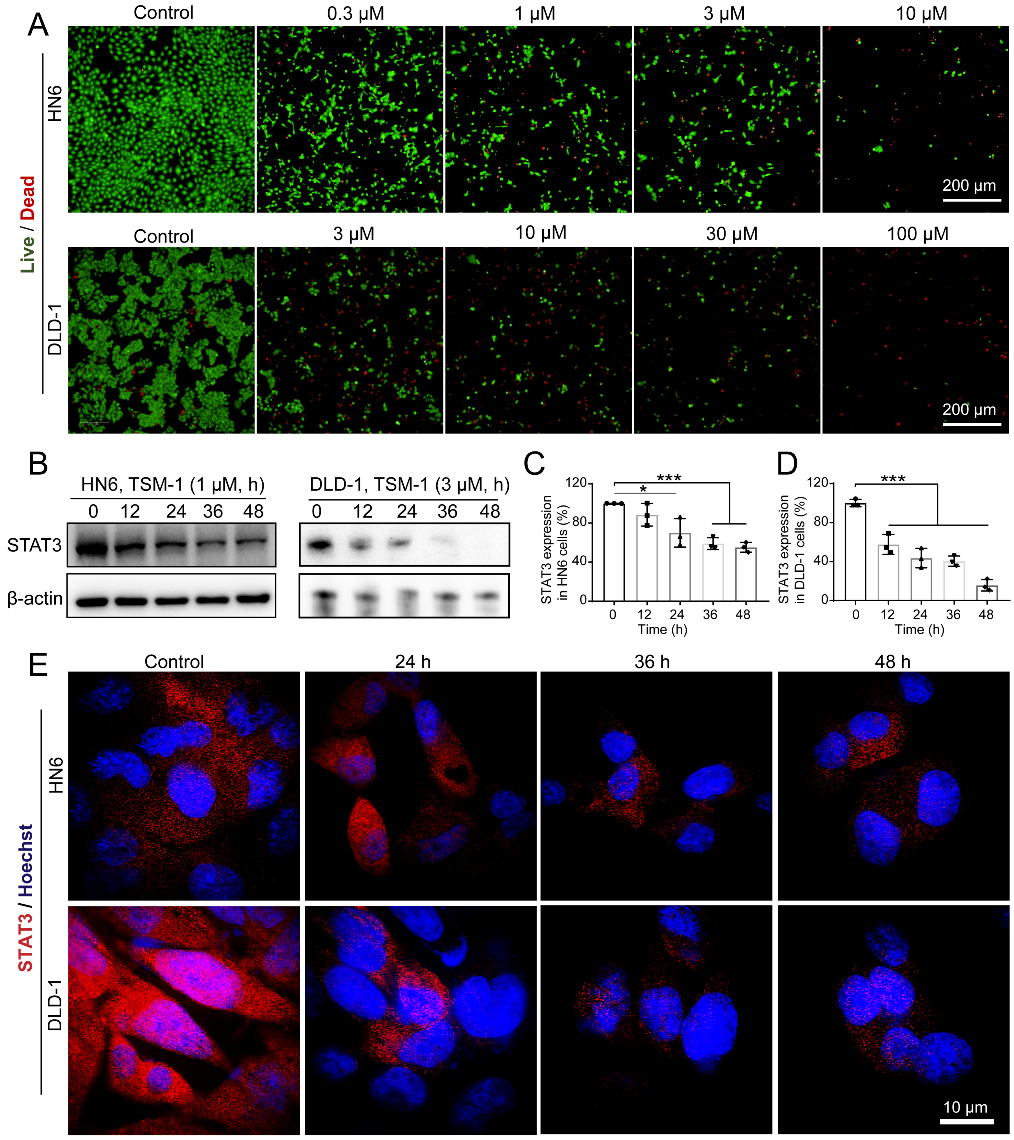

Figure S4. TSM-1 inhibited cell viability and degraded STAT3 protein in HN6 and DLD-1 cells. (A) HN6 and DLD-1 cells were stained with a live/dead cell viability/cytotoxicity kit after treatment with TSM-1 for 48 h (scale bar = 200 μm). (B) TSM-1 induced STAT3 degradation in a time-dependent manner in HN6 and DLD-1 cells. Quantitative results of the relative protein levels of STAT3 were shown in (C) (n=3 replicates) and (D) (n=3 replicates), and the representative microscopic photographs of the HN6 and DLD-1 cells were shown in (E) (scale bar＝10 μm). Statistical significance: *p < 0.05, ***p < 0.001 when compared to the control group. P values are from ordinary one-way ANOVA with Dunnett's multiple comparison test (C, D).

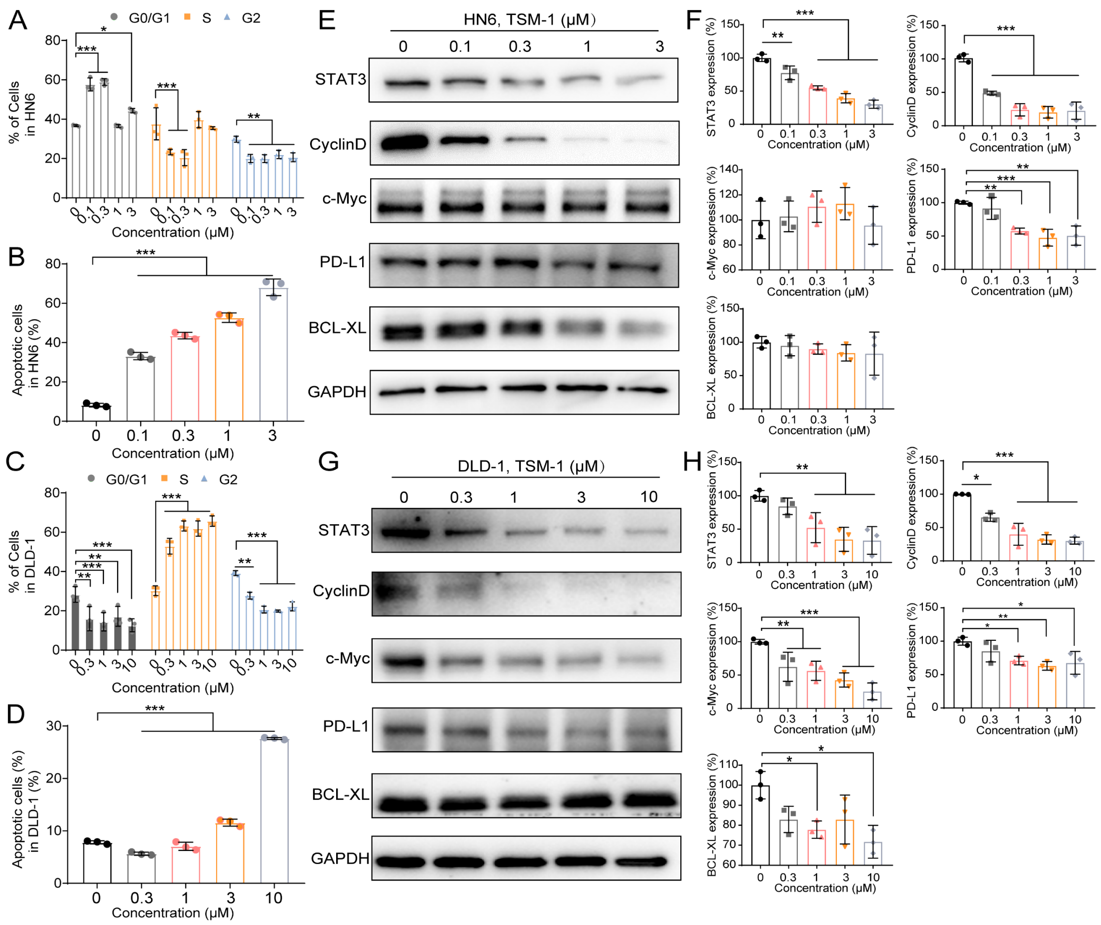

Figure S5. TSM-1 treatment elicited distinct cell cycle arrest and apoptosis. Cell cycle arrest was detected by flow cytometry after treatment with TSM-1 for 36 h (A, C) (n=3 replicates). And cell apoptosis was determined by flow cytometry after treatment with TSM-1 for 48 h (B, D) (n=3 replicates). TSM-1 treatment for 36 h reduced proteins expression of STAT3, cyclinD, c-Myc, PD-L1, and BCL-XL in HN6 (E, F) and DLD-1 (G, H) cells respectively (n=3 replicates). Statistical significance: * $p < 0.05$, ** $p < 0.01$, *** $p < 0.001$ versus the control group. P values are from two-way ANOVA with Tukey's multiple comparison test (A, C) or ordinary one-way ANOVA with Dunnett's multiple comparison test (B, D, F, H).

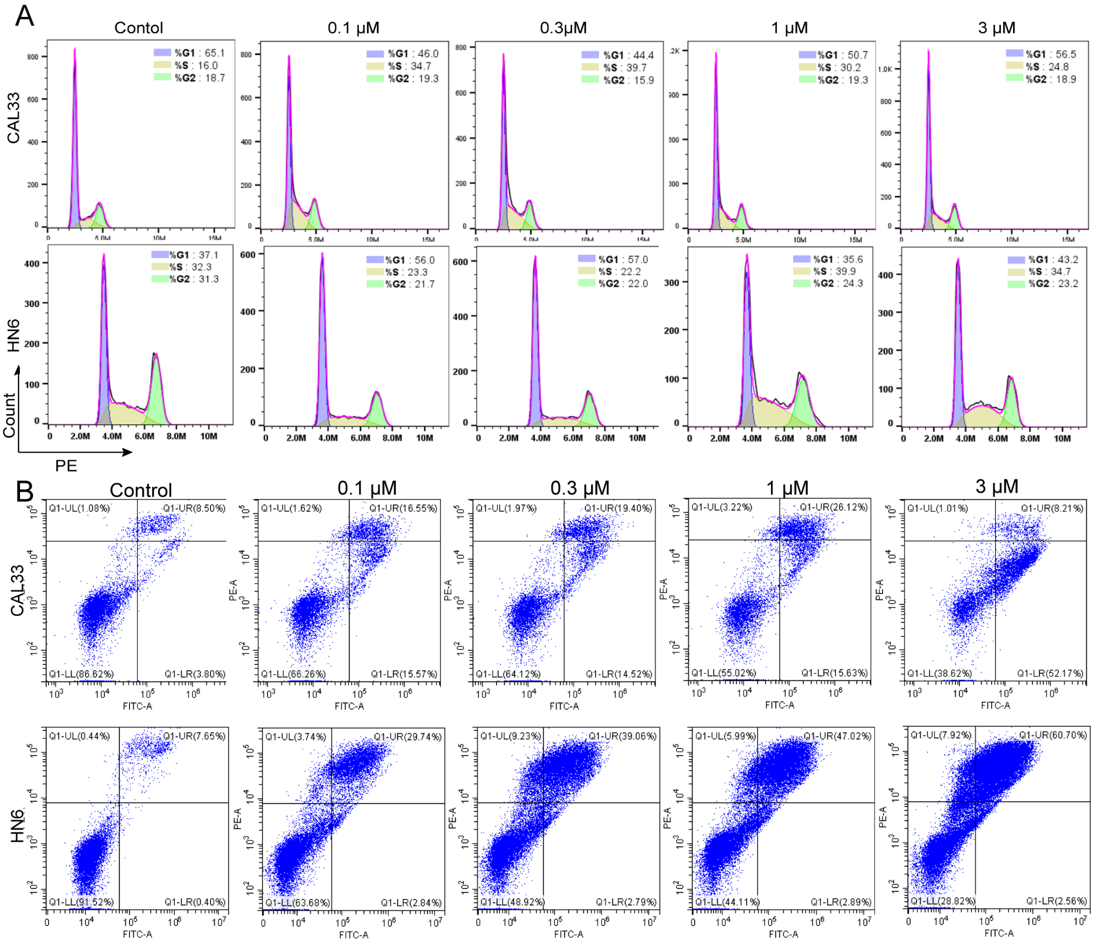

Figure S6. Cell cycle and apoptosis analysis in HNSCC cells. TSM-1 treatment induced cell cycle arrest (A) and apoptosis (B) at dose-dependent manner.

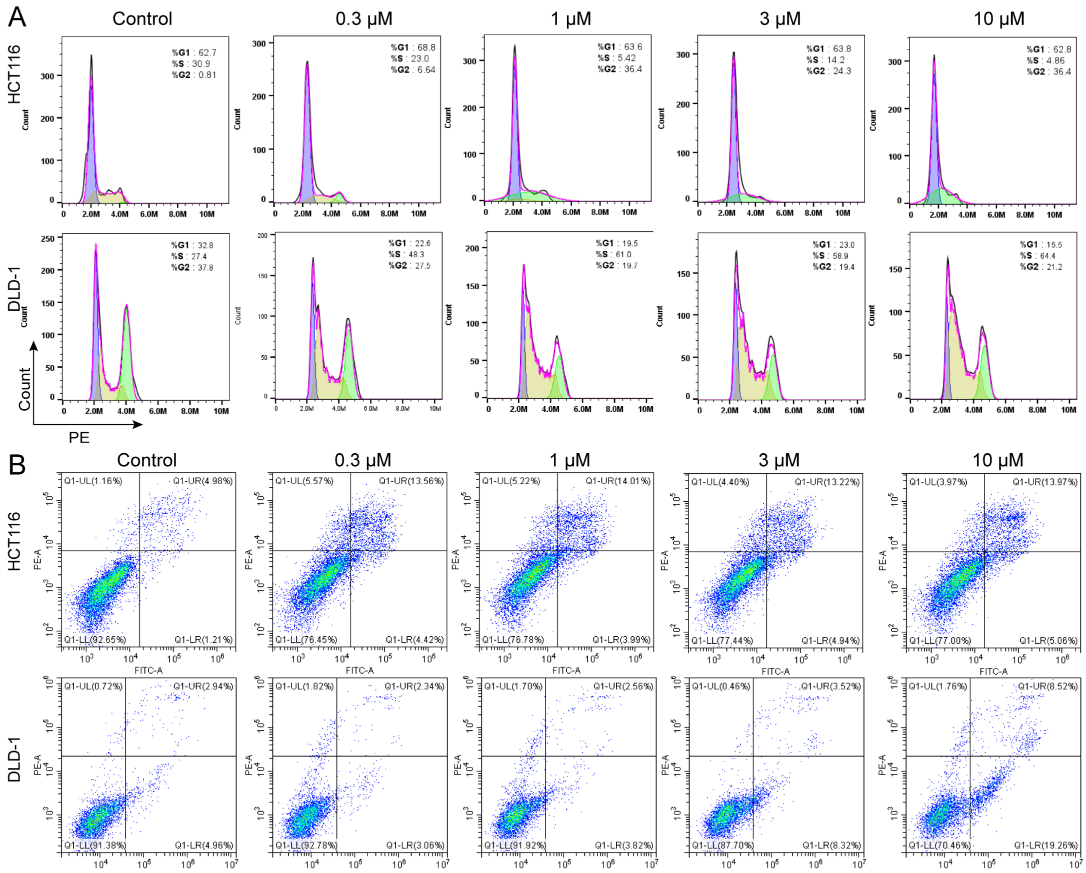

Figure S7. Cell cycle and apoptosis analysis in CRC cells. TSM-1 treatment induced cell cycle arrest (A) and apoptosis (B) at dose-dependent manner.

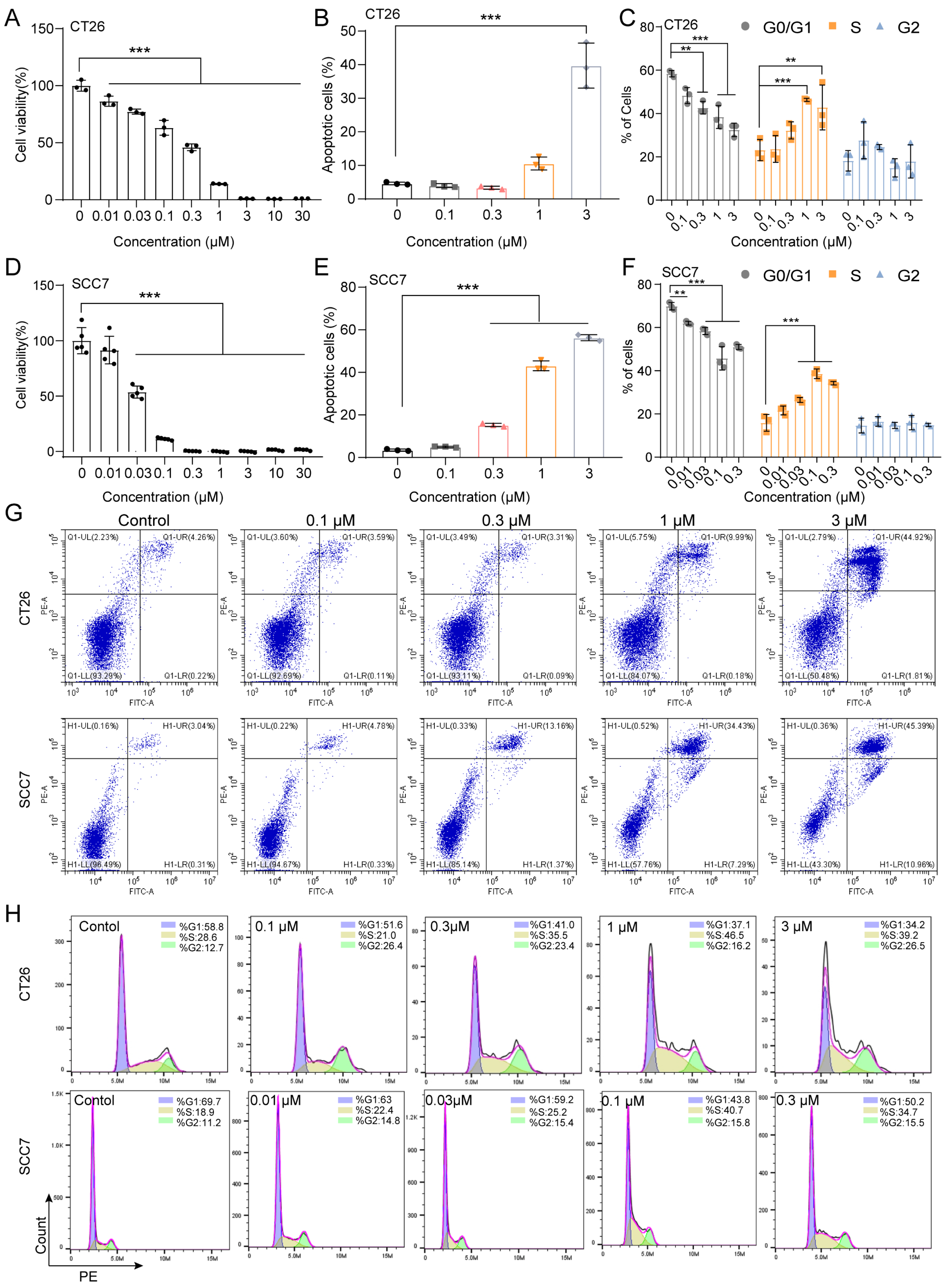

Figure S8. TSM-1 inhibited two murine cell lines *in vitro*. TSM-1 treatment inhibited cell viability of CT26 (A) and SCC7 (D) cells (n=3 replicates). TSM-1 treatment induced cell apoptosis of CT26 (B, G) (n=3 replicates) and SCC7 (E, G) (n=3 replicates). TSM-1 induced cell cycle arrest of CT26 (C, H) (n=3 replicates) and SCC7 (F, H) (n=3 replicates) cells. Statistical significance: ** $p < 0.01$, *** $p < 0.001$ versus the control group. P values are from ordinary one-way ANOVA with Dunnett's multiple comparison test (A, B, D, E) or two-way

ANOVA with Tukey’s multiple comparison test (C, F).

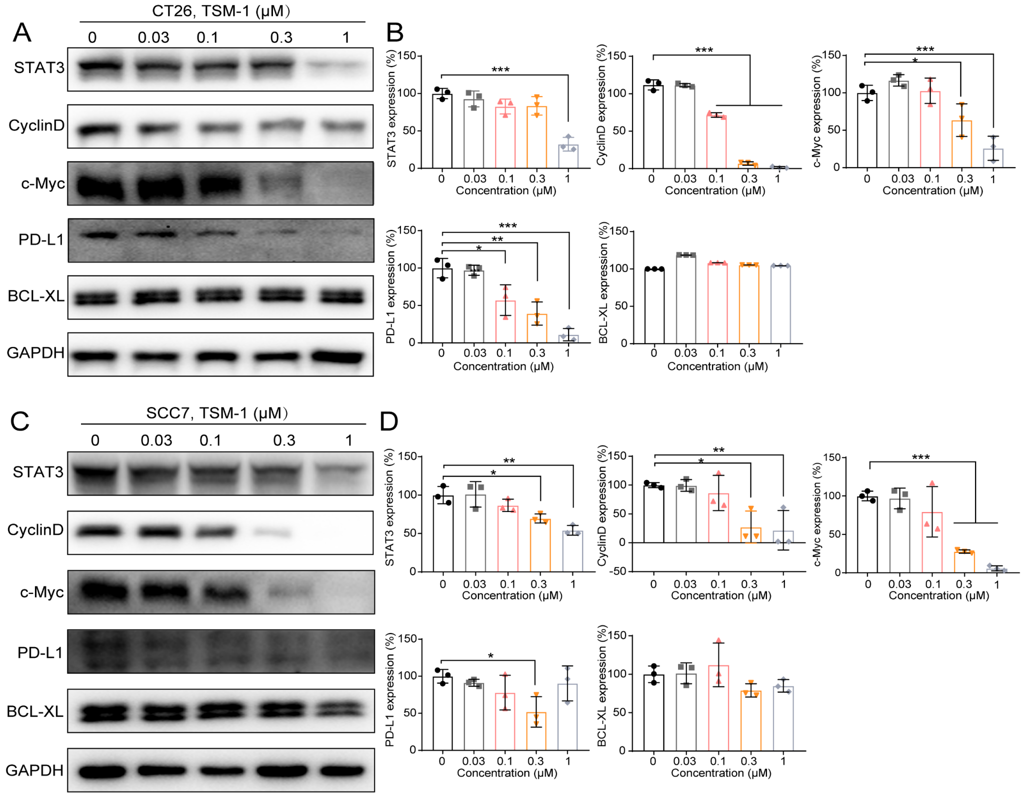

Figure S9. TSM-1 inhibited proteins expression in two murine cell lines. TSM-1 treatment for 36 h reduced proteins expression of STAT3, cyclinD, c-Myc, and PD-L1 in CT26 (A, B) and SCC7 (C, D) cells respectively (n=3 replicates). Statistical significance: * $p < 0.05$, ** $p < 0.01$, *** $p < 0.001$ versus the control group. P values are from ordinary one-way ANOVA with Dunnett’s multiple comparison test (B, D).

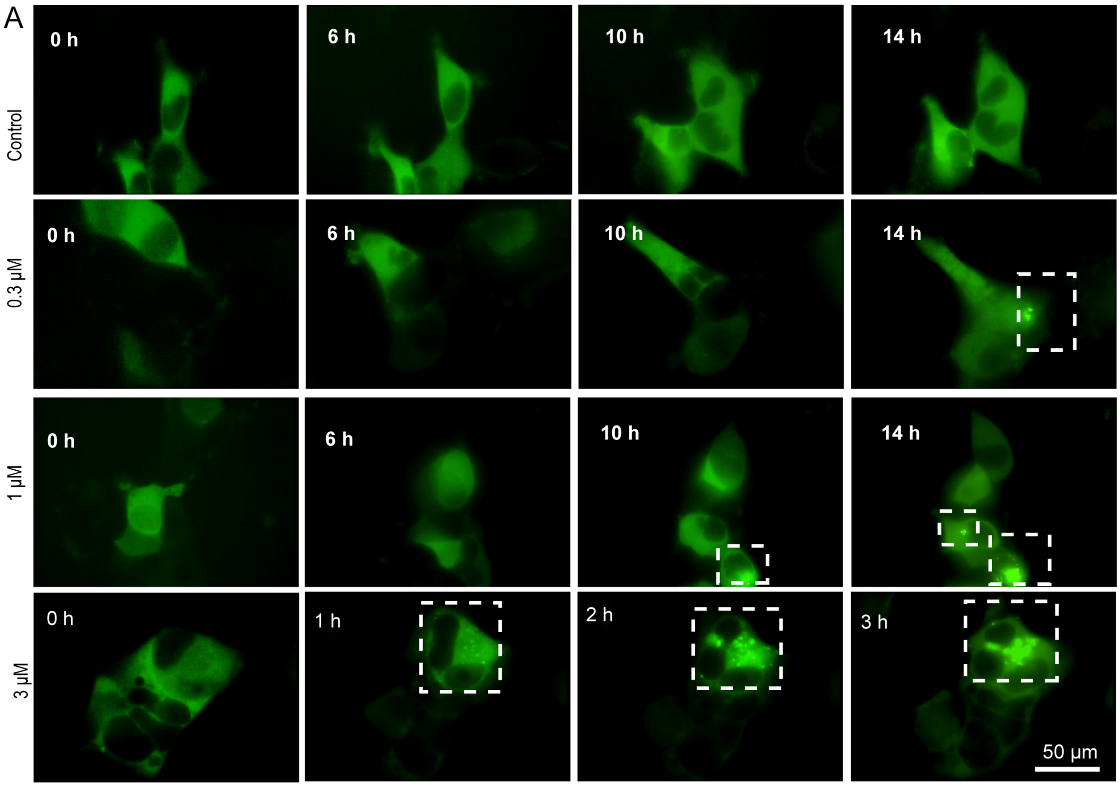

Figure S10. TSM-1 induced formation of ternary complexes in cells. (A) GFP-fluorescence images of HEK293T cells expressing CRBN-EGFP-HOTag3 and STAT3-EGFP-HOTag6 at the indicated time point after treatment with TSM-1.

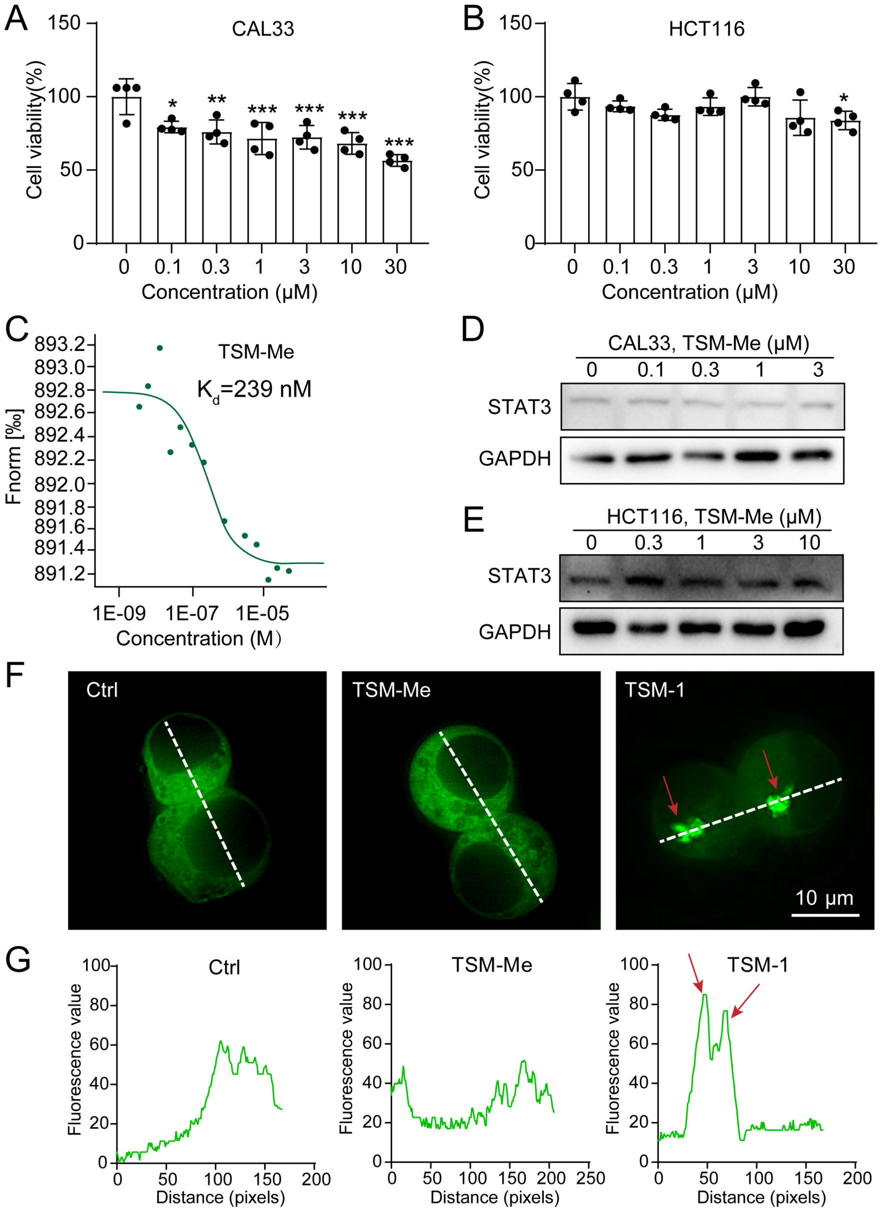

Figure S11. The mechanism of action of TSM-1. CAL33 (A) and HCT116 (B) cells proliferation were detected using CCK-8 assays after treatment with TSM-Me for 48 h (n=4 replicates). (C) MST analysis of TSM-Me binding to STAT3 (Kd = 239 nM). Western blot analysis showed that TSM-Me treatment had no effect on STAT3 expression in CAL33 (D) and HCT116 (E) cells. (F-G) SPPIER assays showed that TSM-Me couldn't induce the formation of the ternary complex when the 293T cells were treated with 1 μM TSM-Me for 48 h. Statistical significance: **p* < 0.05, ***p* < 0.01, ****p*<0.001 versus the control group. P values are from ordinary one-way ANOVA with Dunnett's multiple comparison test (A, B).

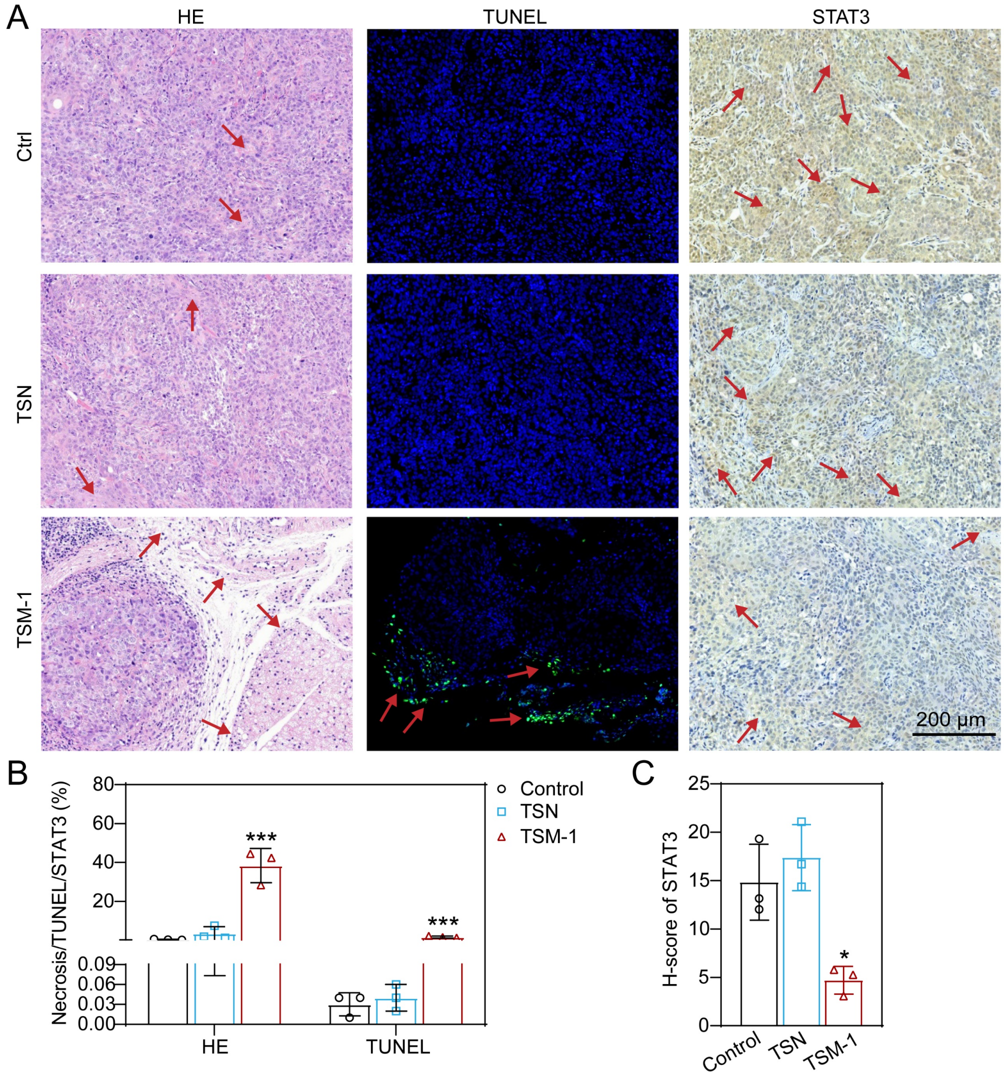

Figure S12. TSM-1 decreased STAT3 protein and induced tumor necrosis in HN6 tumor tissues. TSM-1 led to significantly increased necrosis area, TUNEL-positive cells and decreased STAT3 expression compared with control and TSN group in HN6 xenograft tumor model (A-C) (n=3 mice). Statistical significance: *p<0.05, ***p<0.001 versus the control group. P values are from ordinary one-way ANOVA with Dunnett's multiple comparison test (B, C).

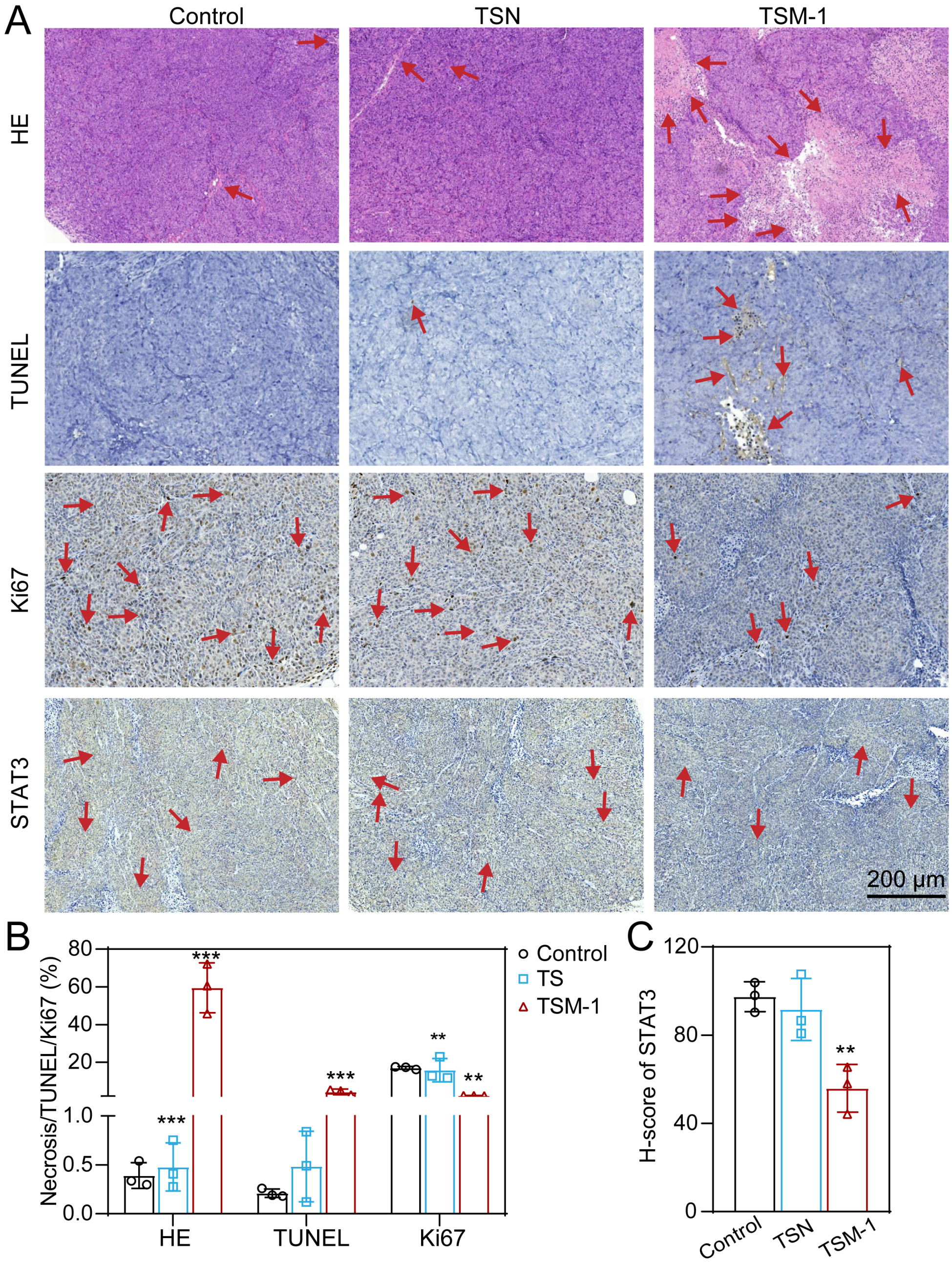

Figure S13. TSM-1 decreased STAT3 protein and induced tumor necrosis in HCT116 xenograft tumor model. (A) TSM-1 led to significantly increased necrosis area, TUNEL-positive cells, decreased STAT3 expression, and Ki67 positive cells compared with control or TS group. The statistical analysis results were shown in (B) and (C) (n=3 mice). Statistical significance: **p<0.01, ***p<0.001 versus the control group. P values are from ordinary one-way ANOVA with Dunnett's multiple comparison test (B, C).

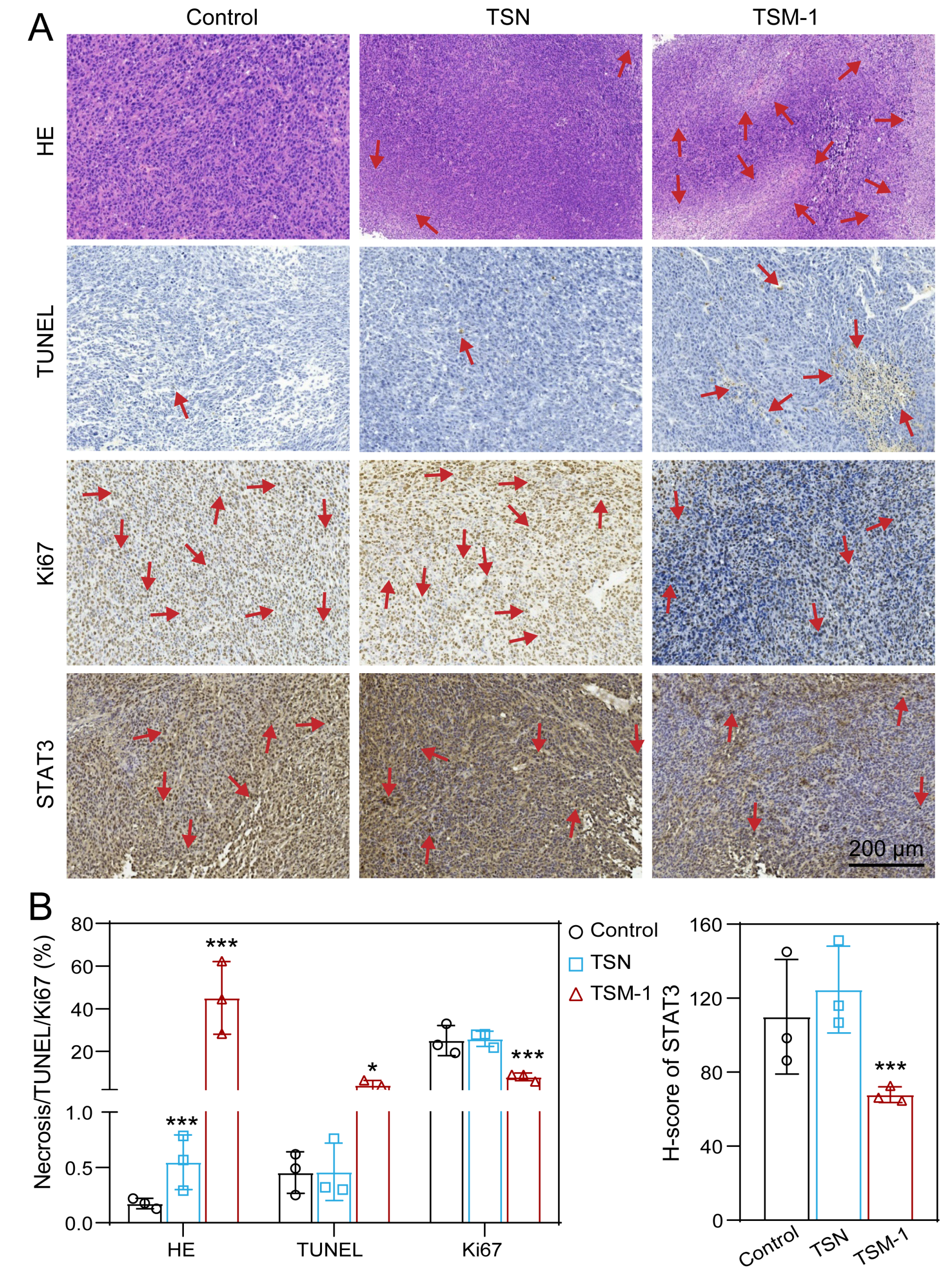

Figure S14. TSM-1 decreased STAT3 protein and induced tumor necrosis in CT26 xenograft tumor model. (A) TSM-1 led to significantly increased necrosis area, TUNEL-positive cells, decreased STAT3 expression and Ki67 positive cells compared with control or TS group. The statistical analysis results were shown in (B) and (C). n=3 mice, statistical significance: *p<0.05, **p<0.01, ***p<0.001 versus the control group. P values are from ordinary one-way ANOVA with Dunnett's multiple comparison test (B).

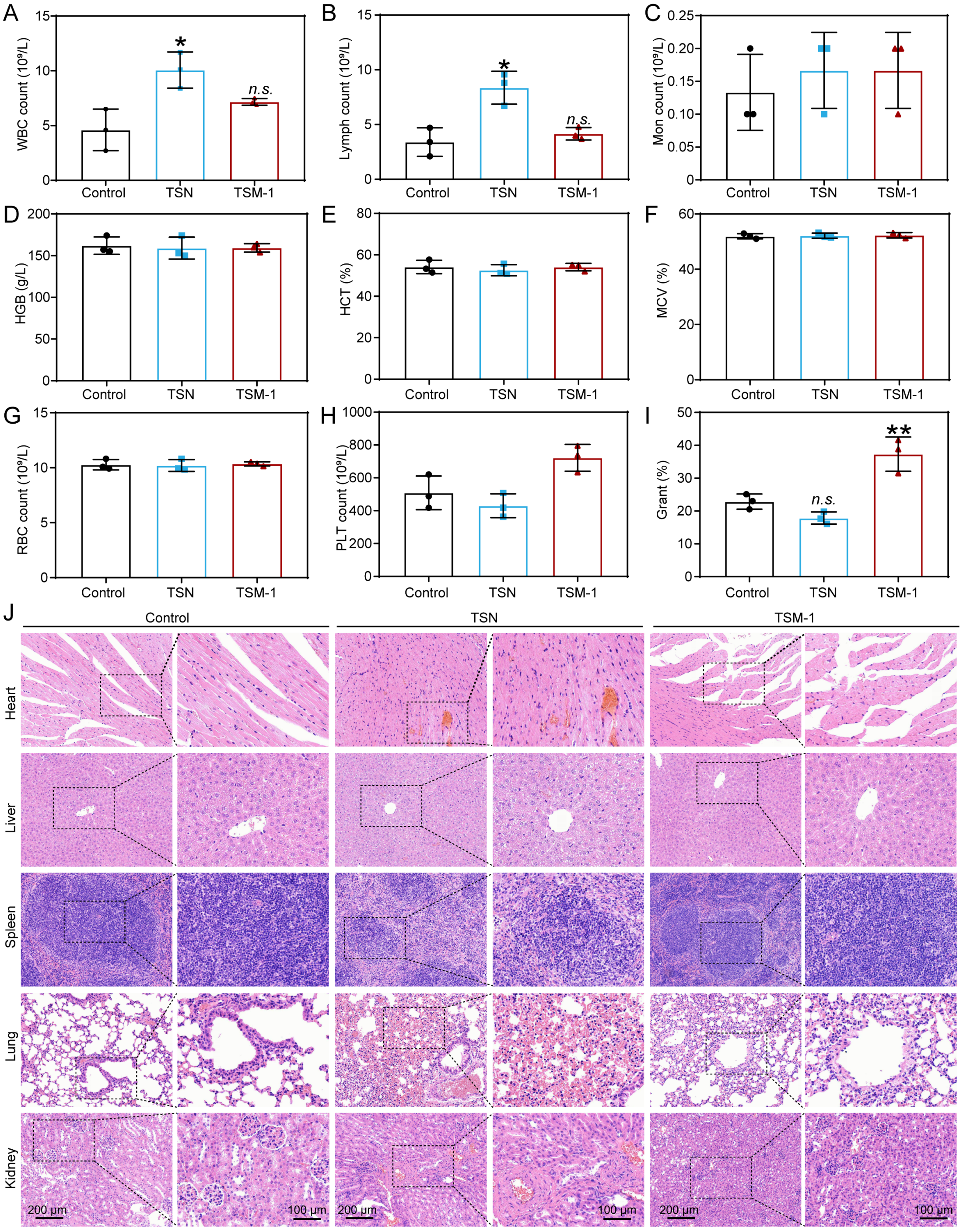

Figure S15. Safety investigation of TSM-1. (A) WBC count. (B) Lymph count. (C) Mon count. (D) HGB count. (E) HCT count. (F) MCV count. (G) RBC count. (H) PLT count. (I) Grant count. (J) Immunohistochemical staining of major organs. n=3 mice, statistical significance: *p<0.05, **p<0.01 when compared to the control group, ns: no statistical significance. P values are from ordinary one-way ANOVA with Dunnett's multiple comparison test (A-I).

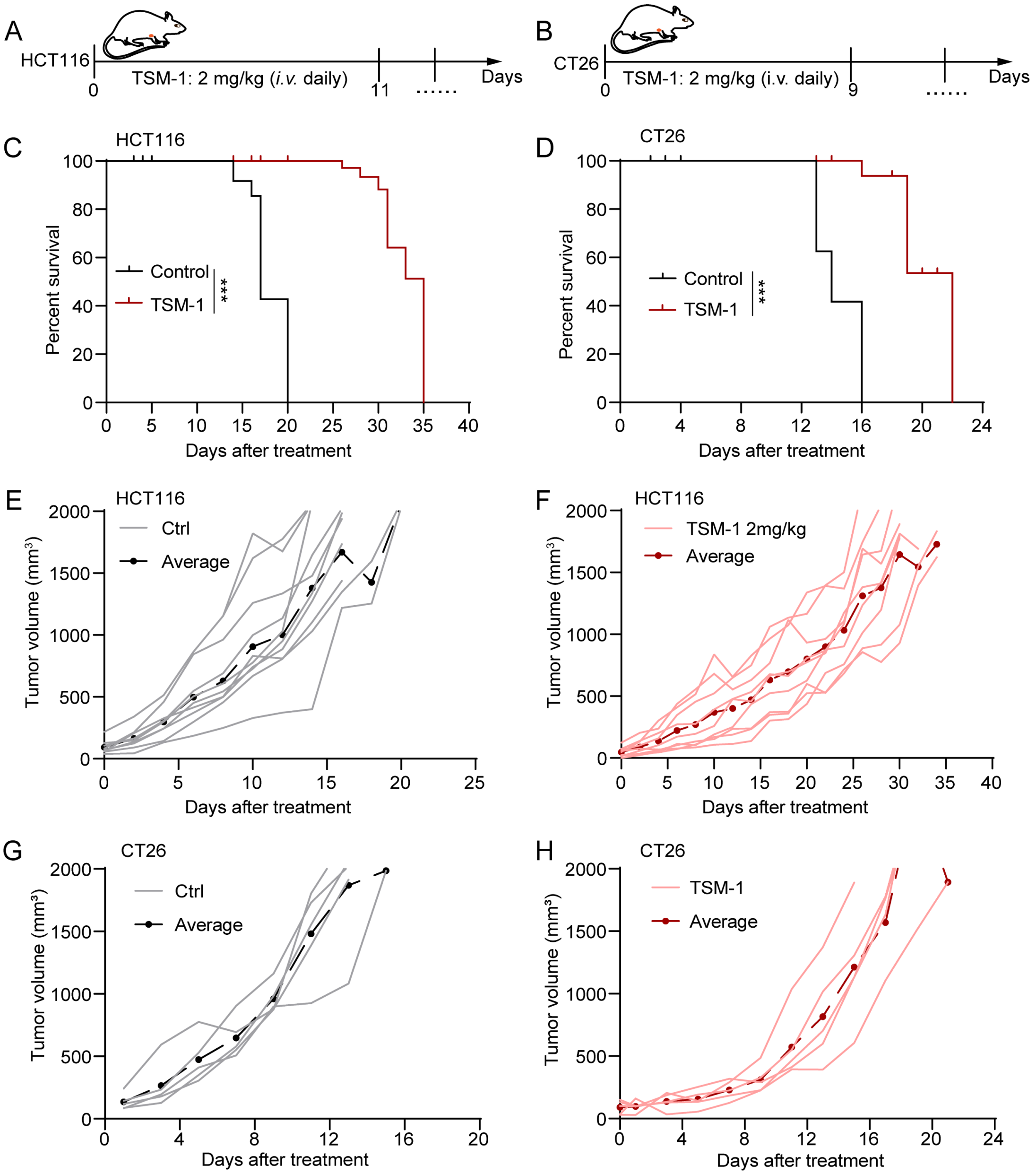

Figure S16. TSM-1 extended the survival of the CT26 and HCT116 tumor-bearing mice. (A) and (B), the treatment regimen diagrams. (C) Percent survival of HCT116 tumor-bearing mice. (D) Percent survival of CT26 tumor-bearing mice. (E) Tumor volume of per HCT116 tumor-bearing mice in the control group and TSM-1-treatment (F). (G) Tumor volume of per HCT116 tumor-bearing mice in the control group and TSM-1-treatment (H). n=5 mice, statistical significance: ***p<0.001 when compared to the control group. P values are from survival analyses (C, D).

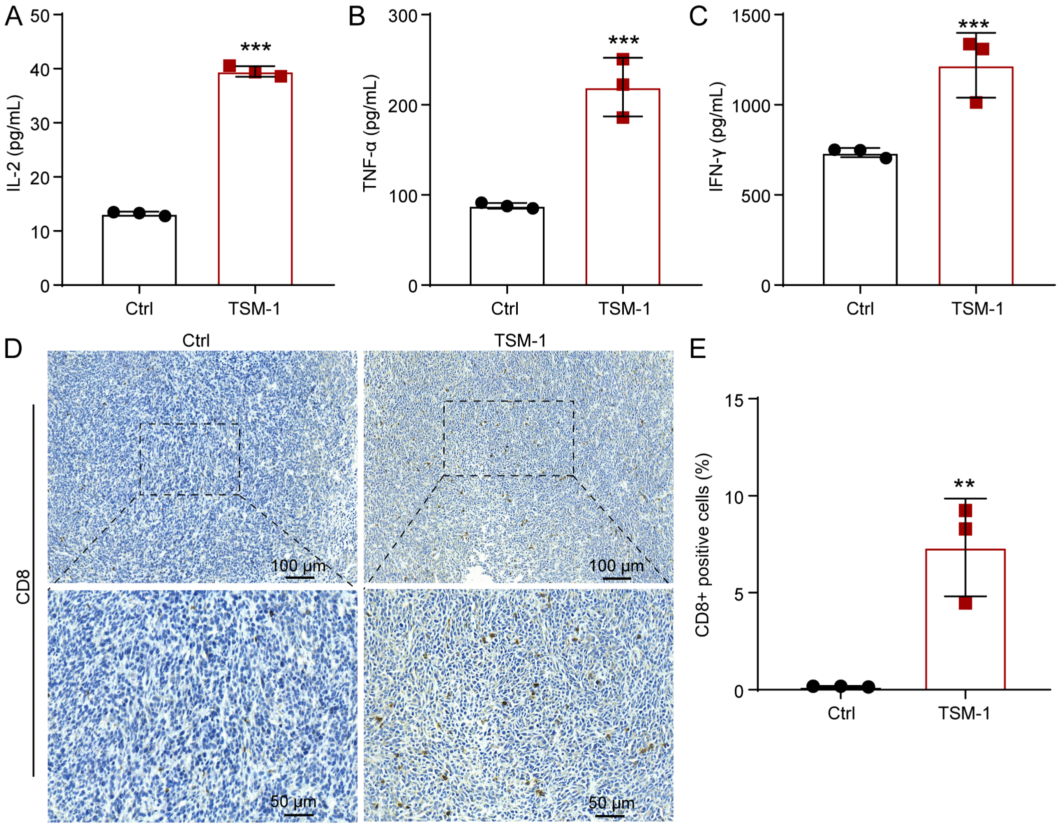

Figure S17. TSM-1 induced T cell activation and infiltrates. (A) The level of IL-2 in CT26 tumor tissues. (B) The level of TNF-α in CT26 tumor tissues. (C) The level of IFN-γ in CT26 tumor tissues. (D) TSM-1 led to increased infiltration of CD8+ T cells. The statistical analysis results were shown in (E). n=3 mice, statistical significance: **p<0.01, ****p*<0.001 when compared to the control group. P values are from Unpaired t test (A, B, C, E)

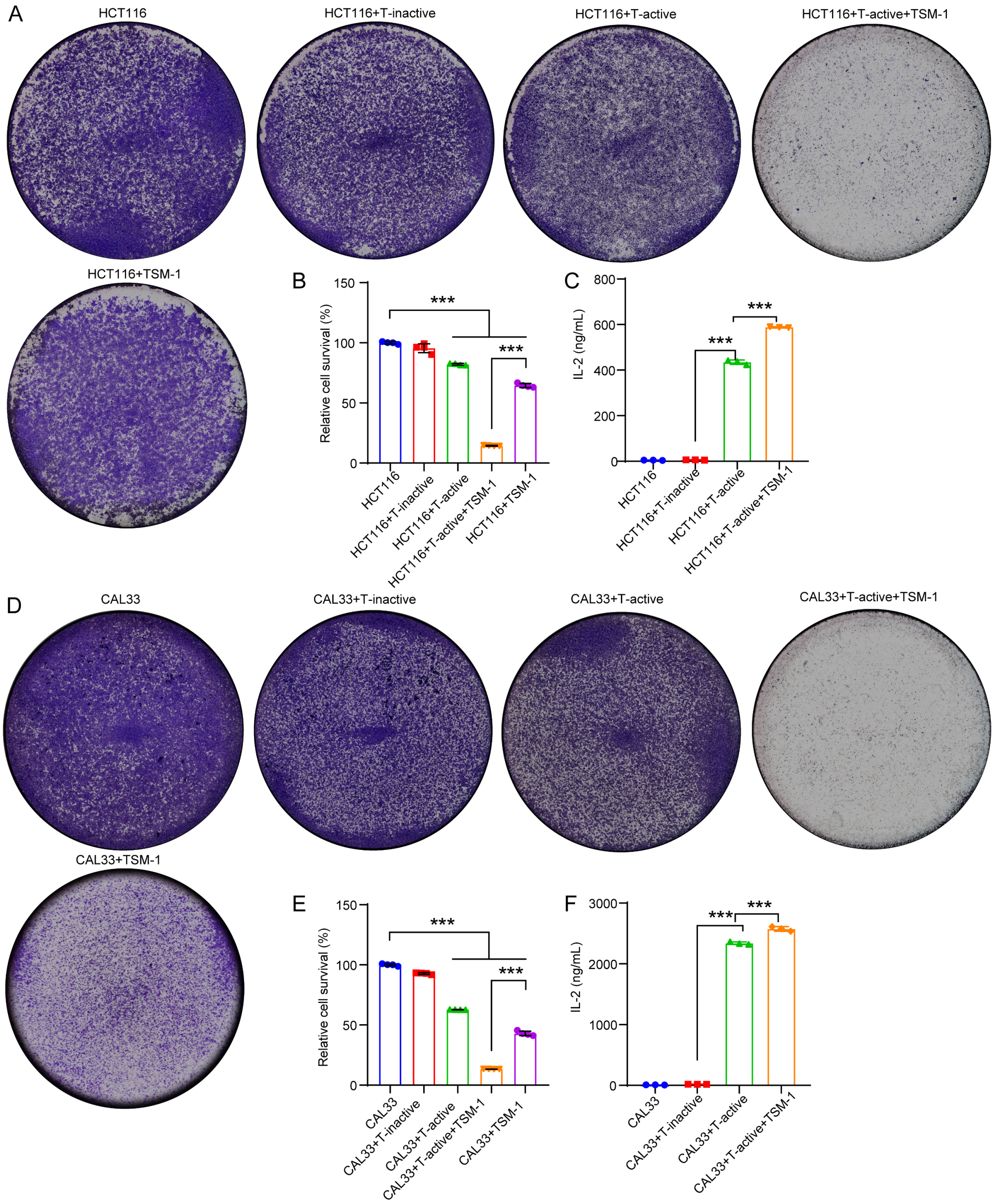

Figure S18. TSM-1 induced T cell activation and infiltrates *in vitro*. (A) HCT116 cells, pretreated with TSM-1 for 24 h, were cocultured with activated Jurkat T cells (tumor: Jurkat T =1:6) for 24 h and subsequently stained with crystal violet for imaging. (B) Crystal violet was dissolved in acetic acid and measured at 595 nm for cell viability detection of HCT116 cells (n=3 replicates). (C) IL-2 levels in the supernatant of A were measured by ELISA kits (n=3 replicates). (D) CAL33 cells, pretreated with TSM-1 for 24 h, were cocultured with activated Jurkat T cells (ratio=1:1) for 24 h and subsequently stained with crystal violet for imaging. (E) Crystal violet was dissolved in acetic acid and measured at 595 nm for cell viability detection of CAL33 cells (n=3 replicates). (F) IL-2 levels in the supernatant of D were measured by ELISA kits (n=3 replicates). Statistical significance: ***$p<0.001$ when compared to the control group. P values are from

two-way ANOVA with multiple comparison test (B, C, E, F).

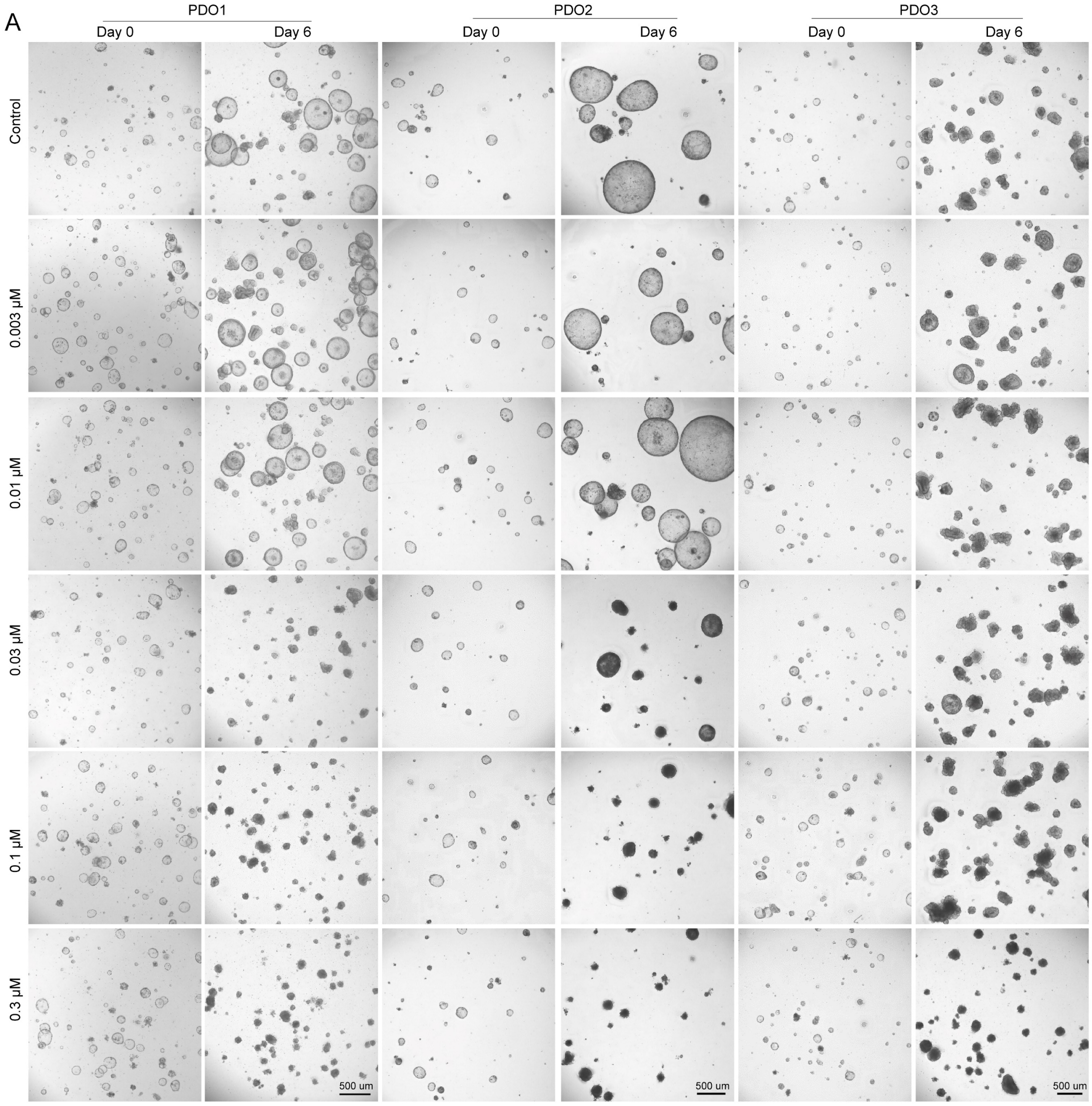

Figure S19. Observing of organoids under bright field.

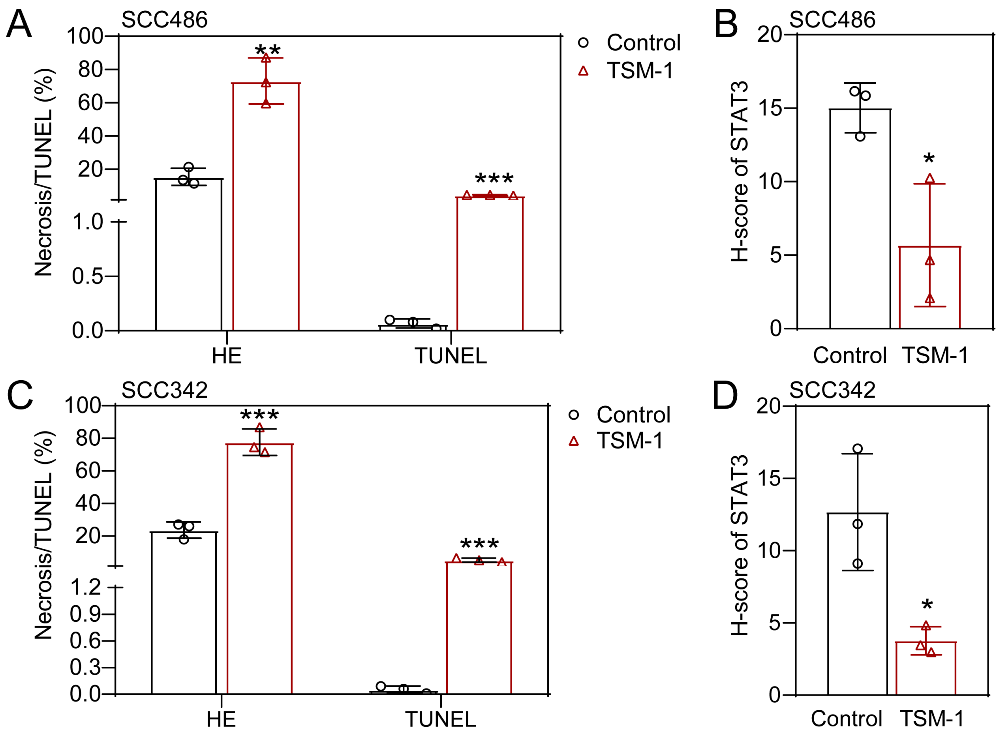

Figure S20. TSM-1 decreased STAT3 protein and induced tumor necrosis in PDX models. (A) PDX SCC486. (B) STAT3 expression in PDX SCC486. (C) PDX SCC342. (D) STAT3 expression in PDX SCC342. n=3 mice, statistical significance: **p*<0.05, ***p*<0.01, ****p*<0.001 versus the control group. P values are from Unpaired t test (A, B, C, D).

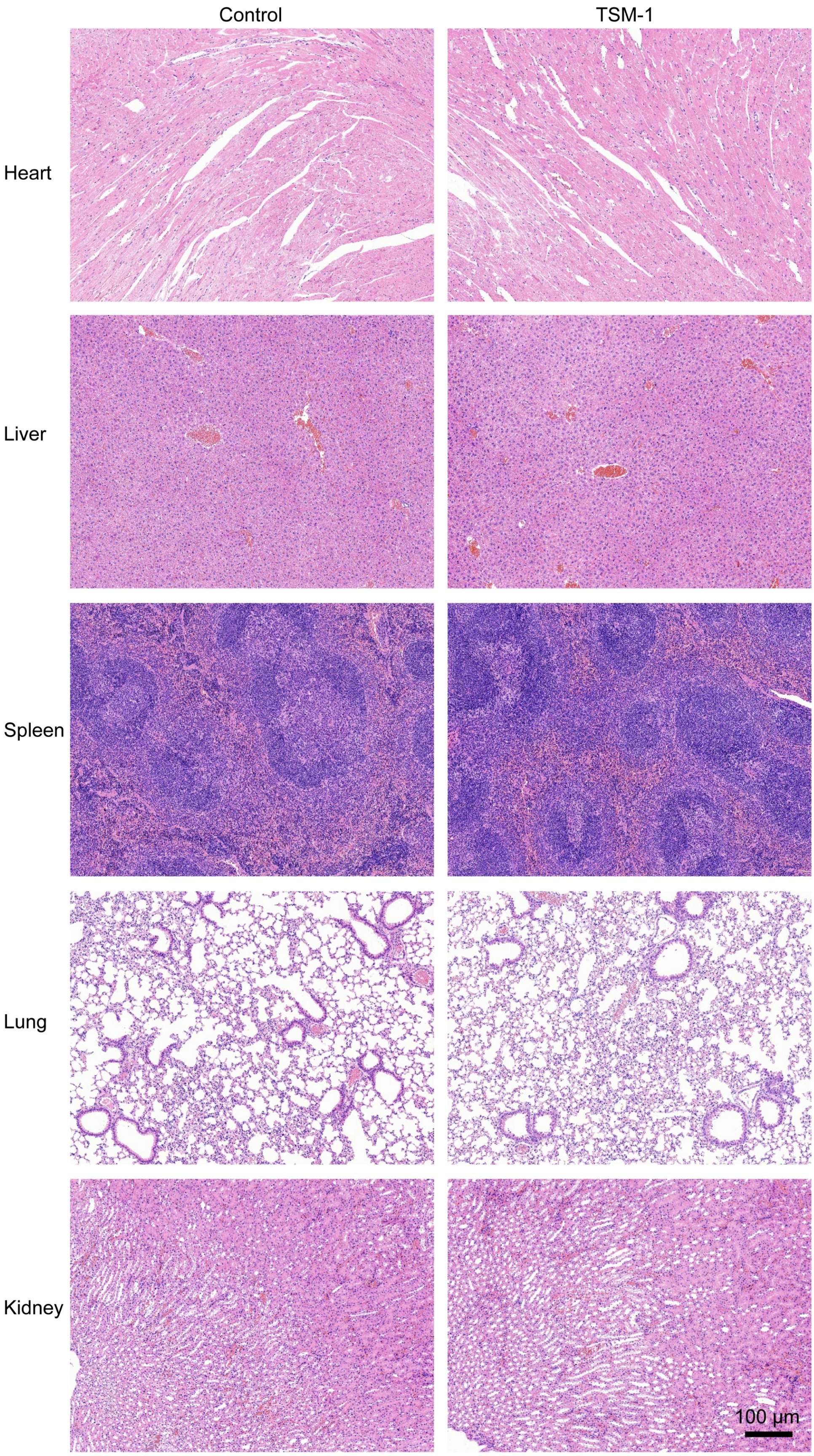

Figure S21. Immunohistochemical staining of major organs in SCC342 model.

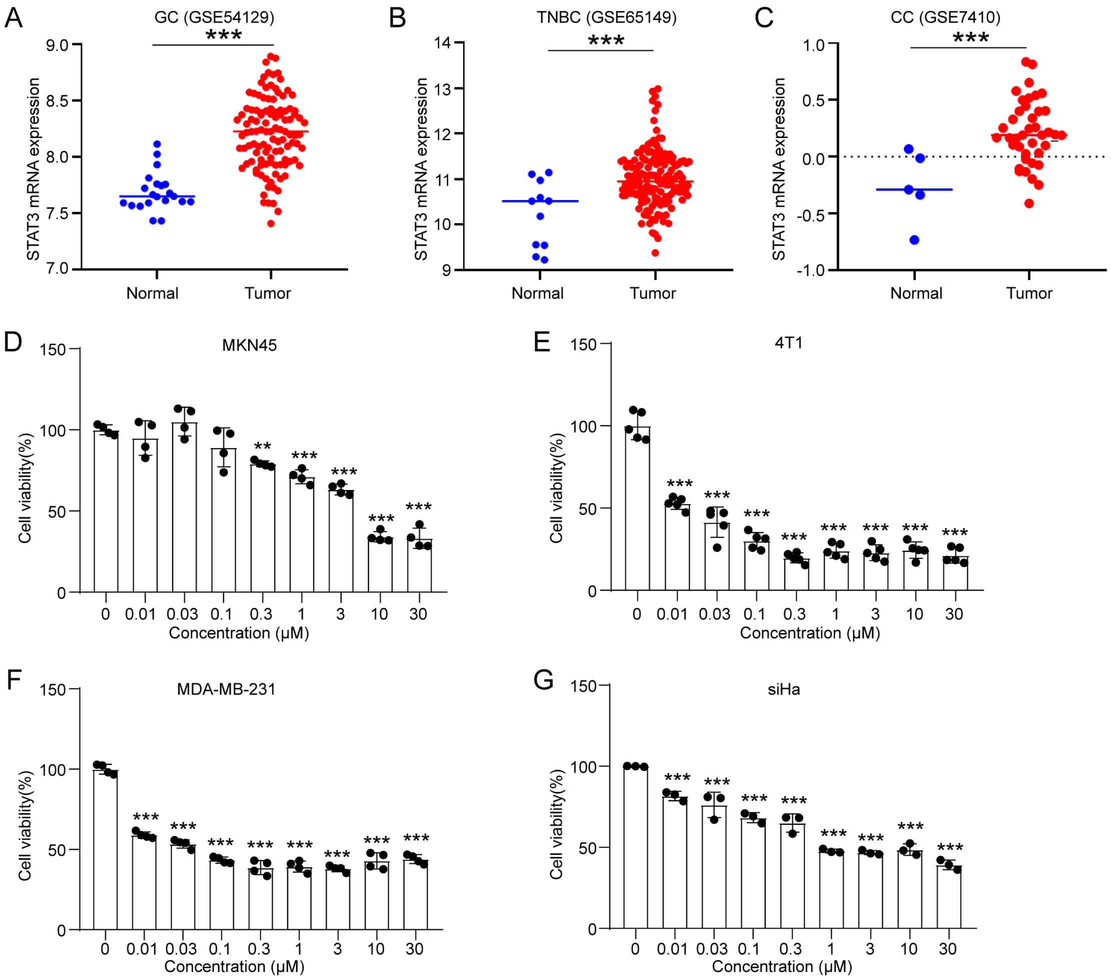

Figure S22. Expression of STAT3 and anti-tumor effects of TSM-1. STAT3 expression was significantly increased in gastric carcinoma (GC) (A), triple negative breast cancer (TNBC) (B), and cervical cancer (CC) (C) patients. cells proliferation was detected using CCK-8 assays after treatment with TSM-1 for 48 h in MKN45 (D, n=4 replicates), 4T1(E, n=5 replicates), MDA-MB-231(F, n=4 replicates), and siHa (G, n=3 replicates)cells. Asterisks indicate: ***p* < 0.01, ****p* < 0.001 when compared to the control group. P values are from Unpaired t test (A, B, C) or ordinary one-way ANOVA with Dunnett's multiple comparison test (D, E, F, G).

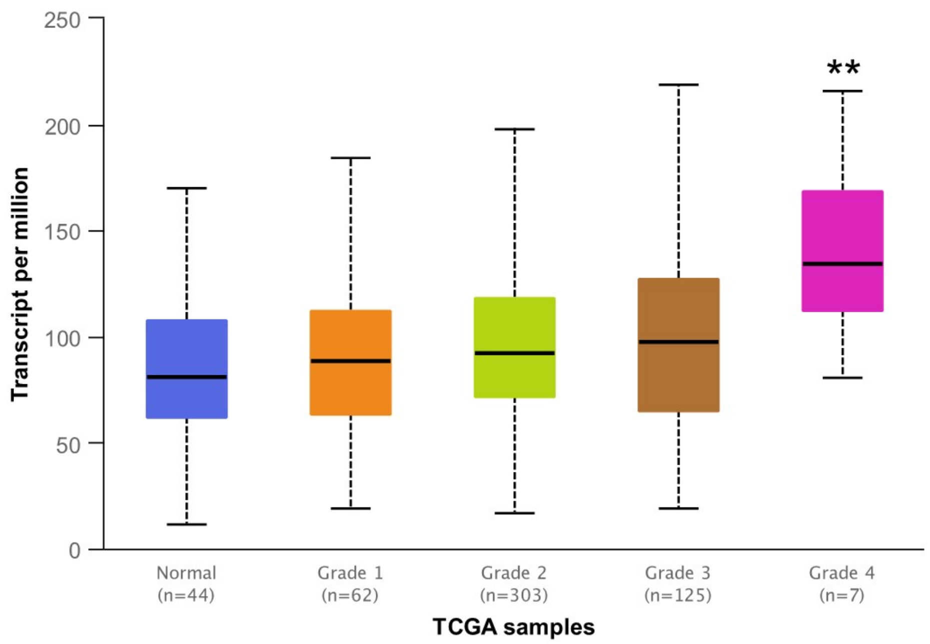

Figure S23. Expression of STAT3 in HNSCC based on tumor grade according to TCGA database. P values are from ordinary one-way ANOVA with Dunnett's multiple comparison test.

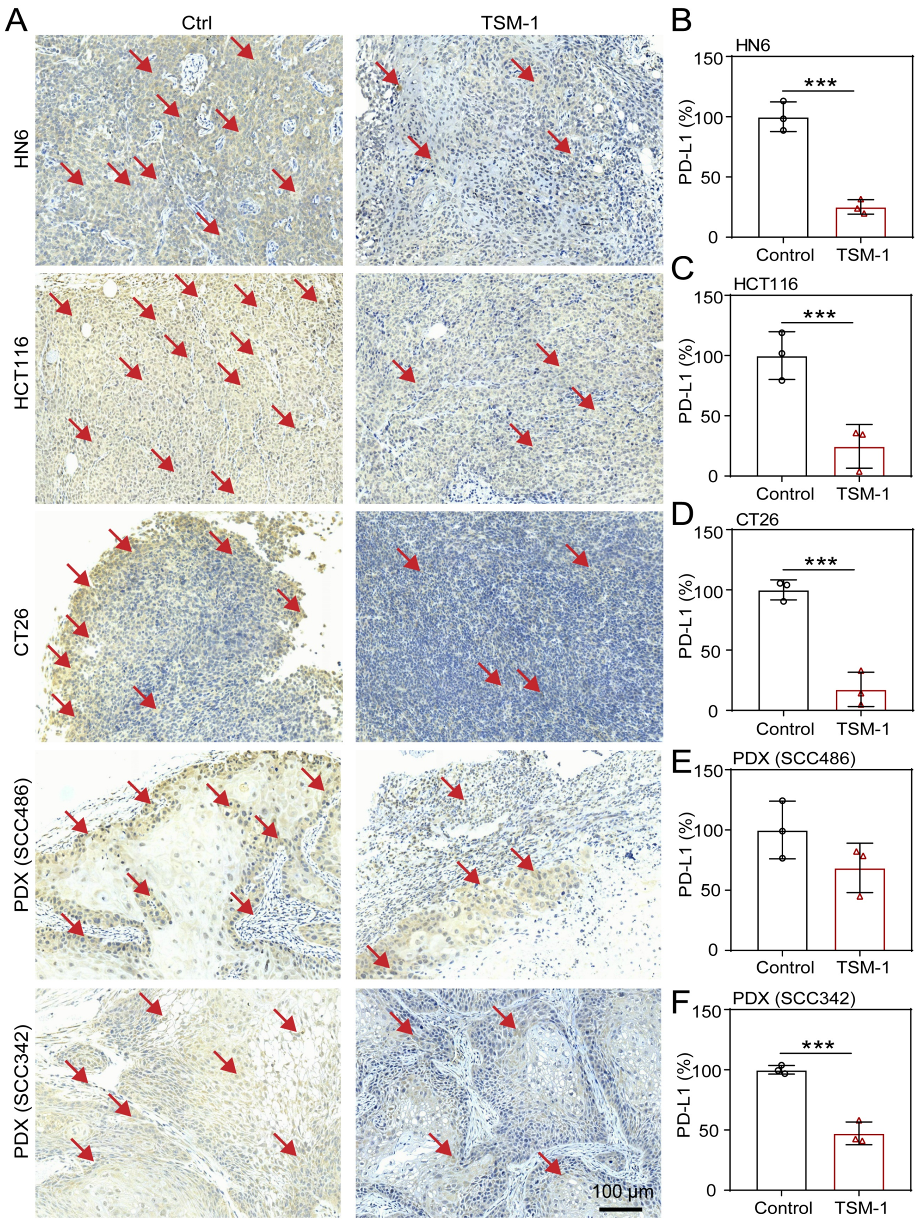

Figure S24. TSM-1 decreased PD-L1 protein in tumor tissues. (A) Immunohistochemical staining of tumor tissues. The statistical analysis results were shown in (B-F) (n=3 mice). Statistical significance: ****p*<0.001 versus the control group. P values are from Unpaired t test (B-F).

Table S1. IC_{50} of HNSCC and CRC cells when treated with TSM-1 for 48 h .

Cell line (HNSCC)	IC_{50} (μM)	Cell line (CRC)	IC_{50} (μM)
CAL27	0.301	HCT116	2.212
CAL33	0.292	DLD-1	5.776
HN6	0.297	HT29	0.297
HN30	1.374	SW620	＞30
PE/CA-PJ15	0.376		

Table S2. The sequence of oligonucleotides

Name	Sequence	
PC	sense(5'-3')	UGACCUCAACUACAUGGUUTT
	antisense(5'-3')	AACCAUGUAGUUGAGGUCATT
NC	sense(5'-3')	UUCUCCGAACGUGUCACGUTT
	antisense(5'-3')	ACGUGACACGUUCGGAGAATT
siRNA (1729)	sense(5'-3')	GGGACCUGGUGUGAAUUAUTT
	antisense(5'-3')	AUAAUUCACACCAGGUCCCTT
siRNA (1878)	sense(5'-3')	GGUACAUCAUGGGCUUUAUTT
	antisense(5'-3')	AUAAAGCCCAUGAUGUACCTT
siRNA (1272)	sense(5'-3')	CCCGGAAAUUUAACAUUCUTT
	antisense(5'-3')	AGAAUGUUAAAUUUCCGGGTT

Table S3. The sequence of plasmids CRBN-EGFP-HOTag3 and STAT3-EGFP-HOTag6

**Name**	**Sequence**
CRBN-EGFP-HOTag3	atggccggcgaaggagatcagcaggacgctgcgcacaacatgggca accacctgccgctcctgcctgcagagagtgaggaagaagatgaaatgg aagttgaagaccaggatagtaaagaagccaaaaaaccaaacatcata aattttgacaccagtctgccgacatcacatacatacctaggtgctgatatg gaagaatttcatggcaggactttgcacgatgacgacagctgtcaggtgat tccagttcttccacaagtgatgatgatcctgattcccggacagacattacct cttcagctttttcaccctcaagaagtcagtatggtgcggaatttaattcagaa agatagaacctttgctgttcttgcatacagcaatgtacaggaaagggaag cacagtttggaacaacagcagagatatatgcctatcgagaagaacagg attttggaattgagatagtgaaagtgaaagcaattggaagacaaaggttc aaagtccttgagctaagaacacagtcagatggaatccagcaagctaaa gtgcaaattcttcccgaatgtgtgttgccttcaaccatgtctgcagttcaatta gaatccctcaataagtgccagatatttccttcaaaacctgtctcaagagaa gaccaatgttcatataaatggtggcagaaataccagaagagaaagtttc attgtgcaaatctaacttcatggcctcgctggctgtattccttatatgatgctg

STAT3-EGFP-HOTag6	agaccttaatggacagaatcaagaaacagctacgtgaatgggatgaaa<br>atctaaaagatgattctcttccttcaaatccaatagatttttcttacagagtag<br>ctgcttgtcttcctattgatgatgtattgagaattcagctccttaaaattggcag<br>tgctatccagcgacttcgctgtgaattagacattatgaataaatgtacttccc<br>tttgctgtaaacaatgtcaagaaacagaaataacaaccaaaaatgaaat<br>attcagtttatccttatgtgggccgatggcagcttatgtgaatcctcatggata<br>tgtgcatgagacacttactgtgtataaggcttgcaacttgaatctgataggc<br>cggccttctacagaacacagctggtttcctgggtatgcctggactgttgccc<br>agtgtaagatctgtgcaagccatattggatggaagtttacggccaccaaa<br>aaagacatgtcacctcaaaaattttggggcttaacgcgatctgctctgttgc<br>ccacgatcccagacactgaagatgaaataagtccagacaaagtaatac<br>tttgcttgatggtgagcaagggcgaggagctgttcaccggggtggtgccc<br>atcctggtcgagctggacggcgacgtaaacggccacaagttcagcgtgt<br>ccggcgagggcgagggcgatgccacctacggcaagctgaccctgaag<br>ttcatctgcaccaccggcaagctgcccgtgccctggcccaccctcgtgac<br>caccctgacctacggcgtgcagtgcttcagccgctaccccgaccacatg<br>aagcagcacgacttcttcaagtccgccatgcccgaaggctacgtccagg<br>agcgcaccatcttcttcaaggacgacggcaactacaagacccgcgccg<br>aggtgaagttcgagggcgacaccctggtgaaccgcatcgagctgaagg<br>gcatcgacttcaaggaggacggcaacatcctggggcacaagctggagt<br>acaactacaacagccacaacgtctatatcatggccgacaagcagaag<br>aacggcatcaaggtgaacttcaagatccgccacaacatcgaggacgg<br>cagcgtgcagctcgccgaccactaccagcagaacacccccatcggcg<br>acggccccgtgctgctgcccgacaaccactacctgagcacccagtccg<br>ccctgagcaaagaccccaacgagaagcgcgatcacatggtcctgctgg<br>agttcgtgaccgccgccgggatcactctcggcatggacgagctgtacaa<br>gggcgaaattgcgaaaagcctgaaagaaattgcgaaaagcctgaaag<br>aaattgcgtggagcctgaaagaaattgcgaaaagcctgaaaggctaa<br>atggcccaatggaatcagctacagcagcttgacacacggtacctggag<br>cagctccatcagctctacagtgacagcttcccaatggagctgcggcagttt<br>ctggccccttggattgagagtcaagattgggcatatgcggccagcaaag<br>aatcacatgccactttggtgtttcataatctcctgggagagattgaccagca<br>gtatagccgcttcctgcaagagtcgaatgttctctatcagcacaatctacg<br>aagaatcaagcagtttcttcagagcaggtatcttgagaagccaatggag<br>attgcccggattgtggcccggtgcctgtgggaagaatcacgccttctaca<br>gactgcagccactgcggcccagcaaggggggccaggccaaccacccc<br>acagcagccgtggtgacggagaagcagcagatgctggagcagcacct<br>tcaggatgtccggaagagagtgcaggatctagaacagaaaatgaaagt<br>ggtagagaatctccaggatgactttgatttcaactataaaaccctcaagag<br>tcaaggagacatgcaagatctgaatggaaacaaccagtcagtgaccag<br>gcagaagatgcagcagctggaacagatgctcactgcgctggaccagat<br>gcggagaagcatcgtgagtgagctggcggggcttttgtcagcgatggag<br>tacgtgcagaaaactctcacggacgaggagctggctgactggaagagg<br>cggcaacagattgcctgcattggaggcccgcccaacatctgcctagatc<br>ggctagaaaactggataacgtcattagcagaatctcaacttcagacccgt<br>caacaaattaagaaactggaggagttgcagcaaaaagtttcctacaaa<br>ggggaccccattgtacagcaccggccgatgctggaggagagaatcgtg<br>gagctgtttagaaacttaatgaaaagtgcctttgtggtggagcggcagcc<br>ctgcatgcccatgcatcctgaccggcccctcgtcatcaagaccggcgtcc<br>agttcactactaaagtcaggttgctggtcaaattccctgagttgaattatcag

cttaaaattaaagtgtgcattgacaaagactctggggacgttgcagctctc
agaggatcccggaaatttaacattctgggcacaaacacaaaagtgatg
aacatggaagaatccaacaacggcagcctctctgcagaattcaaacac
ttgaccctgagggagcagagatgtgggaatgggggccgagccaattgt
gatgcttccctgattgtgactgaggagctgcacctgatcacctttgagacc
gaggtgtatcaccaaggcctcaagattgacctagagacccactccttgcc
agttgtggtgatctccaacatctgtcagatgccaaatgcctgggcgtccat
cctgtggtacaacatgctgaccaacaatcccaagaatgtaaactttttac
caagcccccaattggacctgggatcaagtggccgaggtcctgagctggc
agttctcctccaccaccaagcgaggactgagcatcgagcagctgactac
actggcagagaaactcttgggacctggtgtgaattattcagggtgtcagat
cacatgggctaaattttgcaaagaaaacatggctggcaagggcttctcctt
ctgggtctggctggacaatatcattgaccttgtgaaaaagtacatcctggc
cctttggaacgaagggtacatcatgggctttatcagtaaggagcgggag
cgggccatcttgagcactaagcctccaggcaccttcctgctaagattcagt
gaaagcagcaaagaaggaggcgtcactttcacttgggtggagaagga
catcagcggtaagacccagatccagtccgtggaaccatac
acaaagcagcagctgaacaacatgtcatttgctgaaatcatcatgggcta
taagatcatggatgctaccaatatcctggtgtctccactggtctatctctatcc
tgacattcccaaggaggaggcattcggaaagtattgtcggccagagagc
caggagcatcctgaagctgacccaggtagcgctgccccatacctgaag
accaagtttatctgtgtgacaccaacgacctgcagcaataccattgacctg
ccgatgtcccccgcactttagattcattgatgcagtttggaaataatggtg
aaggtgctgaaccctcagcaggagggcagtttgagtccctcacctttgac
atggagttgacctcggagtgcgctacctcccccatgatggtgagcaagg
gcgaggagctgttcaccggggtggtgcccatcctggtcgagctggacgg
cgacgtaaacggccacaagttcagcgtgtccggcgagggcgagggcg
atgccacctacggcaagctgaccctgaagttcatctgcaccaccggcaa
gctgcccgtgccctggcccaccctcgtgaccaccctgacctacggcgtg
cagtgcttcagccgctaccccgaccacatgaagcagcacgacttcttca
agtccgccatgcccgaaggctacgtccaggagcgcaccatcttcttcaa
ggacgacggcaactacaagacccgcgccgaggtgaagttcgagggc
gacaccctggtgaaccgcatcgagctgaagggcatcgacttcaaggag
gacggcaacatcctggggcacaagctggagtacaactacaacagcca
caacgtctatatcatggccgacaagcagaagaacggcatcaaggtgaa
cttcaagatccgccacaacatcgaggacggcagcgtgcagctcgccga
ccactaccagcagaacacccccatcggcgacggccccgtgctgctgcc
cgacaaccactacctgagcacccagtccgccctgagcaaagacccca
acgagaagcgcgatcacatggtcctgctggagttcgtgaccgccgccgg
gatcactctcggcatggacgagctgtacaagaccctgcgcgaaattgaa
gaactgctgcgcaaaattattgaagatagcgtgcgcagcgtggcggaac
tggaagatattgaaaaatggctgaaaaaaattTGA
